# Genome Mapping and Molecular Breeding of Tomato

**DOI:** 10.1155/2007/64358

**Published:** 2007-08-28

**Authors:** Majid R. Foolad

**Affiliations:** Department of Horticulture and The Intercollege Graduate Degree Programs in Genetics and Plant Biology, The Pennsylvania State University, University Park, PA 16802, USA

## Abstract

The cultivated tomato, *Lycopersicon esculentum*, is the second most consumed vegetable worldwide and a well-studied crop species in terms of genetics, genomics, and breeding. It is one of the earliest crop plants for which a genetic linkage map was constructed, and currently there are several molecular maps based on crosses between the cultivated and various wild species of tomato. The high-density molecular map, developed based on an *L. esculentum* × *L. pennellii* cross, includes more than 2200 markers with an average marker distance of less than 1 cM and an average of 750 kbp per cM. Different types of molecular markers such as RFLPs, AFLPs, SSRs, CAPS, RGAs, ESTs, and COSs have been developed and mapped onto the 12 tomato chromosomes. Markers have been used extensively for identification and mapping of genes and QTLs for many biologically and agriculturally important traits and occasionally for germplasm screening, fingerprinting, and marker-assisted breeding. The utility of MAS in tomato breeding has been restricted largely due to limited marker polymorphism within the cultivated species and economical reasons. Also, when used, MAS has been employed mainly for improving simply-inherited traits and not much for improving complex traits. The latter has been due to unavailability of reliable PCR-based markers and problems with linkage drag. Efforts are being made to develop high-throughput markers with greater resolution, including SNPs. The expanding tomato EST database, which currently includes ∼214 000 sequences, the new microarray DNA chips, and the ongoing sequencing project are expected to aid development of more practical markers. Several BAC libraries have been developed that facilitate map-based cloning of genes and QTLs. Sequencing of the euchromatic portions of the tomato genome is paving the way for comparative and functional analysis of important genes and QTLs.

## 1. INTRODUCTION

### 1.1. Economic importance

The cultivated tomato, *Lycopersicon esculentum* Mill., a fruit that is often treated as a vegetable, is widely grown around the world and constitutes a major agricultural industry. Worldwide, it is the second most consumed vegetable after potato (FAOSTAT 2005; http://faostat.fao.org) and unquestionably the most popular garden crop. In the U.S., it is the third most economically important vegetable (with a total farm value of $2.062 billion) following potato ($2.564 billion) and lettuce ($2.064 billion) (USDA 2005; http://www.usda.gov/nass/pubs/agr05/agstats2005.pdf). In addition to tomatoes that are eaten directly as raw vegetable or added to other food items, a variety of processed products such as paste, whole peeled tomatoes, diced products, and various forms of juice, sauces, and soups have gained significant acceptance. There are more varieties of tomato sold worldwide than any other vegetable. Although a tropical plant, tomato is grown in almost every corner of the world from the tropics to within a few degrees of the Arctic Circle. It is grown in greenhouses where outdoor production is restricted due to cool temperatures. Major tomato producing countries in descending orders include China, USA, India, Turkey, Egypt, and Italy (http://faostat.fao.org). Other leading countries include Spain, Brazil, Iran, Mexico, Greece, and Russia. In North America, production occurs in the U.S., Canada, and Mexico, comprising a total of 310 000 ha. In 2004, the total harvested area in the U.S. was estimated to be 170 808 ha (50 560 ha fresh market and 120 248 ha processing tomatoes) with a total farm value of *∼*$2.06 billion ($1.34 billion fresh market and $0.72 billion processing) (USDA 2005; http://www.nass.usda.gov:8080/QuickStats/index2.jsp). California and Florida are by far the leading producers of processing and fresh market tomatoes, respectively (USDA 2005). Worldwide, tomatoes are an important part of a diverse and balanced diet [1]. The tomato does not rank high in nutritional value; one medium fresh tomato (135 g) provides 47% RDA of vitamin C, 22% RDA vitamin A, and 25 calories. However, by virtue of volume consumed, it contributes significantly to the dietary intake of vitamins A and C as well as essential minerals and other nutrients. In the U.S. diet, for example, tomato ranks first among all fruits and vegetables as a source of vitamins and minerals [2] and phenolic antioxidants [3]. Also, fresh and processed tomatoes are the richest sources of the anti-oxidant lycopene [4], which arguably protects cells from oxidants that have been linked to cancer [5].

### 1.2. Botanical description

Tomato belongs to the nightshade family Solanaceae, which is in division Magnoliophyta, class Magnoliopsida, subclass Asteridae, order Solanales, and suborder Solanineae. The extremely diverse and large Solanaceae family is believed to consist of 96 genera and over 2800 species in three subfamilies, Solanoideae (in which *Lycopersicon* belongs), Cestroideae, and Solanineae [6, 7]. Solanaceae is one of the economically most economically important families of angiosperms and contains many of the commonly cultivated plants, including potato tomato, pepper, eggplant, petunia, and tobacco. This family is the most variable of all crop species in terms of agricultural utility, the 3rd most economically important crop family, exceeded only by the grasses and legumes, and the most valuable in terms of vegetable crops [8]. Among all plant families, members of the Solanaceae are extremely diverse in terms of growth habit (from trees to small annual herbs), habitat (from deserts to the wettest tropical rain forest), and morphology [6]. Many Solanaceous species have played important roles as model plants, including tomato, potato, pepper, tobacco, and petunia.

The tomato genus *Lycopersicon* is one of the smallest genera in Solanaceae, though the centerpiece in the family for genetic and molecular research. It is the closest to the genus *Solanum* (nightshade), an association which originally led people to believe tomato was poisonous [9]. The cultivated tomato was originally named *Solanum lycopersicum* by Linnaeus [10]. In 1754, Miller separated tomatoes and designated the genus *Lycopersicon* and the species *esculentum* for the cultivated tomato [11]. This helped with the acceptability of tomato as a food. The genus *Lycopersicon* was initially distinguished from the genus *Solanum* by its distinct characteristics of anthers and leaves. While *Lycopersicon* has anthers that dehisce laterally, and leaves that are mostly pinnate or pinnatifid, *Solanum* has anthers that dehisce from the terminal ends and leaves that tend to be simple. The *Solanum* species most closely related to and with some level of difficulty crossable with *Lycopersicon* are *S. juglandifolium* Dun., *S. ochranthum* Dun., *S. lycopersicoides* Dun., and *S. rickii* Corr. [12–14].

Phylogenetic relationships between *Solanum* and *Lycopersicon* have been the subject of a great debate for a long time, with many Solanaceae researchers recognizing *Lycopersicon* as a distinct genus while others suggesting its merger with *Solanum*. More recently, based on much molecular and morphological information, a new taxonomic classification of tomato and readoption of *S. lycopersicum* for the cultivated tomato have been suggested [6, 15–18]. The other species of *Lycopersicon* have also been assigned or reassigned to *Solanum* [16, 19–21]. In this review, however, due to the use and citation of numerous historical references and in order to be consistent with much of the literature, Miller's classification [22] is followed.

### 1.3. Genetic variation

In addition to the cultivated species *L. esculentum* and its wild form *L. esculentum* var. *cerasiforme* (Dun.) Gray (wild cherry), there are eight related wild species, including *L. pimpinellifolium* (Jusl.) Mill. (currant tomato), *L. cheesmanii* Riley, *L. chmielewskii* Rick, Kes., Fob. and Holle, *L. chilense* Dun., *L. parviflorum* Rick, Kes., Fob. and Holle, *L. peruvianum* (L.) Mill., *L. hirsutum* Humb. and Bonpl., and *L. pennellii* (Corr.) D'Arcy [23, 24]. All species are native to Western South America, mainly Peru. Only the cultivated species and wild cherry are found outside this range and are common throughout many parts of the world, especially in Mesoamerica and the Caribbean [25]. However, the natural habitat of *Lycopersicon* is highly variable, from very dry to very wet and from coastal to mountainous areas of more than 3300 m elevation [14]. This diversity in habitat has undoubtedly contributed to the great variation that can be found in *Lycopersicon*.

All species within *Lycopersicon* produce perfect, hermaphrodite flowers. A complete range of mating systems is found, from autogamous *L. cheesmanii* and *L. parviflorum* to obligately outcrossed self-incompatible biotypes of *L. chilense, L. hirsutum, L. peruvianum,* and *L. pennellii* [26]. Self fertility with various degrees of facultative outcrossing is found in *L. chmielewskii, L. esculentum, L. pimpinellifolium,* and the self-compatible biotypes of *L. hirsutum* and *L. pennellii* [27]. All tomato species are diploid (2*n* = 2*x = 24*) and are similar in chromosome number and structure [28]. The nine species have been grouped into two intracrossable, interincrossable groups (or complexes): the “*esculentum* complex,” including *L. esculentum, L. esculentum* var. * cerasiforme*, *L. pimpinellifolium, L. cheesmanii* (including *L. cheesmanii* f. *minor*), *L. chmielewskii, L. parviflorum, L. hirsutum* (including f. *typicum* and f. *glabratum*) and *L. pennellii*, and the “*peruvianum* complex” including *L. peruvianum* and *L. chilense* [12, 29, 30]. All species within the *esculentum* complex can be hybridized with the cultivated tomato and most (except *L. hirsutum* f. *typicum* and some *L. pennellii*) are self compatible [12, 23, 31]. They have yellow flowers and the stamens are joined to produce an anther cone. Fruit color varies depending on the species, from red to orange to yellow to green. Several members of the *esculentum* complex have provided sources of pest resistance and other desirable characteristics in the cultivated tomato, as discussed below. The two species within the *peruvianum* complex are extremely diverse and represent a wealth of characteristics, which are potentially valuable for crop improvement. These species, which are mostly self incompatible and produce green fruit, have been rather partial in their usefulness to cultivated forms due to various barriers present in sexual hybridization and gene transfer. However, they can be hybridized with members of the *esculentum* complex by the application of techniques such as embryo rescue [23, 24, 32] or by the use of pollen mixtue (with tomato pollen) when fertilizing tomato plants [33]. There are documented examples of crosses with *peruvianum* complex which have been utilized in tomato breeding, including transfer of tobacco mosaic virus and nematode resistance [30, 34].

The cultivated tomato has limited variability, largely because of several population bottlenecks in the forms of founder events and natural and artificial selections that occurred during domestication and evolution of modern cultivars [25]. For example, tomatoes that were first introduced to Europe by Spanish explorers furnished the entire genetic base for the modern cultivars and consequently the current European and U.S. cultivars are highly similar to each other. It is estimated that only *∼*5% of the total genetic variation within *Lycopersicon* can be found within *L. esculentum* [35, 36] and genes for many desirable agricultural characteristics do not exist in this species. The related wild tomato species, however, are a rich source of desirable genes and characteristics for crop improvement, though they remain largely under exploited. The species with the greatest variability are *L. chilense, L. hirsutum, L. peruvianum, * and *L. pennellii* whereas the least variable species are *L. cheesmanii* and *L. pimpinellifolium* [35, 37].

During the past 70 years wild species of tomato have been utilized in breeding programs to improve the cultivated tomato [24, 27, 32]. For example, much of the disease resistance in most commercial cultivars has been derived from the related wild species. In fact, the cultivated tomato is a prime example of a crop plant that has benefited significantly from exotic germplasm introgressions, probably more so than any other crop species. Furthermore, the great diversity available in tomato wild species promises many more advances in this area. Numerous wild accessions have been identified with desirable horticultural characteristics such as high fruit quality and tolerance to abiotic stresses. Recent advancements in molecular markers and marker-assisted selection (MAS) technology are expected to make tomato improvement via introgression from wild species more feasible. It is estimated that over 62 800 accessions of the cultivated and wild species of tomato (mostly *L. esculentum* accessions) are maintained in genebanks around the world [38], including those in the Asian Vegetable Research and Development Center (AVRDC) at Tainan, Taiwan, China, the United States Department of Agriculture (USDA), Plant Genetic Resources Unit at Geneva (PGRU), Ny, USA, and the CM Rick Tomato Genetics Resource Center (TGRC), University of California, Davis, Calif, USA. The TGRC (http://tgrc.ucdavis.edu) is known to maintain the largest collection of tomato wild species while PGRU has a large collection of open-pollinated cultivars. Good collections of tomato germplasm are also maintained in The Netherlands (IVT), Russia (VIR), Japan (NIAS), Peru (DHUNA), and Cuba (INIFAT) [39]. In addition to the wild and cultivated accessions, there are thousands of tomato monogenic stocks and mutants that have been phenotypically characterized and cataloged (http://tgrc.ucdavis.edu; http://www.zamir.sgn.cornell.edu).

### 1.4. Domestication and crop production

Among the nine *Lycopersicon* species, only *L. esculentum* has become a domesticated crop though *L. pimpinellifolium* (with fruit diameter *∼*1 cm) is also casually planted for consumption [9]. According to [25], domestication of *L. esculentum* was accompanied by a transition from exerted to inserted stigmas, and consequently changing from facultative outcrossing to enforced inbreeding. As a result of this autogamy, most accessions within the cultivated species, including the common fresh market and processing tomatoes, landraces, primitive cultivars, and the wild cherry are essentially pure lines. Fruits of the cultivated species come in a wide range of shapes, sizes, and colors. Some may be globe, round, flattened, oval, heart, or elongated shaped. Their colors may be red, pink, golden, yellow, striped, purple, green, or white [30, 34]. The average weight for the garden tomato used for slicing is 4 to 6 oz., but some varieties such as Giant Heirloom may weigh up to 2 pounds [40]. It is arguably accepted that the wild cherry (*L. esculentum* var. *cerasiforme*, with fruit diameter of *∼*1.5–3 cm) is the immediate progenitor of the cultivated tomato though *L. pimpinellifolium* is also a likely candidate [25, 41]. The isozyme and molecular phylogenetic and diversity studies have not clarified this issue [35, 36, 42].

The cultivated tomato is thought to have originated in the new world, since all of its related wild species are native to the Andean region now encompassed by parts of Peru, Chile, Colombia, Ecuador, and Bolivia [9, 25, 41]. Some distinct relatives (e.g., *L. pennellii*) are also found among the flora of the Galapagos Islands. Although Peru was earlier widely accepted as the center of domestication, “the bulk of the historical, linguistic, archaeological, and ethno-botanical evidence favors Mexico as the source of the cultivated tomatoes” [25]. Also, despite the wide distribution of the genus in Andean region, Mexico has been considered the most likely center of domestication of tomato. The name “tomato” is derived from the Spanish “tomate” which in turn is derived from the Mexican Nahuatl name “tomatl,” which actually means tomatillo, and applied both to the tomato as we know it and the husk tomato, genus *Physalis* [9]. It is not known exactly when domestication of tomatoes occurred, however, by the time the Spanish conquered Mexico in 1523, they were already domesticated [9]. A comparison of hereditary enzyme variants reveals much greater similarity between the older European cultivars and the primitive cultivars and cherry tomatoes of Mexico and Central America than between the European cultivars and the primitive plants of the Andean region [25, 43]. The first record of tomatoes in Europe is credited to descriptions published in 1554 by Italian herbalist Pier Andrea Mattioli. The plant was first known as *pomi d'oro*, *mala aurea* (golden apple), *poma amoris* (love apple), and garden apple [9]. These and equivalent names persisted well into the 19th century. Tomato first appeared in a cookbook in 1692, nearly two hundred years after Columbus headed for the new world. However, even then, because of the persistent superstitions regarding the poisonous nature of the tomato, it was remarkably slow to gain acceptance except as an ornamental, a medicinal, or a curiosity [9]. Such unfounded superstitions persisted into the 19th century in many parts of the world, including North America, to which the plant had been taken by immigrants in the 1600s and early 1700s [9]. Commercial production of tomato in a small scale in the U.S. began in 1847 at Lafayette College at Easton, Pa, which grew to become a major vegetable crop in the mid 20th century.

### 1.5. A model organism

Tomato has been an excellent model system for both basic and applied plant research. This has been due to many reasons [44], including ease of culture under a wide range of environments, short life cycle, photoperioid insensitivity, high self fertility and homozygosity, great reproductive potential, ease of controlled pollination and hybridization, diploid species with a rather small genome (*∼*0.95 pg/1C, 950 Mbp) [45, 46], lack of gene duplication, amenability to asexual propagation and whole plant regeneration [47, 48], the ability to develop haploids [49], and availability of a wide array of mutants [50] and genetic stocks (including wild species; http://tgrc.ucdavis.edu; http://www.sgn.cornell.edu). Tomato's regenerative plasticity also allows easy grafting, an attribute that facilitates certain developmental and practical studies. Recent availability of high molecular weight insert genomic libraries, including both YAC [51] and BAC [52, 53] libraries, has facilitated map-based or positional cloning. Furthermore, members of *Lycopersicon* are easily transformed, and transgenic tomatoes are routinely produced using cocultivation with *Agrobacterium tumefaciens* [47, 54]. Tomato was the first food crop in the U.S. for which a genetically engineered variety was marketed [55] and also for which a disease resistance gene was positionally coloned [56, 57]. Currently, the euchromatic portions of the 12 tomato chromosomes are being sequenced, which will make tomato even more of ideal crop plant system for genomic studies.

### 1.6. Breeding objectives and previous achievements

Breeding new cultivars of tomato with improved characteristics started more than 200 years ago in Europe (mainly in Italy). In the U.S., however, tomato breeding started only a little over a century ago and AW Livingston is recognized as the first tomato breeder in 1870s [30, 34]. Until 1950s, tomato breeding included development of multipurpose cultivars to meet several needs, including fresh market and processing industries. Subsequently, breeding objectives have depended upon method of cultur, that is, field or greenhouse grown, and whether the product has to be used fresh or processed [30, 34]. Today, fresh market and processing cultivars are quite distinct, largely as a result of the different quality requirements for intended use. However, the universal goal of tomato breeding for both fresh market and processing purposes has been to increase fruit yield per unit area. Other essential characteristics common to both industries include disease resistance, broad adaptability, earliness in maturity, ability to set fruit at adverse temperatures, resistance to rain-induced cracking, tolerance to major ripe-fruit rots, adequate vine cover, fruit firmness, and several other fruit quality characteristics. Specific traits needed in processing cultivars include compact, determinate plant habit and concentrated flowering and fruit set suitable for once-over machine harvest, ease of fruit separation from the vine (*jointless* characteristic), and specific fruit quality characteristics such as color, pH, total acidity, soluble solids, total solids, and viscosity (consistency). Specific traits of interest in fresh market cultivars include large, round fruit with adequate firmness and shelf-life, uniform fruit size, shape and color, appearance, freedom from external blemishes or abnormalities, texture, taste and flavor [30, 34]. Currently, in the U.S. much of the tomato breeding work is conducted in private sector (seed companies). However, a few major public tomato breeding programs include those in the University of Florida (JW Scott, fresh market), North Carolina State University (RG Gardner, fresh market), Ohio State University (DM Francis, processing), Pennsylvania State University (Foolad, fresh market and processing) and Cornell University (MA Mutschler). In what follows, some of the major breeding achievements in different areas are briefly discussed.

**Yield.**

Unless a new cultivar has a yield potential equal to or exceeding that of current cultivars, it generally cannot be successful even if it may contain other improved characteristics. Because selection for yield *per se* is seldom very effective, breeders often define individual components that contribute to yield and emphasize selection for those attributes. Breeding for improved fruit yield in tomato has been very successful. For example, between 1920s and 1990s fruit yield of processing tomato cultivars in the U.S. increased from 10.1 tons/ha to 72.4 tons/ha, a 7.2-fold increase [58]. A recent statistic by the USDA indicated processing tomato yield of *∼*102 tons/ha in the U.S. in 2004 (http://www.nass.usda.gov:8080/QuickStats/index2.jsp). It is estimated that on an average about half of the increase in crop productivity has been due to cultivar improvement through plant breeding [59]. Greater farming inputs and advancements in cultural practices are considered other major causes of increases in productivity. Even today, increased yield and quality of tomato is the universal goal of most tomato breeding programs, though this increase may be achieved by selecting for other desirable characteristics such as disease resistance, tolerance to abiotic stresses, earliness, and improved fruit sugar contents. In the fresh market tomato breeding program at the University of Florida, for example, increased yield has been achieved by breeding for heat tolerance for production under hot and humid conditions [60, 61]. Because of the difficulties associated with phenotypic selection for improved yield, more recently molecular markers have been identified for traits that are directly or indirectly related to yield in tomato.

**Disease resistance.**

Diseases are first concern to processing and fresh market tomato industries throughout the world and economic losses due to crop damge or disease control measures are significant (http://faostat.fao.org). Tomato is susceptible to over 200 diseases caused by pathogenic fungi, bacteria, viruses, or nematodes [62]. Without question, the greatest contribution of modern plant breeding to tomato improvement has been through development of cultivars with improved disease resistance. Resistance has been identified, and in many cases characterized, for more than 30 of the major tomato diseases. Most commercial cultivars possess up to 6 (in true breeding lines) or 10 (in hybrids) disease-resistance attributes. These mainly include diseases for which major resistance genes have been identified, including fusarium wilt, verticillium wilt, root-knot nematode, alternaria stem canker, gray leaf spot, and some bacterial and viral diseases. However, horizontal (a.k.a. field or polygenic) resistance has also been reported for several tomato diseases, where major genes for resistance to a particular pathogen or race are not found, such as early blight, powdery mildew, bacterial canker, and bacterial wilt. Except in a few cases (e.g., [63–69]), tomato wild species have been utilized as the source of resistance for all tomato diseases. Resistance resources have been identified in most related wild species of tomato, in particular *L. pimpinellifolium*, *L. peruvianum*, and *L. hirsutum*. For some tomato diseases, such as late blight (caused by oomycete *Phytophthora infestans*) and powdery mildew (caused by fungus *Oidium lycopersicum*), both vertical and horizontal resistances have been identified. All original characterization, disease evaluation, and incorporation of resistance genes were through phenotypic selection and traditional breeding protocols, and still today much of the disease resistance breeding in tomato is through the use of similar protocols. However, the difficulties encountered when transferring resistance from wild species to the cultivated tomato via traditional protocols have restricted transfer of resistance to many tomato elite lines. This is in addition to the lack of suitable screening facility or expertise in many tomato breeding programs to develop cultivars with multiple disease resistances. Thus, breeders have consistently sought more effective approaches for resistance breeding. During the past two decades, the use of molecular markers and MAS techniques have facilitated identification, mapping, and transferring of many disease resistance genes and quantitative trait loci (QTLs) in tomato. The use of marker technology in disease resistance breeding in tomato is becoming a routine procedure. Furthermore, breeding for disease resistance remains a major goal of most public and private tomato breeding programs as new diseases achieve significance or new races of existing pathogens become established. The ultimate goal is to eliminate or significantly reduce the use of pesticides in tomato production by the use of host resistance.

**Insect resistance.**

The cultivated tomato is subject to attack by numerous insects, including various species of mites, whiteflies, aphids, Lepidoptera (e.g., tomato fruitworm, beet armyworm, cotton bollworm, southern armyworm, soybean podworm, and Egyptian cottonworm), Coleoptera (e.g., Colorado potato beetle and tobacco flea beetle), Diptera (e.g., leafminers and fruit fly), thrips, sinkbugs, and cutworms, many of them capable of causing devastating losses. Insect resistance in tomato has received considerably less attention than disease resistance, and few commercial cultivars have been developed with specific insect resistance. However, resistance to major insect pests of tomato has been identified within the related wild species, in particular *L. hirsutum* and *L. pennellii* [34, 70–75]. *L. hirsutum*, the most notable source of arthropod resistance, occurs in two distinct forms, *L. hirsutum* f. *typicum* and *L. hirsutum* f. *glabratum* CM Mull [75], each showing resistance to at least 16 pest species [76]. Resistance to at least nine insect species has been reported in *L. pennellii*, including greenhouse whitefly, carmine and two-spotted spider mites, and the potato aphid [72]. Some insect resistance has also been reported in *L. esculentum* var. *cerasiforme*, *L. pimpinellifolium*, *L. cheesmanii* and *L. chmielewskii, L. peruvianum* and *L. chilense* [76]. Unfortunatley, most of these resources have not been characterized or utilized for insect resistance breeding, though a few inheritance studies have been undertaken [70, 77, 78]. Breeding for insect resistance in tomato has generally encountered more difficulties than breeding for disease resistance, linkage drag being a major impediment [34, 72, 79, 80]. It is expected that identification of markers associated with insect resistance and use of MAS will help alleviate some of the difficulties in developing insect resitant cultivars.

**Abiotic stress tolerance.**

Although the cultivated tomato is widely adapted to different climates, its growth and development is rather sensitive to different environmental stresses, including salinity, drought, excessive moisture, extreme temperatures, mineral toxicity and deficiency, and environmental pollution. There is limited genetic variation for abiotic stress tolerance within the cultivated species and most commercial cultivars are considered moderately to highly sensitive to different stresses. Fortunatley, sources of genetic tolerance (or resistance) to different abiotic stresses are found within the related wild species, including *L. chilense*, *L. peruvianum*, *L. pennellii*, *L. pimpinellifolium*, *L. hirsutum*, *L. cheesmanii, L. chmielewskii,* and *L. parviflorum* [81]. In addition, there are a few species within *Solanum* that exhibit tolerance to environmental stresses and which may be utilized in tomato breeding for stress tolerance. These include *S. lycopersicoides* Dun. and *S. rickii* Corr., which are more closely related to tomato and *S. juglandifolium* Dun. and *S. ochranthum* Dun., which are more distantly related [12, 13, 31, 82, 83].

Several tomato wild species have been utilized for genetic and physiological characterization of abiotic stress tolerance and for breeding purposes [60, 84–91] However, there is only few report of stress-tolerant tomatoes developed via traditional breeding protocols. This is in part due to the complexity of abiotic stress tolerance traits. A plant's response to environmental stress is modulated by many physiological and agronomical characteristics, which may be controlled by the actions of several to many genes whose expressions are influenced by various environmental factors. In addition, stress tolerance is a developmentally regulated, stage-specific phenomenon; tolerance at one stage of plant development is often not correlated with tolerance at other developmental stages [92–97]. For successful tomato production under environmental stress, tolerance may be needed at all major stages of plant development, including seed germination, the vegetative stage, and flowering and fruit production. Each developmental stage (which may be considered as a separate trait) may require a different screening procedure and simultaneous or sequential screening may be impractical or impossible. However, quantification of tolerance often poses serious difficulties. Phenotypic selection under field conditions is difficult because uncontrollable environmental factors adversely affect the precision and repeatability of such trials. There is often no reliable screening technique that could be used year after year or generation after generation. Furthermore, selection for stress tolerance using phenotypic measurements requires specialized personnel and extensive investments in field nurseries or greenhouse facilities. Thus, the challenge has been to improve the efficiency of selection and breeding for stress tolerance. For the past two decades, the identification and use of genetic markers that are associated with traits related to stress tolerance has been considered and suggested as a promising approach. Thus, rather extensive research has been conducted in tomato to identify QTLs for tolerance to different environmental stresses, as described below.

**Fruit quality.**

Fruit quality has been a major focus of most tomato breeding programs during the past century. Major fruit quality characteristics of interest to both fresh market and processing tomato industries include fruit size, shape, total solids, color, firmness, ripening, nutritional quality and flavor. Fruit total solids content is particularly important to the processing industry and probably has received more attention than any other fruit trait. The total solids of the cultivated tomato comprise 4–7.5% of its fresh weight, though this percentage can be much higher in some wild species [98, 99]. The total solids are composed of all fruit components except water and volatiles. In the cultivated tomato, the soluble (SS) and insoluble solids (ISS) account for about 75% and 25%, respectively, of the total solids [100]. Reducing sugars glucose and fructose are the major components of the SS [101]. Sucrose is also present but in very small quantities [102], although some wild species of tomato, including *L. chmielewskii* [103] and *L. hirsutum* [102], have higher concentration of sucrose. The remaining soluble solids are composed of organic acids, lipids, minerals, and pigments. The ISS include proteins, cellulose, hemicellulose, pectins, and polysaccharides, which determine fruit viscosity [34, 104, 105]. Quality of tomato juice, catsup, sauce, soup, and paste are influenced by viscosity of the product. Both SS and ISS are related to yield of concentrated tomato products, and yield and quality of certain processed products are determined by sugar contents of the fruit [105]. For tomato products that are sold on the basis of solids content, the higher the solids of the raw products the greater the value of crop yields. For example, an increase in solids of just 1% represents *∼*20% increase in yield of certain processed products [106, 107]. High sugar content also increases the overall taste and flavor of the fresh fruit [108, 109]. For these reasons, increasing fruit solids content has been the focus of numerous tomato breeding programs. Estimates of the SS contents of the commercial cultivars of tomato range between 4.6% (mostly in fresh market) and 6.3% (mostly in processing) of the fresh weight [106]. However, accessions have been identified within related wild species of tomato, including *L. pimpinellifolium*, *L. chmielewskii* and *L. cheesmanii*, with much higher concentrations (*∼*9–15%) of SS [105, 110]. Despite the presence of this genetic variability, breeders have had limited success in increasing fruit SS or combining high SS with high yield. This has been due to various reasons, including the complex, quantitative nature of the trait [111] and the negative relationship between yield and percentage of SS [112, 113].

Fruit color is another quality characteristic in tomato that has received intensive attention. The two major groups of pigments found in tomato fruit are carotenoids and chlorophylls. However, the final color in tomato fruit is conditioned by the total amount and proportion of different carotenoids. Lycopene is the red pigment and major carotenoid in tomato. The red color is the most visible and important quality attribute of the mature tomato fruit for both fresh consumption and processing. In processing tomato, fruit color influences the grades and standards of the processed commodity. In fresh market tomato, fruit color has significant effect on its marketability. The attention to fruit color has recently been on the rise due to the increasing knowledge of the health benefits of different carotenoids. For example, fresh tomatoes and tomato products are presently major sources of lycopene, a potent natural antioxidant that is increasing in demand. Numerous epidemiological and intervention studies have demonstrated that dietary intake of lycopene-rich foods results in decreased incidence of certain cancers, including the prostate, lung, mouth, and colon cancers, and the coronary heart diseases, cataracts and may be macular degeneration [1, 5, 114–117]. This attention to lycopene is well deserved, as its antioxidant capacity is roughly twice that of *β*-carotene [118]. Unlike *β*-carotene, however, lycopene does not have any provitamin A activity. As the scientific community has become more aware of the impact of carotenoids on human health, attention has shifted to increasing tomato fruit lycopene content. Thus, an important goal of many tomato breeding programs is to develop cultivars with enhanced fruit lycopene content. In addition to lycopene, ripe tomato fruit contains *β*-carotene and small amounts of phytoene, phytofluene, *ζ*-carotene, *γ*-carotene, neorosporine, and lutein [119].

Lycopene levels of “normal” tomatoes vary with variety, and tomatoes with better red color tend to be higher in lycopene. Spontaneous mutations contributing to high fruit lycopene content have been identified within *L. esculentum*. In particular, two recessive mutant genes, *hp1* (*high pigment 1*; [120]) and *hp2* [121], were identified a few decades ago and introgressed into several tomato cultivars [122–125]. The *hp* genes increase total fruit carotenoids, including *β*-carotene [126]. However, the adverse pleiotropic effects of these genes, such as slow germination and seedling growth, seedling mortality, inferior leaf coverage, brittle stems, low yield, reduced total acidity and SS contents, high sensitivity to various pathogens and premature defoliation, have prohibited widespread commercial use of these genes [127–129]. Efforts to reduce these negative effects have largely failed and thus, currently only a handful of “lycopene rich” tomato cultivars carrying *hp1* or *hp2* are used in production. In contrast, the crimson gene (*og^c^*, *cr*), which increases fruit lycopene content at the expense of *β*-carotene [130–133], has been incorporated in many recent tomato genotypes, including breeding lines and cultivars developed at the University of Florida (http://tombreeding.ifas.ufl.edu) and North Carolina State University (http://www.ces.ncsu.edu/fletcher/staff/rgardner). Cultivars containing *og^c^* on average contain 25% more lycopene than normal cultivars. However, recently other sources of high fruit lycopene content have been identified at the Pennsylvania State University, and some processing and fresh market lines with high lycopene content have been developed by Foolad et al. (unpubl.).

Other important fruit quality characteristics of tomato include pH, titratable acidity, fruit firmness, and vitamin contents. Acidity influences the storability of processed tomato. Lower pH reduces the risk of pathogen growth in tomato products by contributing to heat inactivation of thermophilic organisms [134]. Growth of *Bacillus coagulans*, the organism that causes flat sours in tomato products, was found to be completely inhibited by a pH below 4.1 [135]. Titratable acidity has no significant effect unless pH is low. For this reason, a pH below 4.5 and citric acid of above 0.35 g/100 g of fruit fresh weight are desirable. Toward this goal, efforts have been made in different processing tomato breeding programs and some progress has been made. Although tomatoes have intermediate levels of vitamins A and C, compared with other vegetables, they rank near the top for U.S. dietary intake of vitamin A and make an important contribution to intake of vitamin C [9]. This is because tomatoes are consumed in large quantities. Plant carotenoids, in particular *β*-carotene, a major carotenoid in orange-yellow tomatoes, are the primary sources of vitamin A in tomato. The identification of genes and utilization in breeding programs for improved tomato fruit vitamin content can have significant economic as well as nutritional impacts.

Another important consideration in fruit quality improvement in tomato is in regard to flavor. Flavor is a very complex trait that is affected by numerous genetic components and nongenetic factors, not all of which are known or well understood [136–138]. Taste and smell, texture, appearance, fruit temperature, and mouth feel are among many factors that influence perception of flavor. However, a primary determinant of tomato flavor is the ratio of sugars to acids [112, 139]. High levels of SS are directly correlated with tomato-like flavor, and studies have suggested that tomato flavor can be improved by breeding for high SS and high acidity [108, 109]. Fructose and citric acid are more important to sweetness and sourness than glucose and malic acid, respectively, and pH is a better objective measure of tart taste than titratable acidity [140]. A single incomplete-dominant gene (*Fgr*) has been identified in *L. hirsutum* that increases the proportion of fructose over glucose, thus contributing to fruit sweetness [141]. Numerous aromatic volatile compounds play a major role in tomato fruit flavor, many of which are not known definitely [136–138, 142, 143]. However, from among over 400 aromatic volatiles in tomato fruit only 16 are of primary importance to flavor [144]. In addition, expression of flavor is subject to environmental variation, which hampers breeding progress [145]. Same tomato cultivars may exhibit different fruit quality characteristics under different conditions. Stage of ripeness at harvest also has significant effects on flavor [146]. Tomatoes harvested at later stages of ripeness usually are sweeter and have more “tomato-like” flavor than those harvested at “mature green” or “breaker” stage. Furthermore, environmental stresses during plant growth and fruit ripening may have positive or negative effects on fruit quality and flavor. High salinity in the growing media at certain stage of plant growth may improve tomato flavor though it may cause a reduction in fruit size [147, 148]. Flavor of fresh tomato can also be highly affected by post-harvest handling procedure and premarketing storage of the fruit [146], [149].

In summary, unlike the perception by many consumers who complain about deficiencies in the quality of modern tomatoes, fruit quality has been a major consideration in most tomato breeding programs during the past century [150]. The expectation that fresh tomatoes be harvested (usually “mature green”) and shipped thousands of kilometers during off seasons and still have a taste equivalent to a fully-mature fruit picked from the home garden may be more than the modern technology can provide [34]. In addition to varietal differences, the harvest and post-harvest procedures such as shipping and storage have significant effects on tomato quality as a whole and flavor more specifically. However, recently many tomato research programs have focused on the possibility of developing cultivars that can be harvested at later stages of maturity and yet can stand the handling necessary to transport them from the field to the market. Because stage of ripeness has so dramatic effects on fruit quality as a whole, for the past two decades significant amount of research has been devoted to better understanding of the ripening process in order to facilitate manipulation and development of cultivars with desirable fruit quality.

**Fruit ripening.**

In most field production systems around the world, fresh market tomatoes are harvested at the “mature green” or “breaker” stage. This is mainly done to prevent post-harvest damage to fruit caused by various physical and biotic or abiotic factors. Tomatoes are then allowed to ripen in storage before marketing. Such tomatoes naturally do not have the expected quality that consumers demand, certainly not the quality of home-grown vine-ripe tomatoes. In addition to the stage of ripeness, other factors that may positively or negatively affect quality attributes of fresh tomatoes include fruit firmness and shelf life. An approach to improve tomato fruit quality is to develop cultivars with extended shelf life so that tomato can be harvested at a later maturity stage [146]. However, to facilitate development of tomatoes with extended shelf life, a good understanding of the ripening process and the contributing genetic and physiological factors is necessary. During the past two decades, numerous studies have identified critical components involved in fruit ripening and softening in tomato [151–153]. The role of ethylene in initiation of ripening [154, 155] and the enzyme polygalacturonase (PG) in fruit softening [156, 157] have been well studied and characterized [152, 158]. Physiological and genetic studies have resulted in the identification and characterization of several ripening mutants such as *never ripe* (*Nr*), *nonripening* (*nor*), and *ripening inhibitor* (*rin*), genes of which are located on chromosomes 9, 10, and 5, respectively. While fruits of *Nr* mutant ripen slowly, fruits of *nor* and *rin* fail to ripen and do not exhibit any climacteric rise [158]. All three mutants show little or no activity of PG during ripening. Another ripening mutant of tomato originally found in a landrace of tomato (known as alcobaca) is *alc*, fruits of which exhibit prolonged keeping quality [159]. This mutant is controlled by a single gene (*alc*) located on the short arm of chromosome 10, about 20 cM apart from *u*, a gene conferring *uniform ripening* in tomato [160]. Traditional breeding has allowed utilization of *Nr*, *nor,* and *rin* genes and development of lines and cultivars with delayed ripening [161]. It has been determined that in most cases the use of these genes in homozygous conditions is worthless as the fruit does not ripen at all. Hybrids with ripening genes in heterozygous conditions, however, have been successful in providing for delayed ripening, longer shelf life, and increased firmness. To date, many commercial cultivars of tomato are available with these genes. Recent molecular techniques, however, have provided tools for better understanding of fruit ripening and softening in tomato and more precise mapping and cloning of related genes. Such techniques have also facilitated development of tomatoes with delayed fruit ripening, as discussed below.

**Growth habit and machine harvestability.**

Tomato plants may have different growth habits, including determinate, semi-indeterminate, and indeterminate [30, 34]. The necessity for once-over harvest resulted in the development of machinery for mechanization of processing tomatoes in the late 1960s. The first machine-harvestable cultivar was developed by GC Hanna at the University of California, Davis; [30, 162, 163] ; . Since then, processing tomato cultivars with determinate growth habit, small vine size, concentrated flowering and fruit set, slow fruit maturing and softening, and high harvest index have been developed and released for commercial use. Currently, almost 100% of the processing tomato production in the U.S. is mechanized, and almost all commercial cultivars are compact and highly determinate suitable for once-over machine harvest. Similarly, most of the fresh market tomato cultivars for field production are determinate, although with larger vine than processing types. The determinate growth habit in tomato was first reported by [164] and the gene *self-pruning* controlling it (*spsp* *=* determinate) was first characterized by [165]. The *sp* gene was originally mapped onto the short arm of tomato chromosome 6 using a classical linkage map of tomato [28, 30] and later it was mapped to the same location on the tomato molecular linkage map [166, 167]. The introduction of the *sp* allele into processing tomato cultivars transformed the industry by creating a major modification in plant architecture. However, fruits of determinate type plants in all cultivar backgrounds tend to have less sugar than congenic indeterminate types [112]. Also, fruit yield and quality of determinate plants are often inferior to those of indeterminate plants [168]. Recently the *sp* gene was fine mapped, cloned, and physically characterized [169, 170].

**Hybrid production.**

For a long time tomato breeding was mainly based on developing open-pollinated inbred cultivars and their use for commercial production. Since 1970s, however, major emphasis has been placed on production of F_1_ hybrids. Currently in many tomato producing countries, including the U.S., Japan, and Europe, tomato production is mainly based on using hybrid cultivars. The use of hybrids in tomato is not so much due to the benefits of heterosis *per se*, but to factors such as protection of breeders' research investment, combining a complex of valuable attributes such as multiple disease resistance, and production of cultivars with ripening attenuating genes in heterozygous conditions [171, 172]. However, the presence of heterosis for many important traits in tomato has been reported (see [172] for a review). Currently, in the U.S. almost all commercial cultivars of fresh market (JW Scott, University of Florida, pers. commun.) and processing tomatoes (CJ Rivara, California Tomato Research Institutue, Inc., pers. commun.; http://www.ptab.org) are hybrids.

### 1.7. Limitations of classical breeding and the need for new protocols

With the rapid increase in the size of human population, the world faces a greater demand for agricultural products than at any time in our history. Currently, the world human population is *∼*6.6 B and is expected to reach *∼*9.2 B by 2050 (http://www.census.gov/ipc/www/popclockworld.html). To prevent a major food security crisis in the world, it is estimated that food production in the developing countries will have to be doubled or tripled in the next 50 years (http://www.who.int/en). In order to achieve such levels of increase in food production, the contribution of plant breeding will have to be greater than in the past. This is due to limitations in nongenetic approaches to increase crop production, including shrinkage of natural resources (e.g., fresh water and petroleum), lack of additional arable lands, and increased restrictions in the use of chemical fertilizers and pesticides. Thus, more efficient breeding strategies are needed to assure achieving the expected increase in food production.

Traditional protocols of plant genetics and breeding, which are based on phenotypic selection (PS) and progeny testing, have been very effective in improving crop productivity and quality during the past several decades [58, 59, 173]. These methods, however, are often times consuming and not without inherent difficulties. The average length of a breeding project for a seed or vegetable crop, from hybridization and selecting the new genetic combinations to testing them in the field and introducing them in the market, is *∼*10–15 years. This lengthy process may not allow the time-sensitive need to increase crop productivity in the future. Furthermore, for many desirable agronomic and horticultural characteristics, such as disease and pest resistance, abiotic stress tolerance and improved seed/fruit quality, controlling genes may be found only within exotic genetic backgrounds such as wild species. Utilization of genetic variability within wild species often encounters various difficulties. After interspecific hybridization, a major task becomes eliminating the great bulk of undesirable genes introduced from the wild donor. A series of backcrosses to the cultivated recurrent parent alternated with concurrent inbreeding are required before the desired combinations of parental characteristics can be selected. During this process, however, some of the genes of interest from the wild donor may be lost or eliminated, limiting the level of trait expression in the progeny. In addition, wide phenotypic differences between the cultivated and wild type parents present confounding factors during evaluation and selection procedures, reducing the effectiveness of phenotypic selection. These and other problems associated with the use of traditional breeding methods warrant for the employment of techniques that have higher resolution.

An alternative approach to improving selection efficiency is to discover genetic markers that are associated, through linkage or pleiotropy, with genes or QTLs that control the trait(s) of interest. The use of markers and maps can facilitate determination of the number, chromosomal location, and individual and interactive effects of genes or QTLs affecting desirable traits [174]. Following their identification, useful genes or QTLs can be introgressed into desirable genetic backgrounds via MAS [175] or isolated via chromosome walking and map-based cloning [176]. MAS may not only speed up the process of gene transfer, but it also may allow pyramiding of desirable genes and QTLs from different genetic backgrounds. This may be an effective complementary approach to substantial crop improvement, more than what potentially is feasible through PS alone. Furthermore, in tomato, where most genetic variability can be found within the wild species, identification of genes or QTLs and their transfer into the cultivated species can be significantly facilitated by MAS [177]. In the following sections, the current status of markers and maps development, gene and QTL mapping, and MAS breeding in tomato is reviewed and discussed.

## 2. GENETIC MARKERS AND MAPS

### 2.1. Classical genetic markers

By definition, any trait that is expressed in multiple forms and inherited in a simple Mendelian fashion can be considered and used as a genetic marker. The value of genetic markers as indirect selection criteria has been known to breeders since early 1900s. Sax [178] identified an association between seed size and seed coat pigmentation in *Phaseolus vulgaris*, and breeders have used morphological markers to select for superior phenotypes for many decades. The use of morphological markers in genetics and breeding research, however, is often associated with difficulties such as expression of dominance or epistatic interactions, pleiotropic effects, and incomplete penetrance and expressivity. In tomato, there are over 1300 morphological, physiological (e.g., male sterility, fruit ripening, fruit abscission), and disease resistance genes [39], of which only less than 400 have been mapped [179–181]. The second generation of genetic markers, isozymes, became popular during 1970s and early 1980s. In tomato, 41 isozymic genes corresponding to 15 unique enzymatic reactions have been characterized, of which 36 have been mapped onto the 12 tomato chromosomes [180, 182]. Despite their great advantages, isozyme markers are very limited in number and often are not polymorphic among closely-related genotypes [183, 184].

### 2.2. Classical genetic maps

The first “classical” linkage map of tomato, showing markers on all 12 linkage groups, was reported in 1968 and included a total of 153 morphological and physiological markers [185]. For the next several years, the map was expanded and by 1975 more than 258 morphological and physiological markers were assigned to tomato chromosomes [186]. At that time, tomato had one of the best linkage maps of any plant species. The classical map information in 1970s greatly facilitated the mapping of isozyme loci, which were accomplished by the use of standard methods of segregating filial and backcross progeny as well as the trisomic technique. The first complete isozyme linkage map of tomato was published in 1980, which included 19 mapped isozyme markers, 2 approximated to two chromosomes, and 5 remaining unmapped [187]. Currently, there are 36 known isozyme markers in tomato that have been mapped to different chromosomes [180, 182]. The latest published classical linkage map of tomato consists of *∼*400 morphological, physiological, isozyme, and disease resistance genes mapped onto the 12 tomato chromosomes [30, 180, 181].

### 2.3. Contemporary molecular markers

With the advent of DNA marker technology in 1980s [188] and early 1990s, many limitations associated with morphological and isozyme markers were overcome and genetic mapping entered a new exciting and progressive era with the promise to significantly enhance efficiency of plant genetics and breeding research. A DNA marker is typically derived from a small region of DNA that shows sequence polymorphism between individuals within or between species. DNA markers, which are phenotypically neutral and literally unlimited in number, have allowed scanning of the whole genome and assigning landmarks in high density on every chromosome in many plant species, including tomato. During the past two decades, different types of molecular markers have been developed and evolved, including, but not limited to, restriction fragment length polymorphisms (RFLPs) [188], randomly amplified polymorphic DNAs (RAPDs) [189], amplified fragment length polymorphisms (AFLPs) [190], variable number of tandem repeats (VNTRs or minisatellites) [191], simple sequence repeats (SSRs or microsatellites) [192, 193], cleaved amplified polymorphic sequences (CAPS) [194], sequence characterized amplified regions (SCARs) [195], single-strand conformation polymorphisms (SSCPs) [196], expressed sequence tags (ESTs) [197], conserved ortholog sets (COS) [198], single-nucleotide polymorphisms (SNPs), and insertion deletions (InDels) [199].

Among crop species, tomato is very rich in the number of available molecular markers. Currently there are >1000 RFLP markers, most of which have been mapped onto the 12 tomato chromosomes, and *∼*214 000 ESTs (http://compbio.dfci.harvard.edu/tgi/cgi-bin/tgi/gimain.pl?gudb=tomato), of which only a small portion has been mapped onto tomato chromosomes (http://www.sgn.cornell.edu/cgi-bin/search/markers/cos_list.pl). The ESTs have been derived from over 23 cDNA libraries [8, 153] and their sequences are available on Solanaceae genome network (SGN; http://www.sgn.cornell.edu). The development and use of ESTs for various purposes in tomato are described elsewhere [8, 200, 201]; (http://ted.bti.cornell.edu). In addition to RFLPs and ESTs, several other molecular marker types, including SSRs [202–212], CAPS [207, 213, 214], RAPDs [166, 183, 215], SCARs [211], RGAs [216, 217], and AFLPs [210, 218] have been developed and mapped in tomato. At least 148 SSR markers and 77 CAPS have been mapped onto the high-density tomato genetic map [207]; (http://www.sgn.cornell.edu/cgi-bin/mapviewer/mapTop.pl?map_id=9).

Recently, the development and use of PCR-based markers have increased in tomato as these markers are generally more user friendly, cheaper, faster, and less labor intensive to develop compared with conventional DNA markers such as RFLPs and AFLPs [207, 213, 214, 219, 220]. However, a major issue in marker development in tomato is that most of the available DNA markers, including RFLPs and PCR-based markers, do not detect polymorphism within the cultivated species or between the cultivated species and closely related species such as *L. pimpinellifolium* [35, 183, 215, 221, 222]. This limited resolution restricts the use of markers in many tomato genetics and breeding programs that attempt to exploit intraspecific genetic variation or the variation within *L. pimpinellifolium*. Thus, most recently significant efforts have been devoted to the discovery of high-resolution genetic markers such as SNPs and InDels [201, 210, 223]. Such markers would allow detection of polymorphism among closely related individuals within species (e.g., between elite cultivars) or between *L. esculentum* and closely related species. For example, [223] identified one SNP per 8,500 bases when they compared two elite tomato breeding lines for 44 genes. More efforts are currently being devoted to identifying SNP markers in tomato [224–226]; (http://www.tomatomap.net). In summary, like in other plant species, the number, variety, and availability of molecular markers in tomato are continuously changing, the latest record can be found at the SGN website (http://soldb.cit.cornell.edu).

### 2.4. Contemporary molecular maps

The first molecular linkage map of tomato was published in 1986, containing 18 isozyme and 94 DNA markers (mostly cDNA clones) [227]. However, the first high density molecular linkage map of tomato, comprising of 1030 markers, was published in 1992 [228]. This map, which was constructed based on 67 F_2_ plants of an *L. esculentum* cv. VF36*-Tm2* ^a^ × *L. pennellii* LA716 cross, also displayed the chromosomal locations of 100 genes of known function or phenotype, including morphological, isozyme, and DNA markers. The marker density in this map was approximately one per 1.2 cM. A more saturated version of this map was published in 1996, reducing the intermarker space to ≤1 cM [229]. The density of markers in this map has increased over the past decade. As of March 2007, the high-density molecular linkage map of tomato consists of 2,222 mapped molecular markers, including different types of markers with an average marker distance of <1 cM (http://www.sgn.cornell.edu/cview/map.pl?map_id=9). The average estimate for the total length of the tomato linkage map is *∼*1300 cM [217].

**Figure 1:**
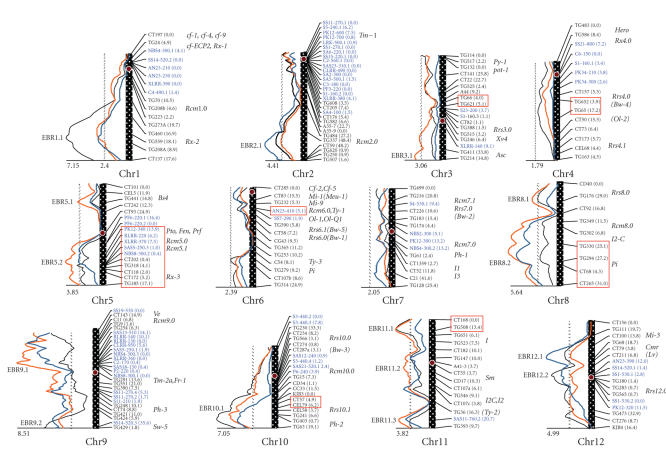
A linkage map of the 12 tomato chromosomes constructed based on a BC_1_ population of a cross between *L. esculentum* breeding line NC84173 and *L. hirsutum* accession PI126445; the framework map was adapted from [217], however, recently more markers were added to the map. The names of the markers and map distances between them are shown at the right of the chromosomes. The map includes 141 RFLP markers (black color) and 73 resistance gene analogs (RGAs; blue color). The LOD plots at the left of the chromosomes indicate the most likely positions of QTLs for early blight resistance as identified in the BC_1_ (black curves), BC_1_S_1_-1999 (red curves) and BC_1_S_1_-2000 (blue curves) populations, as adapted from [242]. The dotted black vertical lines indicates a LOD value of 2.4, a threshold value that the LOD score must cross to allow the presence of a QTL to be inferred. The highest LOD score obtained for each chromosome is shown on the *Y*-axis. Markers denoted in boxes indicate the approximate locations of QTLs detected for early blight resistance in a selective genotyping study [243]. The approximate locations of disease-resistance genes (R genes) and QTLs (Q), as inferred from published research, are shown at the right of the chromosomes. Descriptions of the R genes and QTLs are as diplayed in Table 2.

The haploid DNA content of the tomato genome is estimated to be approximately 950 Mbp (*∼*0.95 pg/1C) [45]. This means that on average 1 cM genetic map distance in tomato equals approximately 750 kb. With the high-density molecular linkage map of tomato, it is likely that any gene of interest, if segregating in this (*L. esculentum* × *L. pennellii*) population, would be within one to a few map units of at least one molecular marker. However, many agriculturally important characteristics are not segregating in this population and many of the markers in this map are not polymorphic in other mapping populations of tomato. These limitations necessitated development of genetic maps based on other inter and intraspecific populations of tomato. Thus, during the past two decades several other molecular linkage maps of tomato have been constructed, mostly based on interspecific crosses between the cultivated and different wild species of tomato (see Table 1). Most of these maps have been developed based on RFLP markers from the 1992 high-density map, although some also used other markers such as RAPDs, ESTs, AFLPs, RAPDs, and resistance gene analogs (RGAs). Most of these maps are of low-to-moderate density, having an average intermarker spacing of around 5 cM and each includes between 70 and 400 markers (see Table 1). A typical molecular linkage map of tomato is displayed in Figure 1.

For some interspecific crosses, particularly those between the cultivated tomato and the closely related wild species *L. pimpinellifolium* and *L. cheesmannii*, identification of sufficient number of polymorphic markers has been a serious limitation. For example, only about 30% of the RFLP markers in the high-density *L. esculentum* × *L. pennellii* map of tomato detected polymorphism in two different *L. esculentum* × *L. pimpinellifolium* crosses [166, 230]. Despite these limitations, to date molecular linkage maps have been developed based on interspecific crosses between *L. esculentum* and all related wild species of tomato, maybe except *L. chilense*. The latter species is only distantly related to the cultivated tomato and although it can be crossed with the cultivated species, difficult procedures such as embryo rescue or pollen mixture are needed. In addition, low fertility in the interspecific progeny may hinder development of populations suitable for genetic linkage mapping. This may demonstrate the difficulty of using this species in genetics and breeding studies, and lack of a complete linkage map based on an *L. esculentum* × *L. chilense* cross. The interspecific crosses based on which most linkage maps of tomato have been developed are those between the cultivated species and *L. pennellii* and *L. pimpinellifolium* (see Table 1).

To facilitate the use of molecular markers in tomato genetics and breeding research, some efforts have been made to develop linkage maps based on mainly PCR-based markers. One such effort resulted in the development of a map based on an F_2_ population of a cross between *L. esculentum* LA925 (E6203) and *L. pennellii* LA716 using a set of 76 SSRs and 76 CAPS [207] (see Table 1). The 152 PCR-based anchor markers covers the tomato genome at intervals of *∼*20 cM and, according to the authors, can be readily used on standard agarose gel. Accordingly, an advantage of this map is that the majority of its markers also detect polymorphism between *L. esculentum* and wild species such as *L. pimpinellifolium*, so that PCR-based markers can be used for quick genetic mapping and MAS in other interspecific populations. Furthermore, the identified markers in this map may also be useful for germplasm fingerprinting and identification, taxonomy, and studies of species relationships [207]. Recently, the number of CAPs and SSR markers in this map has been significantly increased (http://www.sgn.cornell.edu/cview/map.pl?map_id=9). Another significant effort has been conversion of RFLP markers to more friendly PCR-based markers such as CAPS [207, 213, 214].

As alluded to previously, there is limited molecular marker polymorphism within the cultivated species of tomato [35, 183, 222]. This is consistent with an earlier report of the dearth of molecular genetic diversity within the cultivated species [244]. Due to this major limitation, most of the molecular linkage maps of tomato have been constructed based on interspecific crosses, in which polymorphism is rather abundant at the level of common molecular markers such as RFLPs. Such maps, however, may have limited utility in genetic studies or breeding programs that exploit genetic variation within the cultivated species. As such, the paucity of polymorphic genetic markers has prevented detailed study of many economically important traits in the cultivated species of tomato, in particular complex traits. To overcome this problem, some efforts have been made to identify other types of molecular markers (e.g., RAPDs, SSRs, AFLPs, and SNPs) with higher resoultion to develop maps based on intraspecific populations [183, 210, 215, 245]. In particular, a great deal of effort has been made to identify SNP markers, which detect a greater number of polymorphisms between elite cultivars [68, 201, 210, 223]. The growing tomato databases of DNA sequences, in particular the tomato ESTs, is providing useful information for developing more resolving genetic markers for genome mapping, fingerprinting, trait discovery, and marker-assisted breeding within the cultivated species of tomato. It is expected that the availability of such markers will be on the rise over the next several years.

### 2.5. Comparative markers, maps, and genomes

Much of the initial comparative mapping studies in plants were done with *Solanaceae* species, including comparisons across tomato, potato (2*n* = 4*x* = 48), pepper (2*n* = 2*x* = 24), eggplant (2*n* = 2*x* = 24), tobacco (2*n* = 4*x* = 48), and petunia (2*n* = 2*x* = 14). To date, detailed genetic maps are available for tomato, potato [246, 247]; (http://potatodbase.dpw.wau.nl/uhddata.html), pepper [248], and eggplant [249–251]. Molecular maps also have been developed for petunia [252, 253] and tobacco [254]. Although the *Solanaceae* species are phenotypically diverse, their genomes are highly conserved. Comparisons across species have indicated that *Solanaceae* genomes have undergone relatively few genome rearrangements and duplications, and have very similar gene content and order.

Comparative genomics in *Solanaceae* was initiated by two studies comparing the genetic maps of tomato and potato [255] and tomato and pepper [256]. These and further studies indicated that the genomes of tomato and potato differed by only five chromosomal rearrangements, each of which involved a single break at or near a centromere resulting in paracentric inversions of the short arms of chromosomes 5, 9, 11, and 12 and of the long arm of chromosome 10 [228, 255, 257]. Such findings reinforced the high propensity (or tolerance) of plants for intrachromosomal rearrangements. The genomes of tomato and pepper, in contrast, are more extensively rearranged. There are *∼*30 chromosome breaks, including translocations, inversions (both paracentric and pericentric), disassociations or associations of genomic regions, since their divergence from a common ancestor [258, 259]. Hybridization of all examined tomato probes to positions throughout the pepper map led [258] to suggest that no major losses occurred during the divergence of the two species. The authors further reported overwhelming conservation of marker order and large orthologous linkage blocks between tomato and pepper. However, a more recent study has indicated a greater complexity in the correspondence between tomato and pepper genomes and has shown the presence of additional smaller random interruptions in synteny between the tomato and pepper [248]. The overall lengths of the tomato and pepper genetic maps are very similar [248, 258], though the DNA content of pepper is at least 2-fold greater than that of tomato [45].

A comparison of the eggplant and tomato maps revealed conservation of large tracts of collinear markers [249], similar to that observed in potato and pepper. Accordingly, eggplant and tomato were differentiated by 28 rearrangements, including 23 paracentric inversions and five translocations, during their evolution from the species' last common ancestor. The eggplant nuclear genome is slightly larger than that of tomato and contains 1100 Mb of DNA (1.2 pg/1C) [45]. As judged based on genome comparisons across tomato, potato, pepper, and eggplant, it seems that the primary mechanism for chromosome evolution in *Solanaceae* has been paracentric inversion [249]. Furthermore, a recent comparative genome (sequence) analysis of seven *Solanaceae* species, including tomato, potato, pepper, eggplant, petunia, tobacco, and *Nicotiana benthamiana*, confirmed a high degree of sequence conservation [260]. The same study, however, also identified some species-specific sequences suggesting divergence within *Solanaceae* genomes.

A few studies have compared tomato genome with other plant species, including *Arabidopsis* [8, 198, 261, 262] and coffee [263]. Seemingly, there is conservation of gene content and order between tomato and *Arabidopsis* since their divergence from a common ancestor *∼*112 million years ago. A comparison of over 27 000 unigenes (unique consensus sequences) revealed that 70% of the unigenes have identifiable homologs in the *Arabidopsis* genome [8]. Furthermore, of the 10 largest conserved multigene families, a majority shares similar copy number in tomato and *Arabidopsis* suggesting that multiplicity of these families may have occurred before their divergence. An exception was observed for the E8-likr protein family, which is associated with fruit ripening and has higher copy number in tomato than *Arabidopsis*. Moreover, genes related to metabolism have remained most conserved whereas those encoding transcription factors are among the fastest evolving. When comparing gene repertoires of tomato and coffee, it appeared that tomato had a perfect gene-for-gene match with coffee [263]. This was not surprising as the two species have similar genome size and chromosome karyotype (coffee *n* = 11) and architecture. Although from different families (coffee family *Rubiaceae*), both coffee and tomato belong to the Asterid I clade of dicot families. Further information on comparative genomics of tomato can be found elsewhere [6, 264–267].

## 3. MAPPING GENES AND QTL_s_

Tagging and mapping of single-gene traits in tomato, including many morphological, physiological, and disease resistance traits, started in 1930s [268], much earlier than in many other crop species. Tagging of single-gene traits with molecular/biochemical markers started in 1970s. [110] reported an association of root-knot nematode (*Meloidogyne incognita*) resistance with a rare form of isozyme acid phosphatase locus, *Aps-1* ^1^. Later on this association was determined to be due to a tight linkage between the gene controlling nematode resistance in tomato, *Mi* [269], and the *Aps* locus on chromosome 6 [270]. Subsequently, linkages were reported between isozyme markers and genes controlling a few other important traits in tomato, including male-sterility [271] and self incompatibility [272]. Since then, tagging of many other simply inherited traits with molecular markers has been reported and currently linked markers are available for many agriculturally and biologically important traits in tomato.

The use of genetic markers to identify QTLs controlling complex traits in tomato started in the 1980s. Earlier studies mainly used morphological and isozyme markers and filial (e.g., F_2_) or backcross (e.g., BC_1_) populations to identify QTLs for different quantitative traits, including leaf ratio, stigma exsertion, fruit weight, seed weight, internode length, number of nodes, number of flowers, stem width, plant size, plant height, and cold tolerance [273–276]. However, the first comprehensive and systematic analysis of the use of molecular markers to dissect genetic controls of complex traits and to identify underlying QTLs was that of [167]. In this study, a rather complete RFLP linkage map of tomato was used to identify and map QTLs for fruit quality characteristics, including fruit size, pH, and soluble solids content. This study demonstrated for the first time that quantitative traits could be resolved into discrete Mendelian factors. Subsequently, QTL mapping became very popular in tomato genetics and breeding research, where QTLs have been identified for numerous agriculturally and biologically important complex traits. Practically, it is difficult to provide a complete account of all genes and QTLs that have been identified and/or mapped in tomato chromosomes. Rather in this article a tabulated summary of most genes and QTLs which have been identified and mapped in tomato chromosomes during the past two decades is presented (see Tables 2, 3, 4, and 5). Furthermore, a summary discussion of the populations used for mapping as well as mapped genes and QTLs for certain important traits in tomato is provided below.

### 3.1. Populations used for mapping

As alluded to earlier, because of limited DNA polymorphism within the cultivated species of tomato, most mapping populations have been based on interspecific crosses between the cultivated tomato and related wild species. In fact, almost all wild species of tomato have been used for gene and/or QTL mapping, although with different frequencies. For example, while *L. pennellii*, *L. pimpinellifolium, * and *L. hirsutum* have been used extensively, wild species *L. chilense* and *L. peruvianum* have been used infrequently and mainly for mapping of a few major disease resistance genes (see Tables 2, 3, 4, and 5). Reasons for this discrepancy include difficulties normally encountered when using *L. chilense* or *L. peruvianum* to develop mapping populations. For example, in addition to problems in making original crosses and developing F_1_ hybrids, low fertility and presence of excessive undesirable variation in early filial or backcross populations exacerbate the difficulties. Although *L. pennellii* is also distantly related to the cultivated tomato, the presence of a self-compatible accession (LA716), which was originally used to develop the first molecular linkage map and the high-density molecular map of tomato (see Table 1), facilitated frequent use of *L. esculentum* × *L. pennellii*-derived populations for gene mapping and QTL identification. On the other hand, crosses with *L. pimpinellifolium* have been used frequently for mapping experiments mainly because of its close phylogenetic relationship with the cultivated tomato, the ease of crosses and handling of segregating populations and its red-fruited characteristic [230].

As to the types of populations, early filial (e.g., F_2_ and F_3_) and backcross populations (e.g., BC_1_ and BC_2_) have been used more often than advanced populations for genetic mapping (see Tables 2, 3, 4, and 5). While early segregating populations have the advantages of easiness of development and presence of high linkage disequilibrium, they often have several disadvantages including: (1) limitations in trait evaluation (e.g., in F_2_ and BC_1_ evaluation is based on individual plant performance that may not be repeatable), (2) detection of loose marker-QTL association due to high levels of linkage disequilibrium, (3) presence of excessive genetic variation when using wide crosses (which may negatively affect the accuracy of detecting and mapping of genes and QTLs), (4) instability due to changes in their genetic constitutions from generation to generation, and (5) not immediately applicable for breeding purposes (see below). To avoid such problems, some more stable segregating populations such as recombinant inbred lines (RILs), advanced backcross populations (AB; e.g., BC_2_ and BC_3_), backcross inbred lines (BILs, a.k.a. inbred backcross lines or IBC, e.g., BC_2_S_3_, BC_3_S_2_), and introgression lines (ILs) have been developed and used for gene and QTL mapping in tomato, as described below.

The use of RILs in genetic mapping has several advantages, including the possibility of having multiple replications for trait evaluation, repetition of experiments in different years and locations and by different researchers, evaluation of the population for multiple traits in different environments, and detection of mainly tightly linked QTLs (due to low linkage disequilibrium). In tomato, currently there are a few RI populations available, including one based on an *L. esculentum* × *L. cheesmanii* cross [233] and three based on different *L. esculentum* × *L. pimpinellifolium* crosses [277, 278]; Foolad et al. (unpubl.). Although RI populations are valuable resources for genetics and mapping studies, there are several disadvantages in using them, including the long time it takes to develop them, potential difficulties in developing RILs when using interspecific crosses (e.g., sterility or self-incompatibility problems), large genetic diversity among RI lines and presence of large genomic contributions from the wild species (on average 50%), which may cause difficulties with evaluation of certain traits. In tomato, RI populations have been used for mapping QTLs for various characteristics, including fruit weight and SS [279], several morphological traits [280], abiotic stress tolerance [281], seed weight [279] and disease resistance and fruit quality [282]; Foolad et al. (unpubl.).

In comparison to RILs, AB populations and BILs may be more desirable for QTL mapping in self-pollinated crops, in particular when using interspecific crosses [283–285]. Such populations have much smaller genome contributions from the wild donor parent compared to RILs, providing more uniform genetic backgrounds for trait evaluation. These populations are particularly useful for studying the effect of exotic alleles on the agronomic performance of elite cultivated lines [285]. Furthermore, high levels of homozygosity in these populations would allow family/line evaluations over locations and years, an advantage similar to that for RI populations. If properly developed (i.e., having a good coverage of the donor parent in the background of the cultivated recurrent parent), BILs can provide accurate identification and characterization of genes or QTLs. Moreover, such populations can simultaneously be used as breeding materials for crop improvement, a great advantage over other types of mapping populations [283]. During the past several years, BILs have been used frequently for mapping QTLs for many traits in tomato (see Tables 2, 3, 4, and 5), including fruit quality [68, 231, 240, 283, 286] and disease resistance [242, 287–289]. In general, higher levels of homozygosity in RI and BI populations, as compared to early segregating populations, allow more precise estimation of the locations and effects of QTLs.

Another population type that has been developed and extensively used for gene and QTL mapping in tomato is introgression lines (ILs). In comparison, whereas each BIL may contain several chromosomal segments from the wild donor parent in an otherwise *L. esculentum* genetic background, each IL technically contains only a single introgression from a wild species. In other words, ILs are near isogenics to the original *L. esculentum* recurrent parent. Such permanent mapping populations, which are considered as genetic libraries, are a powerful tool for various studies, including placing new markers on tomato chromosomes, identification of region-specific DNA markers, and discovery and characterization of genes or QTLs underlying important qualitative and quantitative characteristics [290]. The first developed IL population of tomato consisted of 50 lines, each containing a single introgression from *L. pennellii* LA716 in the background of processing tomato cultivar M-82 [238, 291, 292]. Since 1995, however, the number of ILs in this population has increased to 76, which totally represent the entire genome of *L. pennellii* LA716 in homozygous or heterozygous conditions. This IL population delimits 107 marker-defined mapping bins, each bin having an average length of 12 cM [293–295]. In addition to this IL population, a total of 99 NILs and BILs derived from a cross between processing tomato cultivar E6203 and a single plant of *L. hirsutum* accession LA1777 have been developed [236]. In this population, most of the lines contain a single-defined introgression from the *L. hirsutum* parent in the *L. esculentum* genetic background, and together the lines provide a coverage of more than 85% of the genome of the LA1777 plant used as the donor parent (note that LA1777 is not an inbred accession and thus the plant used for developing this population does not represent the total variation within LA1777). More recently, an IL population containing introgressions from *S. lycopersicoides* in the backgroun of *L. esculentum* has been developed [296].

The permanent mapping populations have been used for identification and mapping of genes and QTLs for many important tomato traits (see Tables 2 and 4; http://zamir.sgn.cornell.edu), including fruit weight, shape, SS content, pH and yield [236, 238, 290, 297, 298], carotenoid content in relation to fruit color [294, 299], antioxidants [300], and disease resistance [282]. The mapping power of ILs is generally greater than traditional QTL mapping populations and as a result often larger number of QTLs is detected by such populations [238]. Furthermore, although ILs are mainly used for low-resolution mapping, they also can be used for high-resolution mapping by developing F_2_ populations of crosses between targeted ILs and the recurrent parent. Using this strategy, ILs have been used to develop NILs for fine-mapping and map-based cloning of several genes and QTLs controlling various traits, including *self pruning* [170], color mutants [299, 301], fruit soluble solids content [98, 302–304], fruit weight [305], fruit shape [304, 306], stigma exsertion [307], and a few other traits as shown in Table 7. Moreover, the IL populations can be used for MAS pyramiding of important QTLs, as it has been done in case of tomato yield and soluble solids QTLs [308].

The use of BILs and ILs also allows development of NILs for particular genes, QTLs or segments of a chromosome, which can be used for further analyses such as validation of individual effects of QTLs in uniform *L. esculentum* background, marker-assisted transferring of individual or combination of QTLs to different genetic backgrounds, determination of the presence and nature of association (linkage or pleiotropy) between different traits, determination of QTL × QTL, QTL × genetic background and QTL × environment interactions, and fine-mapping and possible cloning and characterization of underlying genes or QTLs. To date, NILs have been developed for QTLs controlling many important traits in tomato, including various disease resistance and fruit quality characters (see Tables 2, 4, and 7).

### 3.2. Disease resistance genes and QTLs

In tomato, mapping disease resistance genes and QTLs has been the focus of many mapping activities. Identification of genetic markers associated with disease resistance in tomato started in 1970s with the pioneering work of Charles M. Rick and his co-workers who identified an association between root-knot nematode resistance and a form of isozyme acid phosphatase, *Aps-1* ^*1*^ [372]. At the time, resistance to root-knot nematodes was known to be genetically inherited and controlled by a single dominant gene, known as *Mi*, located on the long arm of chromosome 6 [373]. Since 1970s, however, genetic markers, in particular DNA markers, have been used extensively to tag or map major genes for vertical (a.k.a. race-specific) resistance and QTLs for horizontal (a.k.a. field or race-nonspecific) resistance to many fungal, bacterial, viral, and nematode diseases in tomato. In Table 2, all known mapped disease resistance genes and QTLs in tomato together with information on gene/QTL symbols, the causal agents of the diseases (pathogens), genetic source(s) of the resistance, chromosomal locations of the resistance genes/QTLs and the cited references are displayed. The space limitation in this review article does not allow for discussion of procedures or methodologies used for mapping of genes or QTLs for resistance to different diseases. However, not withstanding that the procedures and methodologies used for different diseases vary, some general comments can be made as to the mapping of resistance genes and QTLs as follows.

1. With some exceptions (e.g., [64–69, 374, 375]), most genes and QTLs for disease resistance have been identified in the related wild species of tomato and mapped using interspecific segregating populations (see Table 2).
2. For some diseases, often multiple gene resources have been employed to identify and map resistance genes and QTLs. The identification of multiple resistance genes/QTLs for each disease may provide the opportunity to pyramid resistance in selected lines and cultivars using a MAS approach.
3. For most field (horizontal) resistance traits, often multiple QTLs have been identified in each study. In many cases, it has been difficult to determine the precise location or actual effect or importance of each QTL in the original studies. Many studies have suggested development of NILs and sub-NILs to obtain such necessary information.
4. In most cases, early filial and backcross populations, such as F_2_ and BC_1_, have been used for mapping. More recently, however, advanced segregating populations such as RILs, BILs, and ILs have been utilized (see Table 2). Such populations have provided better mapping resolution.

Knowledge of the linkage between molecular markers and resistance genes or QTLs can facilitate an effective, and in some cases rapid, transfer of resistance to various tomato genetic backgrounds through MAS. As described below, MAS has been employed for disease resistance breeding in tomato, in particular by many seed companies. In fact, the utility of MAS for disease resistance breeding has superseded its utility for any other trait in tomato breeding.

### 3.3. Insect resistance genes and QTLs

There are fewer reports of genes or QTLs identified for insect resistance in tomato than those for disease resistance. It is generally very challenging to set up controlled experiments on insect resistance to identify underlying genetic factors. However, over the years some research has been conducted toward this goal. Much of the insect resistance mapping experiments in tomato have been conducted using *L. pennellii* accession LA716 as a resistance parent. The multiple pest resistance of this accession is mediated by acylsugars exuded by type-IV glandular trichomes on the leaf surface of these plants [73]. The acylsugars act as feeding deterrents for tomato pests, including potato aphid, green peach aphid, tomato fruitworm, and beet armyworm, as feeding and oviposition deterrents for the leafminer and silverleaf whitefly. In one study, an F_2_ population of an *L. esculentum* × *L. pennellii* (LA716) was surveyed for acylsugar accumulation, and a total of five QTLs were detected on *L. pennellii* chromosomes 2, 3, 4, and 11 with association with one or more aspects of acylsugar production [73]. In a follow-up study, attempts were made to transfer these QTLs via MAS to an *L. esculentum* genetic background [376]. However, resulting BC_3_ progeny plants with complementary subsets of 3–5 QTLs were found to accumulate only low levels of acylsugars, prompting the authors to speculate presence of other yet unidentified genetic factors controlling acylsugar accumulation. Furthermore, the derived lines exhibited high levels of linkage drag. This research clearly demonstrated the complexity of the trait and difficulties in developing tomato plants with improved insect resistance. In another study, an F_2_ population of an intraspecific cross between two *L. pennellii* accessions (LA716 and LA1912) was employed to study the genetic basis of acylsugar fatty acid composition [377]. A total of six QTLs were detected which together could explain 23–60% of the variation for each of nine fatty acid constituents. These QTLs were different from those which had been detected in an *L. esculentum* × *L. pennellii* F_2_ population [73], further suggesting complexity of the genetic control of acylsugar production [377].

Based on most research reports, specific insect resistance genes often confer resistance to only one insect species or to a closely related species within the same genus. However, the *Mi* gene, which originally was identified as a dominant gene for resistance to a root-knot nematode [269] is an interesting exception. After the *Mi* gene had been cloned [312, 388] it was determined that it was the same locus as *Meu1*, which confers resistance to the potato aphid, *Macrosiphum euphorbiae* [38]. Currently, *Meu1* (*Mi*) is the only insect resistance gene that has been cloned from a plant species. This gene is a member of leucine zipper, nucleotice binding, leucine-rich repeat family of plant resistance genes [389], many members of which have been found to confer isolate-specific resistance to viruses, bacteria, fungi, and nematodes [390]. However, the *Mi* gene is the first example of a plant resistance gene active against two such distantly related organisms belonging to different phyla. A later study revealed that several isolates of potato aphid and green peach aphid (*Myzus persicae*) can overcome the resistance mediated by *Mi* (*Meu1*), limiting the use of this gene for aphid control in tomato [391]. In a more recent study, two tomato BI populations, derived from crosses between two different aphid susceptible *L. esculentum* lines and two aphid resistant accessions of *L. pennellii* and *L. hirsutum*, were evaluated for resistance to both potato aphid and green peach aphid [80]. Field screening over two years resulted in the identification of seven BILs (BC_2_S_3_ to BC_2_S_6_) with resistance to both types of aphid. These BILs can be useful for breeding tomatoes for aphid resistance using PS and/or MAS.

In conclusion, unlike in the case of disease resistance, there has been rather limited research progress in identification, mapping or transfer of genes/QTLs for insect resistance in tomato. Although there are several reasons for these shortcomings, difficulties in phenotypic screening for insect resistance, problems with linkage drag, and the ease of insect control by pesticides are probably the main ones. However, with the increasing restrictions on the use of pesticides and the advancements in marker development, it is expected that more research will be devoted to the identification and use of makers for insect resistance breeding in tomato.

### 3.4. Abiotic stress resistance genes and QTLs

In most crop species, traditional breeding protocols for improved abiotic stress resistance/tolerance has been generally unrewarding mainly due to the very complex nature of such traits. Thus, identification of genetic markers that are associated with tolerance traits and their use in marker-assisted breeding is regarded a promising approach. The challenge is to improve the efficiency of selection for stress tolerance by integrating marker technology with the conventional protocols of plant genetics and breeding [81]. In tomato, while significant efforts have been devoted to the identification and mapping of QTLs conferring tolerance to environmental stresses such as salinity, drought and low temperatures, less mapping research has been conducted on other streses, including high temperatures (for a review see [81]). It should be noted, however, that some heat-tolerant inbred lines and commercial cultivars of tomato have been successfully developed using traditional breeding protocols [33, 90, 392]. In fact, it seems that in tomato more progress has been made in breeding for heat tolerance than breeding for tolerance to any other environmental stresses. This could be due to a greater emphasis that has been placed on breeding for heat tolerance. Below, the recent mapping activities on different abiotic stresses in tomato are briefly reviewed and discussed.

**Salt tolerance.**

More mapping research has been conducted on tomato salt tolerance (ST) than tolerance to any other environmental stresses [81]. Also, because ST is a developmentally regulated, stage-specific phenomenon, efforts have been made to identify contributing genetic components at specific developmental stages. For example, QTLs have been identified for ST during seed germination, vegetative growth and later stages in tomato (see Table 3). The identified QTLs for tolerance at different stages can potentially be transferred to desirable genetic backgrounds through a pyramiding approach using MAS to develop tomatoes with improved ST throughout the plant ontogeny.

More efforts have been made to identify QTLs for ST during seed germination than any other stage. For example, QTLs have been identified in different tomato wild species and under different levels of salt stress. Comparisons of the QTLs identified for ST in different interspecific populations of tomato, including those derived from *L. esculentum* × *L. pennellii* and *L. esculentum* × *L. pimpinellifolium* crosses, indicated that some QTLs were conserved across species whereas others were species-specific [378, 382, 383]. Comparisons of the QTLs identified in different populations of the same cross indicated stability of QTLs across populations and generations. Furthermore, it has been determined that often same QTLs contribute to tolerance at different levels of salt stress [393].

ST during vegetative growth in tomato is more important and more complex than ST during seed germination, and numerous physiological components may affect tolerance at this stage (see [281] for a detailed review). Although a good progress has been made in mapping QTLs for ST during the vegetative stage in tomato (see Table 3 for references), more research is needed for a better understanding of the underlying genetic components. The overall results of the mapping studies support a previous suggestion [394, 395] that ST during the vegetative stage in tomato is controlled by more than one gene and is highly influenced by environmental variation. However, most studies indicate the presence of some major QTLs, suggesting the potential utility of MAS for improving tomato ST during the vegetative stage.

In comparison to the research conducted during seed germination and the vegetative stage, limited research has been conducted to identify QTLs for ST during reproduction in tomato [387, 396–398], and the reported QTLs have not been verified in independent studies or populations. A few QTL mapping studies also have examined relationships among ST at different developmental stages [93, 399]. The overall results support the suggestion that different genetic and physiological mechanisms contribute to ST during different stages of plant development. In theory, simultaneous improvement of ST at different plant stages should be possible through the use of marker-assisted breeding and pyramiding of various tolerance components. In practice, however, for improving tomato ST via MAS, a good knowledge of carefully identified and verified QTLs at all stages of plant development is required.

**Cold tolerance.**

The physiology and genetics of tomato cold tolerance (CT) has been investigated at different developmental stages (see [81] for a review). However, compared to that for ST, much less research has been conducted to identify markers that are associated with genes/QTLs contributing to CT at different developmental stages in tomato. For CT during seed germination, the only published research is that of [378] in which a few QTLs were identified using backcross progeny of an interspecific cross between *L. esculentum* and *L. pimpinellifolium*. More recently two additional studies were conducted to identify QTLs for CT during seed germination in tomato. While in one study a selective genotyping approach was used in a large *L. esculentum* × *L. pimpinellifolium* BC_1_ population ( *N* = 1000), the second study used an F_9_ RIL population ( *N* = 145) of the same cross Foolad et al. (unpubl.). These two studies verified all of the QTLs that were identified in the original study [378] and further detected a few new QTLs. The combined results of these studies suggest that CT during seed germination in tomato is a quantitative character controlled by more than one gene. A comparison of QTLs in different populations of the same cross indicates that most QTLs are stable across populations and generations, whereas a few are population specific.

QTL mapping studies for CT during vegetative growth and reproduction are scarce. This is unfortunate, as the value of QTLs for tolerance at these stages would be much greater than that for CT during seed germination. This is because most field tomato productions are based on the use of transplants that are often produced in warm greenhouses. Tomatoes with CT during seedling stage, in contrary, can facilitate early field planting, which may lead to early harvest and huge economic incentives. Similarly, tomato production in temperate climates with frequenct cold spells during the season can be more successful by the use of cold-tolerant cultivars. To the author's knowledge, there are only two reports of QTLs for CT during the vegetative stage in tomato. In one study, using BC_1_ population of an interspecific cross between a cold-sensitive *L. esculentum* line and a cold-tolerant *L. hirsutum* accession, [275] identified three QTLs responsible for growth at low temperatures. In another study, several QTLs were identified associated with shoot wilting and root ammonium uptake under chilling temperatures in a *L. esculentum* × *L. hirsutum* BC_1_ population [379]. However, extensive research is needed to determine the actual value of these QTLs from *L. hirsutum* and to identify and validate other potentially useful QTLs for CT breeding in tomato. Because of the natural complexity of CT characteristics, molecular marker technology is expected to be useful in identifying critical genetic components leading to development of cold-tolerant tomatoes.

**Drought tolerance.**

Comparatively, less mapping research has been conducted on tomato drought tolerance (DT) than tomato ST or CT. There may be only one published report on QTLs for DT during seed germination [86], in which four QTLs were identified in backcross progeny of an *L. esculentum* × *L. pimpinellifolium* cross. In a more recent study, F_9_ RILs of the same cross were evaluated for germination rate under drought stress and a composite interval mapping detected several QTLs for DT on different tomato chromosomes Foolad et al. (unpubl.), consistent with results of the original study. The combined results indicated presence of stable QTLs for DT during seed germination across populations of the same cross, suggesting the usefulness of these QTLs for improving tomato DT during seed germination by MAS. The stability of these QTLs across other populations and interspecific crosses, however, should be examined before considering them for MAS transfer to the cultivated tomato.

Similar to the situations with tomato ST and CT, limited research has been undertaken to characterize the genetic control of, or to develop tomatoes with, improved DT during the vegetative or reproductive stage. In one study, to facilitate selection for low ffl (^13^C/^12^C discrimination), 3 QTLs associated with this trait were identified using progeny of a cross between *L. esculentum* and *L. pennellii* [380]. However, subsequently it has not been determined whether selection for these QTLs would increase water use efficiency or DT in tomato. There is no published research on QTLs for DT during the reproductive stage. Yet again, if we expect using MAS technology for improving DT in tomato, the first and most important step is to identify reliable QTLs for DT-related characteristics during important growth stages. To the author's knowledge, unfortunately, no such effort is currently underway.

**Relationship among tolerances to different stresses.**

Although several studies have investigated physiological and genetic relationships among tolerances to different abiotic stresses in tomato, only a few studies have used QTL mapping as a tool for such investigations and which focused only on the seed germination stage [94, 400–403]. The overall results of these studies have indicated the presence of genetic relationships among cold, salt, and DT during seed germination. For example, a few QTLs were identified with effects on seed germination under two or three stresses; such QTLs were referred to as stress-nonspecific QTLs. Comparatively, a few other QTLs were identified with effects on germination rate only under specific stress conditions, referred to as stress-specific QTLs. In summary, the results suggest that some genes affect tomato seed germination under different stress conditions while other genes are more specific. Further research is necessary to identify and compare genes/QTLs for tolerance to different stresses at different developmental stages. Such information will not only be scientifically intriguing, but also may be useful for developing plants with tolerance to diffeent environmental stresses.

### 3.5. Genes and QTLs for flower- and fruit-related characteristics

Molecular markers have been used to map genes or QTLs for many flower- and fruit-related characteristics in tomato, including exerted stigma, petal and sepal characters, fruit size, shape, color, soluble solids content, pH, lycopene, acidity, flavor, ripening, and many others. Table 4 summarizes the genes and QTLs that have been identified and/or mapped on tomato chromosomes for such characteristics during the past 2-3 decades. It can be seen from the table that often several groups have conducted research on the same or similar characteristics, or the same traits have been studied using different interspecific populations of tomato. The status of marker development for some major fruit characteristics in tomato is briefly discussed.

**Fruit size.**

This trait has been studied very extensively, as can be seen from Table 4. Although there is variation in fruit size within the cultivated tomato, differences are much greater when comparing wild species (with fruit size as small as 1 g with 2 locules) with the cultivated species (with fruit size as large as 1000 g with 10 or more locules). Thus, most QTL mapping experiments have been based on the use of interspecific populations. Traditional breeding studies had suggested that the genetic control of this trait was not very complex, as the trait could be easily manipulated through PS and breeding due to its high heritability. Molecular mapping studies have revealed presence of about a couple dozens of QTLs for fruit size in tomato, which have been mapped to all 12 chromosomes (see Table 4), some of which with very large effects [414]. Many studies have identified QTLs in the same chromosomal locations, and the most recent studies have not identified novel QTLs for tomato fruit size that were not previously reported. For example, in an F_2_ population of a cross between an *L. pimpinellifolium* accession (LA1589), with average fruit weight of 1 g, and the *L. esculentum* cultivar Giant Heirloom, with fruit size as large as 1000 g, no novel QTLs were identified [40]. This study detected all major QTLs for fruit size that were previously identified in other studies, including *fw1.1* (explaining *∼*17% of the variation), *fw1.2* (*∼*13%), *fw2.1* (*∼*12%; previously known as *locule number, lc*), *fw2.2* (*∼*23%), *fw3.1* (*∼*12%) and *fw11.3* (*∼*37%; previously known as *fasciated, f*). Of these QTLs, *fw2.1* (*lc*) and *fw11.3* (*f*) are associated with an increase in locule number. It has been suggested that exceptionally large-fruited fresh market tomato varieties, such as Giant Heirloom, were evolved as a result of novel combinations of all these major QTLs, whereas medium-size processing tomatoes (with 2–4 locules) were evolved from QTLs *fw1.1*, *fw2.1*, *fw2.2*, *fw3.1*, *fw3.2,* and *fw11.3*, none of which affecting locule number [40].

One of the major QTLs, *fw2.2*, which was detected in many QTL studies in tomato (see Table 4), has been cloned and characterized [305]. This QTL was reported to make the largest contribution to the difference in fruit size between most cultivated tomato genotypes and their small-fruited wild species counterparts [435]. In fact, many studies that used interspecific tomato populations for mapping identified *fw2.2*. As to other fruit size QTLs, a comparison of their map positions across studies indicates colocalization of their positions (see [414]), supporting the hypothesis that the majority of fruit size variation in the cultivated tomato is attributed to allelic variation at a rather limited number of loci [427]. Many studies also have indicated colocalization of QTLs for fruit size and solids contents (see [414]), confirming the negative correlation between these two traits in tomato reported in many studies (e.g., [112]).

**Fruit shape.**

There are extensive variations in fruit shape in the cultivated tomato, including oblate, globe (round), ovate (blocky, square round), heart shaped, ellipsoid (plum-shaped), elongated (cylindrical, long oblong) and pear shaped (pyriform). Traditional genetic studies had identified several genes controlling fruit shape in tomato such as *pr* (pyriform), *o* (ovate), *bk* (beaked tomato), *n* (nipple-tip tomato), *f* (fasciated), and *lc* (for locule number) [436]. During the past two decades, a few of these genes and several other genes and QTLs affecting fruit shape in tomato have been located on tomato molecular linkage map and/or cloned and characterized at the molecular level (see Tables 4 and 7). For example, a major fruit-shape QTL (termed *fs8.1*) differentiating fresh market (round) and processing (blocky) tomatoes was mapped on tomato chromosome 8 [437] and later cloned and characterized [438]. *fs8.1* exerts its effect by changing the length of carpels during preanthesis resulting in longer and larger mature fruit. Similarly, another major fruit-shape QTL termed *ovate*, controlling the transition from round to pear-shaped fruit, was mapped [428] and cloned and characterized [439]. The overall results from different studies indicate that most of the variation in the cultivated tomato fruit shape is controlled by a few major loci and that the observed variation is most likely due to allelic variation at these loci [427].

**Fruit color.**

Because it is an important fruit quality characteristic in tomato, color has been the focus of numerous mapping studies. The attention to tomato fruit color has recently increased as the health benefits of lycopene, the major carotenoid in tomato that is responsible for the red fruit color, has become more obvious [114, 440–443]. As indicated earlier, several major genes with significant contribution to high contents of fruit lycopene (e.g., *hp-1*, *hp-2*, *dg* and *Og^c^*) and other carotenoids (e.g., beta-carotene, *B*) were previously identified and mapped onto the classical linkage map of tomato [30, 444]. However, during the past two decades, numerous QTLs and candidate genes with significant effects on fruit color and/or lycopene content were identified and mapped onto tomato chromosomes along with the previously identified genes (see Table 4). While some of the identified QTLs mapped to the chromosomal locations of many of the known genes in the carotenoid biosynthesis pathway, many mapped to unknown locations (e.g., see [294]). Therefore, it was suggested that there might be more genes affecting fruit color in tomato than those known to affect based on the carotenoid biosynthesis pathway [294]. Currently, a few research programs in the U.S. and around the world are conducting research to identify, map, and possibly clone new genes involved in determining fruit color in tomato. In addition, there are numerous programs attempting to improve tomato nutritional quality either through traditional breeding or transgenic approaches [299, 409, 445].

**Fruit soluble solids.**

As indicated earlier, fruit soluble solids content (SSC) has been the focus of numerous tomato genetics and breeding programs worldwide. However, due to a negative correlation between yield and SSC in tomato, breeders have had limited success in increasing SSC of high-yielding tomato cultivars using traditional phenotypic selection [113]. Although fruit size and yield have been increased substantially via traditional breeding, SSC has remained essentially unchanged. To facilitate alternative approaches to increasing SSC of high-yielding tomato cultivars, significant efforts have been devoted to identify QTLs for high SSC. The hope has been to identify SSC QTLs that may not have any adverse effect on fruit size. Currenlty, there are more than 20 published studies that have identified QTLs for high fruit SSC in tomato (see Table 4). Although these studies used different interspecific populations, there have been significant overlaps in the locations of the QTLs identified across studies (e.g., see [414]). Furthermore, many studies have revealed that QTLs that positively influence SSC are mostly at the same chromosomal locations as QTLs that negatively impact fruit weight [414]. Although a few studies have reported SSC QTLs with no apparent effect on fruit size (e.g., [98]), there is no verfication of the effects of such QTLs via MAS experiments.

**Fruit yield.**

Yield is a complex trait that is directly or indirectly affected by numerous genetic and nongenetic factors. For this reason, the heritability for yield is often very low in most crop species, including tomato. Technically it is difficult to identify QTLs that may be truly indicative of genetic yield potential and could be utilized in marker-assisted breeding. Nevertheless, many studies have conducted QTL mapping for fruit yield in tomato and identified QTLs for traits such as total yield, red yield, and green yield (see Table 4). Basically, QTLs for yield have been identified in different interspecific populations of tomato and mapped to all 12 chromosomes. However, unlike QTLs for fruit weight and SSC that were rather consistent across studies, there is limited concordance across studies in regard to yield QTLs (see Table 4). This is not surprising considering the very complex nature of the trait and its low heritability. Furthermore, there is no published report of the use of fruit yield QTLs for MAS in tomato, and it is not expected that at least in the near future such QTLs would have wide utility for improving tomato fruit yield.

**Fruit ripening.**

As indicated earlier, traditional genetics and breeding research had resulted in the identification and manipulation of several ripening-related genes. During the past two decades, molecular biology techniques facilitated characterization of such genes and identification and mapping of many other ripening-related genes, loci, and QTLs (see Table 4). Furthermore, a few ripening-related genes have been genetically characterized, fine mapped and cloned using map-based cloning techniques (see Table 7, [155, 424]). More detailed lists of genes with effects on fruit ripening in tomato can be found in [151, 153, 446]. Among the major ripening genes, at least one (*rin*) has been used in marker-assisted breeding (see Table 6).

**Other traits.**

In addition to the traits described above, genes or QTLs have been identified for numerous other flower- and fruit-related characteristics as well as traits such as self incompatibility, unilateral incongruity, transgressive segregation, self-pruning (determinate type plants), jointless pedicel, and seed size and number among others, as shown in Tables 4 and 5. The available mapping information may be useful for basic research such as identifying and cloning genes underlying these traits as well as for breeding purposes. The limited space here does not allow discussion of these traits.

## 4. MARKER-ASSISTED SELECTION

MAS can be defined as selection for a trait based on the genotype of an associated marker rather than the trait itself. In essence, the associated marker is used as an indirect selection criterion. The potential of MAS as a tool for crop improvement has been extensively explored [448–450]. In theory, MAS can reduce the cost and increase the precision and efficiency of selection and breeding. It may offer unique opportunities to circumvent many potential problems associated with PS, and thus may be more useful. MAS has been possible, if not always practical, for a wide range of plant traits since the early 20th century. However, with recent development of molecular tools and genetic maps, MAS has become more attractive and practical than before. MAS may allow selecting for a trait in seasons or locations where PS is not feasible or is costly or ineffective, thus increasing the efficiency of selection and flexibility of a breeding program. MAS may be less time consuming for traits whose expressions are developmentally regulated and are phenotypically obvious only late in the season. Markers are independent of variation caused by genetic or environmental factors and this offers the advantage of permitting selection for traits such as resistance in the absence of pathogen, which is otherwise required to identify useful segregants. Trait heritability is the most important factor influencing the utility of MAS. It is suggested that MAS is most useful for traits with low-to-moderate heritability, for which PS may be less effective. However, this is true only if reliable markers for the low-heritability traits can be identified.

Gene pyramiding is a useful approach to maximize utilization of existing gene resources. MAS is an effective approach for pyramiding genes or QTLs from different sources and for different traits into elite germplasm. It has been shown that in backcross breeding programs MAS can be effective in reducing linkage drag and optimizing population size by selecting against the donor genome (i.e., background selection) while selecting for allele(s) to be introgressed from the donor parent (i.e., foreground selection). In other words, the use of MAS in a backcross breeding program can expedite transfer of desirable traits from the donor parent as well as fast recovery of the recurrent genome by breaking the undesirable linkages between traits following gene introgression from the wild species. In addition, MAS can expedite backcross breeding by allowing strict backcrossing in each generation rather than modified backcrossing (i.e., selfing after each generation of backcrossing), which is often necessary when transferring genes with recessive or additive effects. With MAS it is also possible to conduct multiple rounds of selection in a year, a gain of time of about two backcross generations per year compared to one in PS. Furthermore, MAS can speed up the breeding process by allowing seedling assays, simultaneous selection for multiple traits, and increasing the efficiency of selection by eliminating difficult trait assays.

Although the utility of MAS for manipulating single-gene traits is straightforward and has been well documented, its usefulness for complex traits also has been recognized [451–463]. However, it should be realized that MAS for polygenic trait improvement is in its infancy and transitory phase, and the field is on the verge of producing convincing results. Based on most simulation studies and empirical results, it appears that trait heritability (*h^2^*) and the number-of-QTLs are the most important factors influencing the effectiveness of MAS. MAS seems to be most effective for traits with low *h^2^* (0.1–0.3) and which are controlled by rather small numbers of QTLs with large effects. It is generally accepted that, in most cases, for a low-heritability trait MAS will give better selection results than phenotypic selection [457]. In particular, for many quantitative traits, MAS should be useful for pyramiding individual components comprising the complex trait. Thus, it would be more efficient to partition complex traits into their contributing components and identify QTLs for each component before attempting marker-assisted breeding.

### 4.1. Use of MAS in tomato breeding

The use of MAS in tomato breeding is by no means a new idea. In the early 1980s, many tomato seed companies in the U.S. and abroad took advantage of the reported linkage association between nematode resistance and *Aps-1^1^* locus [270] and used the *Aps* marker for selecting for nematode resistance. More recently, however, MAS has become a reality and to some extent a routine practice in many seed companies for improving tomatoes for many simply-inherited traits as shown in Table 6. Unfortunately, however, most of these activities are not reported in public literature. A survey by the author of some major seed companies in the U.S., including Seminis Vegetable Seeds (now owned by Monsanto), Syngenta, Harris Moran, Sakata and Asgrow, and in Europe, including Nunhems Zaden, Vilmorin, Seminis Vegetable Seeds Holland, ENZA, RijkZwaan and DeRuiter, indicated that MAS was routinely employed for tomato improvement for many qualitatively inherited disease resistance traits. Examples include vertical resistance to diseases such as corky root, fusarium wilt, late blight, root-knot nematodes, powdery mildew, bacterial speck, tobacco/tomato mosaic virus, tomato spotted wilt virus, and verticillium wilt (see Table 6). Many of these seed companies indicated that for several of the resistance traits MAS was not only faster than PS but in some cases was also cheaper and more effective. Accordingly, MAS is also practiced for improvement of tomato for some other simple-inherited traits such as jointless, ripening, and carotenoid content (lycopene and beta carotene). However, there is very little indication of the use of MAS in seed companies for manipulating QTLs for complex traits, although it seems that it is being attempted to improve quantitative resistance to bacterial wilt, bacterial canker, bacterial wilt, powdery mildew and yellow leaf curl virus as well as to improve fruit soluble solids (°Brix).

The use of MAS is much less common in public tomato breeding programs, although it has been practiced to improve vertical resistance to a few diseases such as late blight (M Mutschler, Cornell University; R Gardner, NC State University; Foolad, Penn State University), bacterial canker, bacterial speck and bacterial spot (D Francis, Ohio State University; [287, 447], and horizontal resistance to blackmold [288] and late blight [342]. It also has been used infrequently for simple inherited traits such as self pruning (e.g., in a few tomato genetics and breeding programs in the US) and complex fruit quality traits [464]. However, based on the published research, in most cases where MAS was employed to transfer QTLs there were major problems associated with the derived lines in terms of their horticultural value. For example, in case of late blight resistance where three NILs were developed each containing one resistance QTL on an introgressed interval of 6.9, 8.8, or 15.1 cM from *L. hirsutum*, while all three lines exhibited expected level of resistance, they also suffered from undesirable horticultural characteristics [342]. Further inspections of the NILs resulted in the detection of QTLs for other characteristics such as plant shape, canopy density, maturity, fruit yield, or fruit size in the same introgressed regions. The results prompted the authors to suggest further refining of the QTLs before transferring to adapted genetic backgrounds. Similar conclusions were made regarding MAS transfer of QTLs for blackmold resistance from *L. cheesmanii* to the cultivated tomato, as negative associations were found between introgressed QTL alleles and horticultural characteristics [288]. Such undesirable associations were reported for some other complex characteristics in tomato (e.g., [242, 465]), and in most cases it was not clear whether they were due to genetic linkage or pleiotropic effects. With the current state of QTL identification, it is not unexpected that similar problems would arise if practicing MAS for other complex traits in tomato. Before MAS becomes a routine procedure for improving complex traits in tomato, issues surrounding its utility must be addressed.

### 4.2. Issues in using MAS

For almost two decades, MAS has been claimed as an effective alternative to PS for crop improvement. As indicated earlier, the successful use of MAS for manipulating single-gene traits has been well documented. However, despite tremendous investment in finding markers associated with important genes and QTLs, MAS has not yet become a routine procedure in most plant breeding programs, in particular for improving complex traits. The associated problems/issues with the use of MAS are several fold, including: (1) elevated cost of high-throughput marker genotyping, which makes MAS not affordable by most breeding programs. However, with advancements in technology and availability of more ESTs, microarrays and DNA sequences, this does not seem to be a permanent problem. (2) Unavailabililty of closely-linked markers for many traits for which markers have been reported. Loosley linked markers are not useful because of crossovers between markers and the genes or QTLs of interest. This is particularly a serious problem when genes or QTLs to be transferred are found within wild species. (3) Unavailability of reliable PCR-based markers for many simple as well as complex traits. Markers that are expensive and need extensive work to determine, for examples, RFLPs or AFLPs, are not useful in most plant breeding programs where often large populations need to be screened. In tomato, PCR-based markers are available only for a handful of traits (mainly a few disease resistance traits). (4) Lack of validated QTLs for most complex traits. Generally, markers are as good as phenotypic screenings that are used to identify them. For low-heritability traits, for which MAS is clamied to be most helpful, identification of reliable QTLs is not easy unless replicated experiments are conducted across populations and environments. The utility of MAS for complex traits depends on the availability of reliable and precisely delineated QTL intervals. Despite the identification of QTLs for many traits in tomato, only for a few traits the identified QTLs have been verified. (5) Large size of QTL intervals and association with undesirable traits due to “linkage drag.” This is in particular a major problem when transferring genes or QTLs from wild species into the cultigen. (6) Limited molecular marker polymorphism within the cultivated species of tomato. Many tomato breeding programs largely exploit variation within the cultivated tomato. Lack of sufficient marker polymorphism within the cultigen has hindered the use of marker technology. However, with advancements in the marker technology and identification of more resolving DNA markers, this is not expected to be a major problem in future. (7) Unfamiliarity of many traditional plant breeders, who in fact release most of the modern cultivars, with the use of markers, or their limited access to molecular marker laboratories. (8) Identification and mapping of genes and QTLs mainly by researchers who are not breeders or do not have inherent interest in crop improvement. It seems that better cooperation between basic researchers and plant breeders is needed to coordinate meaningful identification and usage of gene/QTL-linked markers. However, from among all these issues, a primary limiting factor in the use of MAS in tomato breeding is the lack of adequate marker polymorphism in the cultivated species or between the cultivated species and closely related species such as *L. pimpinellifolium* and *L. cheesmanii*. Many tomato breeders focus primarily on exploitation of genetic variation among the elite lines or within the cultigen. A good example is the case of resistance genes for tomato bacterial spot (caused by *Xanthomonas campestris* pv. *vesicatoria*) which have been identified either in *L. esculentum* or *L. esculentum* var. *cerasiforme* [68]. In such cases, MAS cannot be easily employed using traditional molecular markers such as RFLPs, CAPS, or AFLPs. However, the current and recent development in discovering SNPs within the cultivated species of tomato are expected to reduce or rectify this problem and facilitate the use of markers in tomato breeding programs exploiting intraspecific genetic variation.

## 5. POSITIONAL CLONING OF GENES

Positional cloning has been practical in tomato because of the rather low ratio of physical-to-genetic distance (average *∼*750 kb/cM). In fact, as most tomato genes are located in the euchromatic regions of the genome, which constitue only about one fourth of the tomato genome [484], this ratio is much smaller for the genetically active fraction of the genome. Tomato was the first plant species in which a disease resistance gene, *pto*, conferring resistance to bacterial speck caused by *Pseudomonas syringae* pv. *tomato* (*Pst*), was cloned using map-based cloning approach [56]. Further analysis of this gene indicated similarity of its ORF to serine-threonine protein kinases (see Table 7; [57, 468]). Subsequently, a similar map-based cloning strategy was employed and several other tomato genes were cloned, including *Prf*, which is required for *Pto* activity and tomato resistance to *Pst* and which also confers tomato susceptibility to organophosphate insecticide Fenthion [317, 485], *Sw-5*, conferring resistance to tospovirus [483], *sp* (*self pruning*; [170]), members of *sp* gene family [169], and *j* and *j-2*, controlling jointless pedicel [476]. It should be noted that both *j* and *j-2* are recessive mutants that completely suppress the formation of pedicel abscission zones [176, 432, 476–478]. As indicated earlier, *jointless* pedicel is an essential character and widely used in the processing tomato industry as it aids mechanical harvesting and prevents physical wounding during transportation. *Jointless* pedicel is also becoming highly desirable in fresh market tomato cultivars. In additioin, during the past decade, several other major genes in tomato have been fine- mapped and/or cloned via map-based cloning approach, as shown in Table 7. Large-insert DNA libraries are essential to isolate genes and QTLs by map-based cloning. Thus, large-insert bacterial artificial chromosome (BAC) or plant-transformation-competent binary BAC (BIBAC) libraries have been constructed for several genotypes of the cultivated tomato, including cv. Mogoer [53]; (http://hbz7.tamu.edu), cv. Heinz 1706 and cv. LA3023 [52]; (http://www.genome.arizona.edu; https://www.genome.clemson.edu), and *L. pennellii* accession LA716 and *L. cheesmanii* accessions LA166 (http://hbz7.tamu.edu) and LA438 (http://www.genome.arizona.edu). A complete list of available tomato libraries can be found at the SGN (http://www.sgn.cornell.edu). The large-insert libraries have been used for different purposes in tomato genomics research, including physical mapping, map-based cloning and sequencing.

## 6. POSITIONAL CLONING OF QTLS

Most QTL experiments often detect QTLs within rather large marker intervals, usually 10 cM or greater. The genetic and physical natures of most such QTLs are not known. For example, often it cannot be determined whether a detected QTL contains one gene or several tightly linked genes affecting the same trait. Also, detected QTLs may affect more than one trait and often it is unknown whether such effects are due to pleiotropic effects of the same gene or linkage of several independent loci. In tomato, for example, the lower part of chromosome 1 has been identified to affect many agriculturally important traits, including various morphological and fruit quality characteristics as well as resistance to many biotic and abiotic stresses [405, 406]. Similarly, the long arm of chromosome 4 contains QTLs for many horticulturally important traits such as fruit soluble solids content, shape and lycopene content [304]. Furthermore, it has been shown frequently that introduction of a small segment of DNA from a tomato wild species into the cultivated tomato results in significant changes in several characteristics [234, 238, 288, 342]. Earlier QTL studies were unable to determine how many genes would control each character or whether same genes could affect more than one trait. During the past several years, however, advancements in marker technology have facilitated physical characterization of QTL segments in tomato. For example, a NIL of tomato containing a 40-cM introgression at the bottom of chromosome 1 from *L. hirsutum* accession LA1777 was dissected by developing sub-NILs containing smaller introgression segments affecting different traits [405]. In a similar study, a series of sub-NILs were developed for tomato chromosome 1 containing introgressions from *L. chmielewskii* to fine-map loci controlling a number of fruit quality characteristics important to processing tomato varieties [406]. By such substitution mapping studies, it was determined that in the lower part of tomato chromosome 1 independent genetic loci affected fruit soluble solids (°Brix), yield and fruit shape, whereas genetic factors affecting fruit weight, shoulder pigmentation, and external color coincided with the location of a °Brix locus. These results, combined with results of other studies, prompted the authors to conclude that the base of tomato chromosome 1, which exhibits significant effects on various agronomic and fruit quality characteristics, contains multiple QTLs whose effects can not be attributed to the pleiotropic effects of a single locus [405, 406]. In another study, NILs containing the lower part of chromosome 4 from either *L.* *peruvianum* or *L. hirsutum* were dissected by developing series of sub NILs containing small introgressions from either of the two wild species [304]. Results of this study indicated the presence of multiple, loci controlling soluble solids and fruit weight and other loci controlling fruit shape, fruit weight and epidermal reticulation which colocalized to the same portion of chromosome 4 and could be attributed to pleiotropy and/or gene-dense-area with low frequency of recombination. Many similar QTL coincidences have been observed in tomato and other crop species, however, in most cases the nature of such coincidences have yet to be determined.

During the past decade, efforts have been made to clone QTLs and determine whether QTLs have the same molecular basis as Mendelian genes [486]. Much of such efforts have been made in tomato as a model species. For example, the first map-based cloning of a QTL in plants was carried out in tomato for a fruit size QTL (*fw2.2*) by [305]. While tomato improvement for fruit size has been relatively easy due to its high heritability [487], the inheritance (e.g., [488]) and QTL mapping studies (e.g., [414, 465]) have revealed that this trait is controlled by many loci. To date, most, if not all, QTLs involved in the evolution of tomato fruit size (from small to large) have been identified and mapped (see Table 4). In many studies, one major QTL (known as *fw2.2*)was found to be associated with large phenotypic variation for fruit size [414, 465]. While the modern tomato cultivars carry large-fruit alleles at this locus, all wild *Lycopersicon* species examined contain small-fruit alleles [435]. Because of its large, consistently detectable effects, significant efforts were made to clone and characterize this QTL [305]. In a complementation test, when a cosmid obtained from *fw2.2* region of a small-fruited wild species (*L. pennellii*) was transformed into large fruited cultivars, it resulted in reduction in fruit size. By applying a map-based cloning approach, *fw2.2* was cloned, sequenced, and characterized [305, 473, 474]. Furthermore, it was determined that this gene was expressed early in floral development and controls carpel cell number. Following this remarkable advancement, similar strategy has been used in tomato to fine map and/or clone a few other QTLs affecting traits such as soluble solids content, fruit shape, and exserted stigma, as shown in Table 7. However, it is expected that with advancements in marker technology and QTL identification, more and more QTLs will be fine-mapped and cloned using positional cloning strategy.

## 7. TOMATO GENOME ORGANIZATION AND SEQUENCING

The tomato nuclear genome comprises 12 chromosomes and approximately 950 Mbp of DNA, containing 59% non coding sequences, 28% coding sequences, 11% transposons, and 2% organellar sequences [484]. Approximately 77% of the chromosomal DNA is comprised of centromeric heterochromatic regions, which are devoid of genes [484, 489]. The tomato genome is estimated to encode *∼*35,000 genes, majority of which are populated at distal euchromatic regions of the chromosomes [8, 46, 490] with an approximate gene density of 6.7 kb/gene, similar to that of *Arabidopsis* and rice [484]. The latter study [484] also indicates that a significant portion of the tomato euchromatin is methylated in the intergenic spacer regions. Currently the 12 tomato chromosomes are being sequenced by an international consortium of 10 countries, including China (chr. 3), France (chr. 7), India (chr. 5), Italy (chr. 12), Japan (chr. 8), Korea (chr. 2), The Netherlands (chr. 6), Spain (chr. 9), United Kingdom (chr. 4), and the U.S. (chrs. 1, 10 and 11) (http://www.sgn.cornell.edu/help/about/tomato_sequencing.pl.). This effort is part of a larger initiative known as the International Solanaceae Genome Project (SOL): Systems Approach to Diversity and Adaptation. Lunched in 2003, this project has set ambitious research goals for the next 10 years, including physical, evolutionary, and functional genomics of the family Solanaceae [491]; (http://www.sgn.cornell.edu/solanaceae-project/index.pl). The first cornerstone of the project, however, is to determine a high-quality sequence for the euchromatic portions of the tomato chromosomes as a reference for the Solanaceae. To date (March 2007), about 17% of the target regions have been sequenced (http://sgn.cornell.edu). Concomitantly, other genome organizations studies are being conducted in tomato, including efforts to expand EST database of tomato. To date, more than 214 000 ESTs have been developed (http://compbio.dfci.harvard.edu/tgi/cgi-bin/tgi/gimain.pl?gudb=tomato). Although the EST-derived unigene sets of tomato do not represent the entire gene repertoire of this species, analysis of the tomato EST database and several sequenced BAC libraries have led to the prediction that tomato genome encodes *∼*35 000 genes, largely sequestered in euchromatic regions of the 12 tomato chromosomes, which correspond to less than 25% of the total nuclear DNA in tomato [8, 484]. Recently, Syngenta has mapped 17 000 BACs to the *L. esculentum* × *L. pennellii* ILs and made the data available to the tomato sequencing project (ftp://ftp.sgn.cornell.edu/tomato_genome/bacs/syngenta).

## 8. CONCLUSION AND FUTURE PROSPECTS

During the past two decades, remarkable progress has been made in tomato molecular marker research, including development of markers and maps, mapping of genes and QTLs, comparative analysis, generation of large insert libraries, fine-mapping and map-based cloning of genes and QTLs, and genome sequencing and organization. A primary goal of molecular mapping has been to use markers as indirect selection criteria for crop improvement. Comparatively, however, little has been reported as to the actual use of markers in tomato breeding, in particular for improvement of complex traits. Potential reasons for this deficiency were discussed above. However, based on most recent discoveries and research progresses, it seems that the prospect for routine application of markers in tomato breeding in the future is good. Perhaps the most important factor is development of markers that are more resolving and easier to use in breeding programs. High-throughput marker systems that are easy to assay, PCR based, and can detect polymorphism between closely related genotypes are forthcoming. In particular, it is expected that more SNP markers will be available, which will detect polymorphism among elite tomato germplam and will gain utility in marker-assisted breeding in tomato. It is also expected that with further advancements in molecular marker technology, the cost of marker development will continue to decline, making them more economical. Furthermore, as the sequencing of the tomato genome progresses, the information will be used to develop additional sequence-based high-resolving markers. It is also expected that a greater emphasis will be placed on development of functional markers, including PCR-based ESTs and candidate genes, which will be highly useful to both applied and basic research programs. Thus, it is not unlikely that in a near future MAS becomes a routine procedure in many tomato breeding programs, in particular for improvement of many simply inherited traits. Many breeders are convinced that even for many simple traits with high heritability MAS has an edge over PS because of various potential limitations in phenotypic screening. Current use of MAS for improvement of many such traits in commercial seed companies, where funding is often less limited than in public tomato breeding programs, supports this assessment.

Unlike for simple traits, there is little evidence of the use of markers for improving complex characteristics in tomato. Two major limiting factors are unrialiability of QTLs and linkage drag, as discussed above. For many complex traits, such as yield or tolerance to abiotic stresses, obtaining reliable phenotypic data for QTL mapping is not straightforward, often leading to the identification of QTLs which may not be reproducible and thus of little value. Improvements ought to be made in our ability to identify more tractable QTLs for complex traits. One approach is to identify QTLs controlling individual components of complex genetic variation, rather than detecting QTLs based on the ultimate trait(s). For example, partitioning of the total genetic variation for a complex trait into its physiological and developmental components would lead to detection of QTLs for individual components, which may be more tractable and useful. A subsequent necessary step to streamline the use of QTLs is to further refine QTL positions by development of NILs and sub-NILs. Such fine mapping may not only establish the actual value of individual QTLs, but also may determine whether any potential negative association with undesirable genes could be broken before transferring QTLs. In other words, fine mapping would allow detection of QTLs that are useful for marker-assisted breeding. The importance of such refinements is well recognized among geneticists and breeders and many research programs have initiated such activities. It is the author's expectation that in a not-too-distant future we will witness a greater application of marker technology to tomato crop improvement for simple as well as complex characteristics. Another reason to be optimistic is the increasing use of F_1_ hybrid cultivars for commercial production. When developing hybrids, the use of MAS will not only be more practical but also more economical. However, despite all expected advancements in the marker technology, I do not anticipate that MAS will be a “silver bullet” solution to every breeding problem in tomato. Most likely, in future, a combination of traditional breeding protocols and marker-assisted breeding will become a routine procedure for tomato crop improvement.

## Figures and Tables

**Table 1:** Genetic linkage maps of tomato (*Lycopersicon* spp.) developed based on different intra- and interspecific crosses.

Linkage map	Population type^ (a) ^	Population size	Number of markers	Type of markers^ (b) ^	Reference
*L. esculentum* × *L. esculentum* var. *cerasif.*
1. Cervil × Levovil	F_7_-RIL	153	377	RFLP, RAPD, AFLP	[215]

*L. esculentum* × *L. pimpinellifolium*
1. M82-1-7 × LA1589	BC_1_	257	120	RFLP, RAPD, morphological	[166]
2. NC84173 × LA722	BC_1_	119	151	RFLP	[230]
3. Giant Heirloom × LA1589	F_2_	200	90	RFLP, CAPS	[40]
4. E6203 × LA1589	BC_2_F_6_-BIL	196	127	RFLP	[231]
5. NC84173 × LA722	F_10_RIL	119	191	RFLP, RGA	Foolad et al. (unpubl.)
6. NCEBR1 × PSLP125	F_2_	172	256	RFLP, EST, RGA	Foolad et al. (unpubl.)
7. NCEBR1 × PSLP125	F_8_-RIL	172	255	RFLP, EST	Foolad et al. (unpubl.)

*L. esculentum* × *L. cheesmanii*
1. UC204B × LA483	F_2_	350	71	RFLP	[232]
2. UC204B × LA483	F_7_-RIL	97	132	RFLP	[233]

*L. esculentum* × *L. parviflorum*
1. E6203 × LA2133	BC_2_	170	133	RFLP, SCAR, morphological	[234]

*L. esculentum* × *L. chmielewskii*
1. UC82B × LA1028	BC_1_	237	70	RFLP, Isozyme	[167]

*L. esculentum* × *L. hirsutum*
1. E6203 × LA1777	BC_1_	149	135	RFLP	[235]
2. E6203 × LA1777	NIL, BIL	111	95	RFLP	[236]
3. NC84173 × PI126445	BC_1_	145	171	RFLP, RGA	[217]

*L. esculentum* × *L. pennellii*
1. VF36 *Tm2* ^ (a) ^ × LA716 (high-density map of tomato)	F_2_	67	1050	Isozyme, RFLP, morphological	[228, 229]
2. Vendor *Tm2* ^ (a) ^ × LA716	F_2_	432	98		[237]
3. M82 × LA716	IL	50	375	RFLP	[238]
4. VF36 *Tm2* ^ (a) ^ × LA716	F_2_	67	1242	AFLP, RFLP	[218]
5. E6203 (LA925) × LA716	F_2_	83	1500	COS	[198]
6. E6203 × LA1657	BC_2_	175	110	RFLP	[239]
7. E6203 × LA716	F_2_	83	152	SSRs, CAPs	[207]

*L. esculentum* × *L. peruvianum*
1. E6203 × LA1706	BC_3_	241	177	RFLP, SCAR	[240]

*L. esculentum* var. *cerasif. × L. pimpinellifolium*
1. E9 × L5	F_6_-RIL	142	132	SSR, SCAR	[211]

*L. esculentum* var. *cerasif.* × *L. cheesmanii*
1. E9 × L3	F_6_-RIL	115	114	SSR, SCAR	[211]

*L. peruvianum* × *L. peruvianum*
1. LA2157 × LA2172	BC_1_	152	73	RFLP	[241]

^(a) ^RIL: recombinant inbred line; NIL: near isogenic line; BIL: backcross inbred line.

^(b) ^RFLP: restriction fragment length polymorphism; RAPD: randomly amplified polymorphic DNA; AFLP: amplified fragment length polymorphism; CAPS: cleaved amplified polymorphic sequence; RGA: resistance gene analog; EST: expressed sequence tag; SCAR: sequence characterized amplified region; SSR: simple sequence repeat.

**Table 2:** Summary of disease (fungal, bacterial, viral, and nematode) and insect resistance genes and QTLs (Q) mapped on tomato chromosomes.

Disease	Gene/QTL	Pathogen	Resistance source	Mapping population	Chromosomal location	References
Alternaria stem canker	*Asc*	*Alternaria alternata* f. sp. *lycopersici*	*L. pennellii*	F_2_	3	[309, 310]

Anthracnose ripe rot	*Anthracnose* (Q)	*Colletotrichum coccodes*	*L. esculentum*	F_2_	Various Chromosomes	[67]

Aphid (potato)	*Meu-1*	*Macrosiphum euphorbiae*	*L. peruvianum*	NIL F_2_	6	[38, 311, 312]

	*Rcm 1.0–10.0* (Q)	*Calvibacter michiganensis* ssp. *michiganensis*	*L. peruvianum*	BC_1_	1,6,7,8,9,10	[313]
Bacterial canker	*Rcm5.0, Rcm7.1, Rcm9.0 (Q)*	*Calvibacter michiganensis* ssp. *michiganensis*	*L. peruvianum*	F_2_	5,7,9	[314]
	*Rcm2.0, Rcm5.1* (Q)	*Calvibacter michiganensis* ssp. *michiganensis*	*L. hirsutum*	BC_2_S_5_	2,5	[287, 315]

Bacterial speck	*Pto*	*Pseudomonas syringae* pv. *Tomato (Pst)*	*L. pimpinellifolium*	NIL F_2_	5	[57, 316]
	*Prf*	Required for resistance to *Pst*	*L. pimpinellifolium*	NIL F_2_	5	[317]

Bacterial spot	*Rx-1, Rx-2, Rx-3, Rx-4 (Q) Bs4, Xv4*	*Xanthomonas euvesicatoria, X. vesicatora, X. perorans, X. gardneri*	*L. esculentum L. pennellii*	BC_1_, F_2_, BC_3_	1,3,4,5	[68, 69, 318, 319]

Bacterial wilt	*Rrs 3.0–12.0* (Q)	*Ralstonia solanacearum*	*L. pimpinellifolium L. peruvianum*	F_2_, F_3_	3,4,6,7,10	[320–324]

Blackmold	*Blackmold* (Q)	*Alternaria alternata*	*L. cheesmanii*		2,3,9,12	[288]

Corky root rot	*Py-1*	*Pyrenochaeta lycopersici*	*L. peruvianum*	NIL F_2_	3	[325]

Cucumber mosaic virus	*Cmr*	CMV	*L. chilense*	BC_1_-inbred	12	[326]

Early blight	*11 (Q)*	*Alternaria solani*	*L. hirsutum*	BC_1_	1,2,5,8,9,10,11,12	[242]
	*13 (Q)*	*Alternaria solani*	*L. hirsutum*	BC_1_S_1_	1,2,3,5,8,9,10,11,12	[242]
	*7 (Q)*	*Alternaria solani*	*L. hirsutum*	BC_1_	1,2,3,5,8,9,10,11,12	[243]
	*7 (Q)*	*Alternaria solani*	*L. pimpinellifolium*	RILs	1,3,4,5,6,9,11	Foolad et al. (unpubl.)

Fusarium crown and root rot	*Frl*	*Fusarium oxysporum* f. sp. *radidicis-lycopersici*	*L. peruvianum*	F_2_	9	[327]

Fusarium wilt	*I, I1, I2, I2C, I3*	*Fusarium oxysporum* f. sp. *lycopersici*	*L. pimpinellifolium L. pennellii*	Different populations	7,8,11	[328–336]

Gray leaf spot	*Sm*	*Stemphylium* spp.	*L. pimpinellifolium*	F_2_	11	[337]

Late blight	*Ph-1, Ph-2, Ph-3*	*Phytophthora infestans*	*L. pimpinellifolium*		7,9,10	[338–340]
	*lb1-lb12* (Q)	*Phytophthora infestans*	*L. hirsutum L. pimpinellifolium*		All 12 Chromosomes	[341–343]

Leaf mould	*Cf-1, Cf-2, Cf-4, Cf-5, Cf-9, Cf-ECP2*	*Cladosporium fulvum*	*L. hirsutum L. pimpinellifolium*	F_2_, NIL F_2_, BC_1_	1,6	[218, 344–347]

Nematode (potato cyst)	*Hero*	*Globodera restochiensis*	*L. pimpinellifolium*	NIL F_2_	4	[348]

Nematode (root knot)	*Mi, Mi-1, Mi-2, Mi-3, Mi-9*	*Meloidogyne* spp.	*L. peruvianum*	F_2_, F_3_, NIL F_2_, BC_1_, BC_2_	6,12	[312, 349–355]

Potyviruses	*pot-1*	Potyviruses	*L. hirsutum*	ILs	3	[356]

Powdery mildew	*Lv*	*Leveillula taurica*	*L. chilense*	F_2_	12	[357]
	*Ol-1, Ol-2*	*Oidium lycopersicum*	*L. esculentum L. hirsutum*	F_2_	4,6	[219, 358, 359]
	*Ol* (Q)-*1, Ol* (Q)-*2, Ol* (Q)-*3*	*Oidium lycopersicum*	*L. parviflorum*	F_2_	6,12	[360]

Tobacco mosaic virus	*Tm-1, Tm-2* ^ (a) ^	TMV/ToMV	*L. hirsutum L. peruvianum*	F_2_, NILs	2,9	[327, 361–364]

Tomato mottle virus	One *Gene*	ToMoV	*L. chilense*	F_2_	6	[365]

Tomato spotted wilt virus	*Sw-5*	TSWV	*L. peruvianum*	NILs, F_2_	9	[366]

Tomato yellow leaf curl virus	*Ty-1* (Q), two other Q	TYLCV	*L. esculentum L. chilense L. pimpinellifolium L. hirsutum*	BILs, F_2_, F_3_, F_4_	6,11	[289, 367, 368]
	*Ty-2*	TYLCV	*L. hirsutum*	F_6_RILs	11	[369]
	*Ty-3*	TYLCV	*L. chilense*	F_2_NIls	6	[370]

Verticillium wilt	*Ve*	*Verticillium dahliae*	*L.cheesmanii*	F_2_, RILs, ILs	9	[282, 371]
			*L. esculentum*			
			*L. pennellii*			

**Table 3:** Summary of abiotic stress tolerance/resistance QTLs mapped on tomato chromosomes.

Stress	Specific trait^(a)^	Number of QTLs	Tolerance source	Mapping population	Chromosomal location	References
Cold (low temp.)	SG	3	*L. pimpinellifolium*	BC_1_S_1_	1,4	[378]
		5	*L. pimpinellifolium*	RILs	1,2,3,8,12	Foolad et al. (unpubl.)
	VG	3	*L. hirsutum*	BC_1_	6,7,12	[275]
	Sht. wlt., RAU	10	*L. hirsutum*	BC_1_	1,3,5,6,7,9,11,12	[379]

Drought	SG	4	*L. pimpinellifolium*	BC_1_S_1_	1,8,9,12	[86]
		8	*L. pimpinellifolium*	RILs	1,2,3,4,8,9,12	Foolad et al. (unpubl.)
	WUE	3	*L. pennellii*	BC_1_S_1_, F_3_	Undetermined	[380]

Salt	SG	5	*L. pennellii*	F_2_	1,3,7,8,12	[381]
		8	*L. pennellii*	F_2_	1,2,3,7,8,9,12	[382]
		8	*L. pennellii*	F_2_	1,3,5,6,8,9	[383]
		7	*L. pimpinellifolium*	BC_1_S_1_	1,2,5,7,9,12	[95]
		8	*L. pimpinellifolium*	RIL_s_	1,2,3,4,8,9,12	Foolad et al. (unpubl.)
	VG	4	*L. pimpinellifolium*	BC_1_S_1_	1,5,9	[384]
		5	*L. pimpinellifolium*	BC_1_	1,3,5,6,9	[385]
		7	*L. pimpinellifolium*	RILs	1,3,4,5,7,8,9	Foolad et al. (unpubl.)
	Ion accumulation	6	*L. pennellii*	F_2_	1,2,4,5,6,12	[386].
	FN, FW, FY	Several	*L. pimpinellifolium*	F_2_	Undetermined	[387]

^(a) ^FN: fruit number; FW: fruit weight; FY: fruit yield; RAU: root ammonium uptake; SG: seed germination; Sht. wlt.: shoot wilting; VG: vegetative growth; WUE: water use efficiency.

**Table 4:** Summary of flower, fruit, and yield-related characteristics for which genes or QTLs have been identified and mapped in tomato.

Trait^(1)^	Wild species used	Mapping population	Genes (G) or QTLs (Q)	Chromosome	References
Anther-tube width	*L. pimpinellifolium*	BC_1_	2 Q	6,7	[208]

Anther-tube length	*L. pimpinellifolium*	BC_1_	2 Q	2,7	[208]

Carotenoid biosynthesis candidate genes	*L. pennellii*	ILs	23 G	Most chromosomes	[294]

Corolla indentation	*L. hirsutum*	BC_1_	2 Q	2,8	[235]

Flwr., exerted stigma	*L. pennellii*	BC_1_	4 Q	1,2,7	[274]
	*L. hirsutum*	BC_1_	1 Q	12	[235]
	*L. peruvianum*	BC_3_, BC_4_	2 Q	2,9	[240]

Flwr., petal apex angle	*L. pennellii*	F_2_	1 Q	11	[404]

Flwr., petal length	*L. pennellii*	F_2_	2 Q	7,12	[404]

Flwr., petal number	*L. pennellii*	F_2_	1 Q	11	[404]

Flwr., petal surf. area	*L. pennellii*	F_2_	1 Q	12	[404]

Flwr., petiolule length	*L. pennellii*	F_2_	2 Q	7,12	[404]

Flwr., sepal apex angle	*L. pennellii*	F_2_	2 Q	5,9	[404]

Flwr., sepal D/L ratio	*L. pennellii*	F_2_	3 Q	5,8,11	[404]

Flwr., sepal number	*L. pennellii*	F_2_	2 Q	2,11	[404]

Flwr., sepal surf. area	*L. pennellii*	F_2_	1 Q	3	[404]

Flwr., sepal width	*L. pennellii*	F_2_	1 Q	3	[404]

Flwr., sepal W/L ratio	*L. pennellii*	F_2_	2 Q	5,8	[404]

Frt. antioxidant capacity	*L. pennellii*	ILs	5 Q	3,6,7,10	[300]

Frt. ascorbic acid	*L. pennellii*	ILs	6 Q	3,5,10,12	[300]

Frt. citric acid content	*L. pennellii*	ILs	7 Q	4,5,8,9,10	[297]

Frt. color	*L. pimpinellifolium*	BC_1_	2 Q	2,6	[208]
	*L. pimpinellifolium*	BC_2_, BC_3_	5 Q	2,3,4,7,8	[286]
	*L. peruvianum*	BC_3_, BC_4_	8 Q	1,6,7,8,9,10,12	[240]
	*L. hirsutum*	BC_2_, BC_3_	15 Q	1,2,4,5,7,8,9,10,11	[283]
	*L. hirsutum*	subNILs	1 Q	1	[405]
	*L. parviflorum*	BC_3_	9 Q (visual test)	1,2,4,5,7,8,11,12	[234]
			4 Q (spec. test)	4	[234]
	*L. chmielewskii*	NILs	1 Q	1	[406]
	*L. hirsutum*	NILs	1 Q	1	[406]
	*L. pennellii*	BC_2_/BC_2_F_1_	1 Q	12	[239]

Frt. color ( *β*-carotene)	*L. cheesmannii L. hirsutum L. pennellii*	F_2_, ILs	1 G (*B*)	6	[131, 220, 407–409]
	*L. parviflorum*	BC_3_	6 Q	2,4,8,9,10,11	[234]
	*L. pennellii*	ILs	1 G (*B*)	6	[220, 299]
	*L. pennellii*	ILs	2 Q	6	[300]

Frt. color (carotene content)	*L. esculentum*	RIL	3 Q	2,3,8	[410]

Frt. color (crimson)	*L. esculentum*	F_2_	1 G (*og^c^, cr*)	6	[131–133, 299, 411]

Frt. color (external)	*L. parviflorum*	BC_3_	9 Q	1,2,4,5,7,8,11,12	[234]
	*L. pimpinellifolium*	BC_2_F_6_	2 Q	3,11	[231]
	*L. pennellii*	BC_2_/BC_2_F_1_	2 Q	5,12	[239]

Frt. color (high pigment 1)	*L. esculentum, L. cheesmannii*	F_2_	1 G (*hp-1*)	2	[120, 412]

Frt. Color (high pigment 2)	*L. esculentum, L. pennellii*	BC_1_	1 G (*hp-2*)	1	[121, 413]

Frt. color (internal)	*L. parviflorum*	BC_3_	15 Q	1,2,4,5,7,8,9,10,11,12	[234]
	*L. pimpinellifolium*	BC_2_F_6_	3 Q	3,11	[231]
	*L. pennellii*	BC_2_/BC_2_F_1_	1 Q	12	[239]
	*L. pennellii*	ILs	16 Q	Most chromosomes	[294]

Frt. color (lycopene)	*L. pimpinellifolium*	BC_1_S_1_	8 Q	1,4,5,6,7,10,12	[414]
	*L. parviflorum*	BC_3_	5 Q	2,3,5,8,12	[234]
	*L. esculentum*	RIL	2 Q	4,11	[410]
	*L. pennellii*	ILs	2 Q	3,6	[300]

Frt. color (orange)	*L. pennellii*	BC_2_/BC_2_F_1_	2 Q	11,12	[239]

Frt. color (yellow)	*L. parviflorum*	BC_3_	1 Q	12	[234]

Frt. cracking	*L. pennellii*	BC_2_/BC_2_F_1_	5 Q	2,5,8,10,12	[239]

Frt. cracking (concentric)	*L. pimpinellifolium*	BC_2_F_6_	3 Q	2,8,9	[231]

Frt. cracking (radial)	*L. pimpinellifolium*	BC_2_F_6_	2 Q	2,9	[231]

Frt. diameter	*L. pimpinellifolium*	BC_1_	3 Q	1,2,8	[208]
	*L. pimpinellifolium*	BC_1_S_1_	8 Q	1,2,3,6,7,11	[414]
	*L. pimpinellifolium*	F_2_	7 Q	1,2,3,4,7,11	[40]
	*L. esculentum*	RIL	5 Q	2,3,11,12	[410]
	*L. pimpinellifolium*	BC_2_F_6_	12 Q	2	[231]

Frt. elasticity	*L. esculentum*	RIL	5 Q	1,2,3,4,9	[410]

Frt. epidermal reticulation	*L. parviflorum*	BC_3_	4 Q	4,6,8,12	[234]

Frt. firmness	*L. pimpinellifolium*	BC_2_, BC_3_	4 Q	2,3,4,8	[286]
	*L. peruvianum*	BC_3_, BC_4_	6 Q	1,3,4,6,9,11	[240]
	*L. hirsutum*	BC_2_, BC_3_	3 Q	2,5,11	[283]
	*L. parviflorum*	BC_3_	12	1,3,5,6,8,9,10,11,12	[234]
	*L. esculentum*	RIL	2 Q	4,9	[410]
	*L. pimpinellifolium*	BC_2_F_6_	2 Q	6	[231]
	*L. chmielewskii*	NILs	1 Q	1	[406]
	*L. pennellii*	BC_2_/BC_2_F_1_	3 Q	2,10	[239]

Frt. fructose content	*L. pennellii*	ILs	4 Q	4,5,7,9	[297]

Frt. fructose:glucose ratio	*L. hirsutum L. pennellii*	F_2_, F_3_, BC_1_, ILs	1 G (*Fgr*)	4	[141]

Frt. glucose content	*L. pennellii*	ILs	4 Q	4,5,9,12	[297]

Frt. graywall	*L. pennellii*	BC_2_/BC_2_F_1_	1 Q	12	[239]

Frt. green gel	*L. pennellii*	BC_2_/BC_2_F_1_	3 Q	1,5,8	[239]

Frt. length	*L. pimpinellifolium*	BC_1_S_1_	9 Q	1,2,3,6,7,9,12	[414]
	*L. pimpinellifolium*	F_2_	7 Q	1,2,3,4,9,11	[40]
	*L. pimpinellifolium*	BC_2_F_6_	5 Q	2,3,8,9,11	[231]

Frt. locule number	*L. pimpinellifolium*	BC_1_	2 Q	1,3	[208]
	*L. pimpinellifolium*	F_2_	3 Q	2,11	[40]
	*L. pimpinellifolium*	F_2_	5 Q	2,3,4,10,12	[404]

Frt. malic acid content	*L. pennellii*	ILs	5 Q	3,4,8,12	[297]

Frt. maturity	*L. pimpinellifolium*	BC_2_, BC_3_	7 Q	2,3,5,7,8,9	[286]
	*L. peruvianum*	BC_3_, BC_4_	5 Q	3,5,7,8,9	[240]
	*L. hirsutum*	BC_2_, BC_3_	6 Q	3,5,7,8,9,12	[283]
	*L. parviflorum*	BC_3_	16	1,2,3,5,6,7,8,9,10,12	[234]
	*L. pimpinellifolium*	BC_2_F_6_	1 Q	7	[231]

Frt. organoleptic quality	*L. esculentum*	RIL	Many Q	Various chromos.	[143, 410, 415]

Frt. ostwald	*L. parviflorum*	BC_3_	1 Q	6	[234]

Frt. pericarp thickness	*L. pimpinellifolium*	BC_1_	4 Q	2,8,10	[208]
	*L. parviflorum*	BC_3_	7 Q	1,6,7,8,9,10	[234]
	*L. chmielewskii*	NILs	1 Q	1	[406]
	*L. pennellii*	BC_2_/BC_2_F_1_	2 Q	10,12	[239]

Frt. pH	*L. chmielewskii*	BC_1_, BC_2_	5 Q	3,6,7,8,10	[167]
	*L. cheesmanii*	F_2_, F_3_	9 Q	1,3,4,6,7,8,10	[232]
	*L. chmielewskii*	BILs/BC_2_F_5_	1 Q	7	[101, 416]
	*L. pimpinellifolium*	BC_2_, BC_3_	5 Q	1,3,5,7,12	[286]
	*L. peruvianum*	BC_3_, BC_4_	6 Q	2,3,9,10,12	[240]
	*L. hirsutum*	BC_2_, BC_3_	10 Q	1,2,3,4,6,8,9,10,12	[283]
	*L. pimpinellifolium*	BC_1_S_1_	6 Q	1,2,4,5,9,12	[414]
	*L. parviflorum*	BC_3_	10 Q	2,3,4,5,6,7,9,12	[234]
	*L. esculentum*	RIL	2 Q	11,12	[410]
	*L. pennellii*	BC_2_/BC_2_F_1_	2 Q	3,12	[239]
	*L. pennellii*	ILs	11 Q	2,4,5,8,9,10,11,12	[297]

Frt. phenolic content	*L. pennellii*	ILs	9 Q	3,5,6,7,8,9	[300]

Frt. puffiness	*L. pimpinellifolium*	BC_2_, BC_3_	5 Q	2,8,9,11	[286]
	*L. peruvianum*	BC_3_, BC_4_	1 Q	9	[240]
	*L. hirsutum*	BC_2_, BC_3_	1 Q	4	[283]
	*L. parviflorum*	BC_3_	13 Q	2,3,4,5,7,8,9,10,11,12	[234]
	*L. pimpinellifolium*	BC_2_F_6_	3 Q	8,9	[231]
	*L. pennellii*	BC_2_/BC_2_F_1_	4 Q	2,3,10	[239]

Frt. reducing sugar	*L. pennellii*	ILs	13 Q	1,2,4,5,7,8,9,10,11,12	[297]

Frt. ripening	*L. esculentum*	F_2_	2 Q	5,12	[417]
	*L. pennellii*	F_2_	Many loci	All chromosomes	[160, 418]
	*L. pimpinellifolium*	BC_1_	3 Q	2,8,9	[208]
	*L. pimpinellifolium*	BC_2_, BC_3_	4 Q	2,4,7,8	[286]
	*L. peruvianum*	BC_3_, BC_4_	4 Q	2,3,8,9	[240]

Frt. ripening (*alcobaca*)	*L. esculentum*	F_2_	*alc*	10	[159]
	*L. pimpinellifolium*	BC_1_	*alc*	10	[160]

Frt. ripening (*colorless nonripening*)	*L. esculentum, L. cheesmanii*	F_2_	*Cnr*	2	[419, 420]

Frt. ripening (*never ripe*)	*L. esculentum, L. cheesmanii*	F_2_	*Nr*	9	[421–423]

Frt. ripening (*nonripening*)	*L. pennellii L. cheesmanii*	F_2_	*nor*	10	[153, 155, 158, 424, 425]

Frt. ripening (*polygalacturonase*)	*L. pimpinellifolium*	BC_1_	TOM6	10	[160]

Frt. ripening (*ripening-inhibitor*)	*L. pennellii L. cheesmanii*	F_2_	*rin*	5	[153, 155, 158, 424, 425]

Frt. ripening (*uniform ripening*)	*L. pimpinellifolium*	BC_1_	*u*	10	[160]

Frt. rotten	*L. pimpinellifolium*	BC_2_F_6_	4 Q	2,8,9	[231]
	*L. pennellii*	BC_2_/BC_2_F_1_	5 Q	3,5,8,9,12	[239]

Frt. sensory attributes	*L. esculentum*	RIL	Many Q	Various chromosomes	[143, 415]

Frt. set (fertility)	*L. pimpinellifolium*	BC_2_F_6_	3 Q	4,5,7	[231]
	*L. pennellii*	BC_2_/BC_2_F_1_	3 Q	3,9,12	[239]

Frt. shape	*L. pimpinellifolium*	BC_2_, BC_3_	4 Q	1,2,8	[286]
	*L. pimpinellifolium*	BC_1_	2 Q	2,8	[208]
	*L. peruvianum*	BC_3_, BC_4_	12 Q	1,2,6,7,8,9,10,12	[240]
	*L. hirsutum*	BC_2_, BC_3_	9 Q	2,3,7,8,9,11,12	[283]
	*L. pimpinellifolium*	BC_1_S_1_	4 Q	1,9,10,12	[414]
	*L. hirsutum*	subNILs	1 Q	1	[405]
	*L. parviflorum*	BC_3_	16 Q	All 12 chromosomes	[234]
	*L. pimpinellifolium*	F_2_	1 Q	11	[40]
	*L. pimpinellifolium*	BC_2_F_6_	2 Q	1,9	[231]
	*L. pimpinellifolium*	F_2_	4 Q	2,3,7,11	[426]
	*L. pennellii*	BC_2_/BC_2_F_1_	4 Q	2,8,10,12	[239]

Frt. shape (bumpiness)	*L. pimpinellifolium*	F_2_	3 Q	8,9,11	[427]

Frt. shape (bell pepper)	*L. pimpinellifolium*	F_2_	3 Q	2,8	[427]
	*L. pennellii*	F_2_	1 Q	2	[428]

Frt. shape (blossom-end blockiness I)	*L. pimpinellifolium*	F_2_	1 Q	2	[427]

Frt. shape (elongated)	*L. pimpinellifolium*	F_2_	2 Q	6,9	[427]

Frt. shape (heart)	*L. pimpinellifolium*	F_2_	4 Q	1,2,3,7	[427]

Frt. shape (pear)	*L. pimpinellifolium*	F_2_	2 Q	2,10	[428]

Frt. shape (stem-end blockiness)	*L. pimpinellifolium*	F_2_	6 Q	1,2,3,7,8,12	[427]

Frt. shoulder pigmentation	*L. hirsutum*	subNILs	1 Q	1	[405]
	*L. parviflorum*	BC_3_	1 Q	10	[234]

Frt. size	*L. pennellii*	BC_2_/BC_2_F_1_	4 Q	2,3,10,12	[239]

Frt. skin reticulation	*L. pennellii*	BC_2_/BC_2_F_1_	4 Q	2,4,5,8	[239]

Frt. soluble solids (SS)	*L. chmielewskii*	NIL F_2_	1 Q	Undetermined	[429]
	*L. chmielewskii*	BC_1_, BC_2_	4 Q	3,4,6,7	[167, 430]
	*L. cheesmanii*	F_2_, F_3_	7 Q	2,3,6,7,9	[232]
	*L. chmielewskii*	BILs/BC_2_F_5_	3 Q	7,10	[101]
	*L. pennellii*	ILs	3 Q	1,5,7	[298]
	*L. chmielewskii*	BC_2_F_5_	1 Q	7	[101, 416]
	*L. cheesmanii*	F_8_ RIL	12 Q	1,2,3,4,5,6,9,10	[279]
	*L. pennellii*	ILs	23 Q	Most chromosomes	[238]
	*L. pimpinellifolium*	BC_2_, BC_3_	12 Q	2,3,4,5,6,7,8,11,12	[286]
	*L. pimpinellifolium*	BC_1_	3 Q	3,6,9	[208]
	*L. peruvianum*	BC_3_, BC_4_	9 Q	1,2,7,8,9,10	[240]
	*L. hirsutum*	BC_2_, BC_3_	5 Q	3,5,6,9	[283]
	*L. pimpinellifolium*	BC_1_S_1_	13 Q	1,2,3,7,10,12	[414]
	*L. hirsutum*	subNILs	1 Q	1	[405]
	*L. parviflorum*	BC_3_	5 Q	4,5,6,9	[234]
	*L. esculentum*	RIL	3 Q	2,9	[410]
	*L. pimpinellifolium*	BC_2_F_6_	2 Q	8,9	[231]
	*L. chmielewskii*	NILs	1 Q	1	[406]
	*L. hirsutum*	NILs	1 Q	1	[406]
	*L. pennellii*	BC_2_/BC_2_F_1_	3 Q	4,9,12	[239]
	*L. pennellii*	ILs	9 Q	1,3,4,5,7,9,10,12	[297]

Frt. SS × red yield	*L. pennellii*	ILs	14 Q	Most chromosomes	[238]
	*L. pimpinellifolium*	BC_2_, BC_3_	4 Q	3,7,9	[286]
	*L. peruvianum*	BC_3_, BC_4_	9 Q	1,2,5,7,8,9,10,12	[240]
	*L. hirsutum*	BC_2_, BC_3_	9 Q	1,2,3,4,6,8,11,12	[283]
	*L. parviflorum*	BC_3_	2 Q	5,8	[234]
	*L. pennellii*	BC_2_/BC_2_F_1_	4 Q	3,5,12	[239]

Frt. stem release (%)	*L. pimpinellifolium*	BC_2_, BC_3_	5 Q	1,2,3,10	[286]
	*L. peruvianum*	BC_3_, BC_4_	5 Q	2,6,9,12	[240]
	*L. parviflorum*	BC_3_	6	2,6,7,8,10	[234]

Frt. stem retention (%)	*L. hirsutum*	BC_2_, BC_3_	6 Q	2,8,9,10,11	[283]
	*L. pennellii*	BC_2_/BC_2_F_1_	9 Q	2,3,4,6,9,10,11,12	[239]

Frt. stem scar size	*L. parviflorum*	BC_3_	11 Q	2,3,4,5,6,7,8,9,10,11	[234]
	*L. pimpinellifolium*	BC_2_F_6_	7 Q	1,2,3,4,6,7,8	[231]
	*L. chmielewskii*	NILs	1 Q	1	[406]
	*L. hirsutum*	NILs	1 Q	1	[406]

Frt. stem scar penetration (veins)	*L. parviflorum*	BC_3_	2	1,6	[234]
	*L. pennellii*	BC_2_/BC_2_F_1_	2 Q	4,8	[239]

Frt. sugar content	*L. esculentum*	RIL	5 Q	2,3,10,11	[410]
	*L. esculentum* var. *cerasifomee*	F_2_	6 Q	ND	[112]

Frt. sunscald	*L. pimpinellifolium*	BC_2_, BC_3_	2 Q	7,8	[286]
	*L. peruvianum*	BC_3_, BC_4_	4 Q	2,3,8,9	[240]

Frt. titratable acidity	*L. esculentum*	RIL	6 Q	1,2,3,9,12	[410]
	*L. pennellii*	ILs	15 Q	2,3,4,5,7,8,9,10,11,12	[297]

Frt. total acid	*L. parviflorum*	BC_3_	4 Q	3,4,7,8	[234]

Frt. total organic acid	*L. parviflorum*	BC_3_	2 Q	9,12	[234]

Frt. viscosity	*L. pimpinellifolium*	BC_2_, BC_3_	1 Q	9	[286]
	*L. peruvianum*	BC_3_, BC_4_	4 Q	1,2,8,9	[240]
	*L. hirsutum*	BC_2_, BC_3_	2 Q	2,10	[283]
	*L. parviflorum*	BC_3_	3 Q	2,9,10	[234]
	*L. pennellii*	BC_2_/BC_2_F_1_	4 Q	2,3,9,12	[239]

Frt. weight	*L. pennellii*	BC_1_	5 Q	2,4,8	[274]
	*L. chmielewskii*	BC_1_, BC_2_	6 Q	1,4,6,7,9,11	[167, 430]
	*L. cheesmanii*	F_2_, F_3_	11 Q	1,2,3,4,6,7,9,11,12	[232]
	*L. cheesmanii*	F_8_ RIL	13 Q	1,2,3,4,6,7,9,12	[279]
	*L. pennellii*	ILs	18 Q	Many chromosomes	[238]
	*L. pimpinellifolium*	BC_1_	7 Q	1,2,8,11	[208]
	*L. pimpinellifolium*	BC_2_, BC_3_	8 Q	2,3,4,5,7,9	[286]
	*L. peruvianum*	BC_3_, BC_4_	10 Q	1,2,3,7,8,9,10,12	[240]
	*L. hirsutum*	BC_1_	3 Q	1,3	[235]
	*L. hirsutum*	BC_2_, BC_3_	3 Q	2,3,4	[283]
	*L. pimpinellifolium*	BC_1_S_1_	12 Q	1,2,3,4,6,7,8,9,11,12	[414]
	*L. parviflorum*	BC_3_	8 Q	2,3,6,7,10,11,12	[234]
	*L. esculenum*	F_2_	2 Q	4,6	[417]
	*L. pimpinellifolium*	F_2_	6 Q	1,2,3,11	[40]
	*L. esculentum*	RIL	5 Q	2,3,11,12	[410]
	*L. pimpinellifolium*	BC_2_F_6_	2 Q	2,3	[231]
	*L. pimpinellifolium*	F_2_	7 Q	1,2,3,5,6,7,11	[427]
	*L. pennellii*	BC_2_/BC_2_F_1_	3 Q	3,10,12	[239]
	*L. pennellii*	ILs	13 Q	2,3,4,5,6,7,9,10,11,12	[297]

Frt. yield (total yield)	*L. chmielewskii*	BILs/BC_2_F_5_	1 Q	7	[101]
	*L. pennellii*	ILs	11 Q	Various chromosomes	[238]
	*L. pimpinellifolium*	BC_2_, BC_3_	6 Q	2,3,7,9	[286]
	*L. peruvianum*	BC_3_, BC_4_	10 Q	1,2,6,7,8,9,10,12	[240]
	*L. hirsutum*	BC_2_, BC_3_	12 Q	1,2,3,4,5,6,7,8,12	[283]
	*L. hirsutum*	subNILs	1 Q	1	[405]
	*L. parviflorum*	BC_3_	5 Q	1,2,3,6,8	[234]
	*L. pennellii*	BC_2_/BC_2_F_1_	6 Q	3,5,8,9,12	[239]

Frt. yield (red yield)	*L. pimpinellifolium*	BC_2_, BC_3_	2 Q	2,7	[286]
	*L. peruvianum*	BC_3_, BC_4_	12 Q	1,2,3,5,7,8,9,10,12	[240]
	*L. hirsutum*	BC_2_, BC_3_	11 Q	1,2,3,5,7,8,10,11,12	[283]
	*L. parviflorum*	BC_3_	4 Q	2,5,8	[234]
	*L. pennellii*	BC_2_/BC_2_F_1_	4 Q	3,5,12	[239]

Frt. yield (green yield)	*L. hirsutum*	BC_2_, BC_3_	11 Q	2,3,7,8,11,12	[283]
	*L. pennellii*	BC_2_/BC_2_F_1_	3 Q	8,9,12	[239]

Jointless	*L. cheesmanii*	F_2_	*j*	11	[176, 431]
	*L. cheesmanii*	F_2_	*j-2*	12	[432]

Phytochrome genes	*L. esculentum L. pennellii*	BC_1_	*5 G (PhyA, PhyB1, PhyB2, PhyE, PhyF)*	1, 2, 5, 7, 10	[121]

^(1)^D = distance; Flwr. = flower; Frt. = fruit; L = length; W = width.

**Table 5:** Summary of other characteristics for which genes or QTLs have been identified and mapped in tomato chromosomes.

Trait^(1)^	Wild species used	Mapping population	Genes (G) or QTLs (Q)	Chromosome	References
Branch number	*L. cheesmanii*	F_8_ RIL	7 Q	2,3,4,5,7,11	[280]

Bud type	*L. hirsutum*	BC_1_	7 Q	1,2,7,12	[235]

Curly leaf	*L. pimpinellifolium*	BC_2_F_6_	7 Q	2,3,5,6,8,9,11	[231]

Days to emergence	*L. pimpinellifolium*	BC_1_	3 Q	1,2,3	[208]

Days to 1st flower	*L. pimpinellifolium*	BC_1_	2 Q	1,2	[208]
	*L. pimpinellifolium*	BC_2_F_6_	2 Q	3,4	[231]

Days to 1st ripe fruit	*L. pimpinellifolium*	BC_1_	2 Q	2,4	[208]
	*L. pimpinellifolium*	BC_2_F_6_	2 Q	1,7	[231]

Days to 3rd leaf	*L. pimpinellifolium*	BC_1_	3 Q	1,2,3	[208]

Flwr., number/plant	*L. hirsutum*	BC_1_	1 Q	1	[235]

Flwr., number/truss	*L. pimpinellifolium*	BC_1_	3 Q	3,6,9	[208]
	*L. pimpinellifolium*	BC_2_F_6_	5 Q	1,7,9,10,11	[231]
	*L. pimpinellifolium*	F_2_	9 Q	1,2,3,4,5,7,9	[426]

Flwr. node number	*L. cheesmanii*	F_8_ RILs	5 Q	4,8,9,11	[280]

Hort. acceptability	*L. parviflorum*	BC_3_	3	1,5,9	[234]

Inflores. raquis length	*L. hirsutum*	BC_1_	3 Q	1,5,7	[235]

Inflores. veg. meristem	*L. hirsutum*	BC_1_	2 Q	4,12	[235]

Leaf, length	*L. cheesmanii*	F_8_ RIL	5 Q	2,3,4,6,11	[280]

Leaflet, apex angle	*L. pennellii*	F_2_	4 Q	4,5,7	[404]

Leaflet, D/L ratio	*L. pennellii*	F_2_	1 Q	5	[404]

Leaflet, length	*L. pennellii*	F_2_	4 Q	2,11,12	[404]

Leaflet, number	*L. pennellii*	F_2_	2 Q	1,5	[404]

Leaflet, surface area	*L. pennellii*	F_2_	3 Q	10,11	[404]

Leaflet, width	*L. pennellii*	F_2_	2 Q	17,12	[404]

Leaflet, W/L ratio	*L. pennellii*	F_2_	2 Q	1,4	[404]

Male sterility	*L. pimpinellifolium*	F_2_	ms-10	2	[271]

Node number	*L. cheesmanii*	F_8_ RIL	6 Q	1,2,3,6,8	[280]

Plant cover	*L. peruvianum*	BC_3_, BC_4_	8 Q	1,2,3,5,8,9,10	[240]
	*L. hirsutum*	BC_2_, BC_3_	6 Q	2,3,6,7,8	[283]
	*L. parviflorum*	BC_3_	19	1,2,3,4,5,6,8,9,10,11,12	[234]

Plant fresh mass	*L. cheesmanii*	F_8_ RIL	8 Q	2,3,4,6,9,11	[280]

Plant growth habit	*L. peruvianum*	BC_3_, BC_4_	6 Q	1,2,3,7,8,9	[240]
	*L. pimpinellifolium*	BC_2_F_6_	4 Q	2,3,9,11	[231]

Plant height	*L. pennellii*	ILs	16 Q	Many chromosomes	[238]
	*L. pimpinellifolium*	BC_1_	1 Q	2	[208]
	*L. hirsutum*	BC_1_	4 Q	1,2,5,11	[235]
	*L. cheesmanii*	F_8_ RIL	7 Q	2,3,4,6,7	[280]

Seed number	*L. pimpinellifolium*	BC_1_	4 Q	4,6,7,12	[208]
	*L. pimpinellifolium*	F_2_	2 Q	1,11	[40]
	*L. pimpinellifolium*	BC_2_F_6_	4 Q	5,6,8	[231]
	*L. pimpinellifolium*	F_2_	10 Q	1,2,3,4,6,7,9,11,12	[426]

Seed weight	*L. pennellii*	BC_1_	5 Q	1,2,4,7,8	[274]
	*L. cheesmanii*	F_8_ RILs	14 Q	1,2,3,4,6,7,9,11,12	[279]
	*L. pimpinellifolium*	BC_1_	4 Q	2,4,10,12	[208]
	*L. pimpinellifolium*	F_2_	4 Q	1,2,4	[40]
	*L. pimpinellifolium*	BC_2_F_6_	5 Q	1,4,5,7	[231]

Self incompatibility	*L. peruvianum*	Reciprcal F_1_s	*S* locus	1	[272]
	*L. peruvianum*	F_1_	*S* locus	1	[433]
	*L. hirsutum*	BC_1_	*S* locus	1	[235]

Self pruning	*L. esculentum*	F_2_	*sp*	6	[407]
	*L. chmielewskii*	BC_1_	*sp*	6	[167]
	*L. pimpinellifolium*	BC_1_	*sp*	6	[166]

Stem vascular morphology	*L. hirsutum*	BC_2_S_5_	1 Q	2	[434]

Transgressive Segregation	*L. pennellii*	F_2_	74 Q for 8 traits	All 12 chromosomes	[237]

Unilateral incongruity	*L. hirsutum*	BC_1_	6 Q	1,2,3,11,12	[235]

Various frt. and plant characteristics	*L. pimpinellifolium*	F_2_	Many Q	Undetermined	[276]

^(1)^D = distance; Flwr. = flower; Frt. = fruit; L = length; W = width.

**Table 6:** Known traits for which marker-assisted selection and breeding are done in tomato.

Trait	Source species	Gene/QTL (Q)	Reference
Bacterial canker	*L. peruvianum*	3 Q	Seed companies
	*L. hirsutum*	*Rcm2.0, Rcm5.1*(Q)	[287]

Bacterial speck	*L. pimpinellifolium*	*Pto*	Seed companies, [447]

Bacterial spot	*L. esculentum*	*Rx-3* (Q)	[447]

Bacterial wilt	*L. esculentum*	2 Q	Seed companies

Blackmold	*L. cheesmanii*	Few Q	[288]

Corky root rot	*L. peruvianum*	*Py-1*	Seed companies

Fusarium wilt	*L. pimpinellifolium*	*I-2C, I-3*	Seed companies, public breeders

Jointless	*L. cheesmanii*		Seed companies

Late blight	*L. pimpinellifolium*	*Ph-3*	Seed companies, public breeders
	*L. hirsutum*	4 Q	[342]

Lycopene	*L. esculentum*	*Og^c^, cr*	Seed companies

Powdery mildew	*L. chilense, L. hirsutum*	*Lv, Ol-1, Ol-2*	Seed companies

Ripening inhibitor	*L. cheesmanii*	*rin*	Seed companies

Root-knot nematode	*L. peruvianum*	*Mi*	Seed companies, public breeders

Self pruning	*L. esculentum*	*sp*	Seed companies, public breeders

Soluble solids	Not known	*Q*	Seed companies

Tomato spotted wild virus	*L. peruvianum*	*Sw-5*	Seed companies

Tomato yellow leaf curl virus	Different species	Few Q	Seed companies

Tobacco mosaic virus	*L. peruvianum*	*Tm-2^a^*	Seed companies

Verticillium wilt	*L. esculentum*	*Ve*	Seed companies, public breeders

**Table 7:** Fine-mapped and/or cloned genes and QTLs in tomato.

Trait	Gene/QTL	Chromosome	Source species	Fine mapping population	Nature/activity/ function	Reference
Aphid (potato) resistance	*Meu-1*	6	*L. peruvianum*	NIL F_2_	NBS-LRR	[38, 388, 389]

Bacterial speck resistance	*Pto*	5	*L. pimpinellifolium*	NIL F_2_	Serine-threonine protein kinase	[56, 466, 467]

Brix (soluble solids)	*Brix9-2-5, Lin5*(Q)	9	*L. pennellii*	NIL F_2_	Apoplastic invertase	[98, 302, 303]

Fenthion resistance	*Prf*	5	*L. pimpinellifolium*	NIL F_2_	NBS-LRR Resistance gene	[317, 468]

Flower, exerted stigma	*se2.1* (Q)	2	*L. pennellii*	NIL F_2_ and F_3_	Affects several aspects of floral morphology	[307]

*Frt. color ( *β*-carotene)*	*B*	6	*L. pennellii*	NIL F_2_	Lycopene *β*-cyclase	[131, 299]

*Frt. color (crimson)*	*og^c^, cr*	6	*L. esculentum*	NIL F_2_	Lycopene cyclase null allele	[131, 299]

*Frt. color (high-pigment-2)*	*hp-2*	1	*L. esculentum*	N/A	Homologue of deetiolated 1	[413]

*Frt. color (old gold)*	*og*	6	*L. esculentum*	NIL F_2_	Lycopene cyclase null allele	[131]

*Frt. color (tangerine)*	*CRISTO*	10	*L. esculentum*	NIL F_2_	Carotenoid isomerase	[301]

Frt. ripening (*colorless nonripening*)	*Cnr*	2	*L. esculentum, L. cheesmani*	F_2_ and FISH	ND	[420]

Fruit ripening (*never ripe*)	*Nr*	9	*L. esculentum*	NIL F_2_	Blocks ethylene perception	[421–423]

Fruit ripening (*nonripening*)	*nor*	10	*L. esculentum*	NIL F_2_	MADS-box transcription factor	[153, 424, 425]

Frt. ripening (polygalacturonase)	TOM6	10	*L. esculentum*	N/A	Pectin hydrolyzing	[157, 418, 469]

Fruit ripening (*ripening inhibitor*)	*rin*	5	*L. esculentum L. cheesmanii L. pennellii*	NIL F_2_	MADS-box transcription factor	[153, 424, 425]

Fruit shape	*fs8.1* (Q)	8	*L. pimpinellifolium*	NIL F_2_	Imparts blocky, elongated shape	[438, 470]
	*Sun* (Q)	7	*L. esculentum*	NIL F_2_ and F_3_	Imparts oval shape	[306, 470, 471]
	*ovate* (Q)	2	*L. pimpinellifolium*	NIL F_2_	Plant-growth suppressor	[428, 439, 470]

Fruit weight	*fw2.2* (Q)	2	*L. pennellii*	NIL F_2_	Controls carpel cell number	[305, 435, 470, 472–474]

Fusarium wilt resistance	*I2*	11	*L. esculentum*	NIL F_2_	Leucine zipper and LRR-NBS	[335]

Growth habit	*PW9-2-5 (Q)*	9	*L. pennellii*	F_2_	Semi-determinate growth	[303]

Iron uptake	*chloronerva*	1	*L. pennellii*	NIL F_2_	Nicotianamine synthase	[475]

Jointless	*j*	11	*L. esculentum*	F_2_, NIL F_2_	Suppress formation of pedicel abscission zone	[176, 431, 476, 477]
	*j-2*	12	*L. cheesmanii*	F_2_	Suppress formation of pedicel abscission zone	[432, 478]

Leaf mould resistance	*Cf-2, Cf-4, Cf-5, Cf-9*	1,6	*L. peruvianum*	NIL F_2_	LRR	[479]

Nematode (rootknot) resistance	*Mi-1.1, Mi-1.2*	6	*L. peruvianum*	NIL F_2_	NBS-LRR	[38, 312, 388, 389]

Self pruning (*sp*)	*sp*	6	*L. esculentum*	NIL F_2_	Regulate cycle of vegetative and reproductive growth	[170]

*sp* gene family	*sp21, sp3D, sp5G, sp6A, sp9D*	2,3,5,6,9	*L. pennellii*	NIL F_2_	Not determined	[169]

Self incompatibility	*S*	1	*L. peruvianum*	N/A	RNase activity	[480]

Tomato spotted wilt virus	*Sw-5*	9	*L. peruvianum*	NIL F_2_	NBS-LRR Resistance gene	[481–483]

## References

[1] Willcox JK, Catignani GL, Lazarus S (2003). Tomatoes and cardiovascular health. *Critical Reviews in Food Science and Nutrition*.

[2] Rick CM (1980). Tomato. *Hybridization of Crop Plants*.

[3] Vinson JA, Hao Y, Su X, Zubik L (1998). Phenol antioxidant quantity and quality in foods: vegetables. *Journal of Agricultural and Food Chemistry*.

[4] Nguyen ML, Schwartz SJ (1999). Lycopene: chemical chemical and biological properties. *Food Technology*.

[5] Giovannucci E (1999). Tomatoes, tomato-based products, lycopene, and cancer: review of the epidemiologic literature. *Journal of the National Cancer Institute*.

[6] Knapp S, Bohs L, Nee M, Spooner DM (2004). Solanaceae—a model for linking genomics with biodiversity. *Comparative and Functional Genomics*.

[7] Nee M, Hawkes JG, Lester RN, Estrada N (1991). *Solanaceae III: Taxonomy, Chemistry, Evolution*.

[8] van der Hoeven RS, Ronning C, Giovannoni JJ, Martin G, Tanksley SD (2002). Deductions about the number, organization, and evolution of genes in the tomato genome based on analysis of a large expressed sequence tag collection and selective genomic sequencing. *The Plant Cell*.

[9] Rick CM (1978). The tomato. *Science American*.

[10] Linnaeus C (1753). *Species Planatarium*.

[11] Miller P (1754). *The Gardeners Dictionary*.

[12] Rick CM, Hawkes JG, Lester RN, Skelding AD (1979). Biosystematic studies in *Lycopersicon* and closely related species of *Solanum*. *The Biology and Taxonomy of Solanaceae*.

[13] Rick CM, DeVerna JW, Chetelat RT, Stevens MA (1986). Meiosis in sesquidiploid hybrids of *Lycopersicon esculentum* and *Solanum lycopersicoides*. *Proceedings of the National Academy of Sciences of the United States of America*.

[14] Warnock SJ (1988). A review of taxonomy and phylogeny of the genus *Lycopersicon*. *HortScience*.

[15] Olmstead RG, Sweere JA, Spangler RE, Bohs L, Palmer J, Nee M, Symon DE, Lester RN, Jesssop JP (1999). Phylogeny and provisional classification of the Solanaceae based
on chloroplast DNA. *Solanaceae IV*.

[16] Spooner DM, Anderson GJ, Jansen RK (1993). Chloroplast DNA evidence for the interrelationships of tomtoes, potatoes, and pepinos (Solanaceae). *American Journal of Botany*.

[17] Terrell EE, Broome CR, Reveal JL (1983). Proposal to conserve the name of the tomato as *Lycopersicon esculentum* P. Miller and reject the combination *Lycopersicon lycopersicum* (L.) Karsten (Solanaceae). *Taxon*.

[18] Peralta IE, Spooner DM (2001). Granule-bound starch synthesis (GBSSI) gene phylogeny of wild tomatoes (*Solanum* L. section *Lycopersicon* [Mill.] Wettst. subsection *Lycopersicon*). *American Journal of Botany*.

[19] Marshall JA, Knapp S, Davey MR (2001). Molecular systematics of *Solanum* section *Lycopersicum* (*Lycopersicon*) using the nuclear ITS rDNA region. *Theoretical and Applied Genetics*.

[20] Peralta IE, Knapp S, Spooner DM (2005). New species of wild tomatoes (*Solanum* section *Lycopersicon*: Solanaceae) from Northern Peru. *Systematic Botany*.

[21] Spooner DM, Peralta IE, Knapp S (2005). Comparison of AFLPs with other markers for phylogenetic inference in wild tomatoes [*Solanum* L. section *Lycopersicon* (Mill.) Wettst.]. *Taxon*.

[22] Miller P (1768). *The Gardeners Dictionary*.

[23] Rick CM (1976). Natural variability in wild species of *Lycopersicon* and its bearing on tomato breeding. *Genet Agraria*.

[24] Rick CM Potential improvement of tomatoes by controlled introgression of genes from wild speies.

[25] Rick CM, Simmonds NW (1976). Tomato, *Lycopersicon esculentum* (Solanaceae). *Evolution of Crop Plants*.

[26] Rick CM, Nevins DJ, Jones RA (1987). Genetic resources in *Lycopersicon*. *Tomato Biotechnology*.

[27] Rick CM, Vasil IK, Scowcroft WR, Frey KJ (1982). The potential of exotic germplasm for tomato improvement. *Plant Improvement and Somatic Cell Genetics*.

[28] Rick CM, Butler L (1956). Cytogenetics of the tomato. *Advances in Genetics*.

[29] Rick CM, Lamm R (1955). Biosystematics studies on the status of *Lycopersicon chilense*. *American Journal of Botany*.

[30] Stevens MA, Rick CM, Atherton JG, Rudich J (1986). Genetics and breeding. *The Tomato Crop: A Scientific Basis for Improvement*.

[31] Rick CM, DeVerna JW, Chetelat RT, Stevens MA (1987). Potential contributions of wide crosses to improvement of processing tomatoes. *Acta Horticulturae*.

[32] Rick CM, Srb AM (1973). Potential genetic resources in tomato species: clues from observations in native habitats. *Genes, Enzymes, and Populations*.

[33] Scott JW, Olson SM, Howe TK, Stoffella PJ, Bartz JA, Bryan HH (1995). ‘Equinox’ heat-telerant hybrid tomato. *HortScience*.

[34] Tigchelaar EC, Bassett MJ (1986). Tomato breeding. *Breeding for Vegetable Crops*.

[35] Miller JC, Tanksley SD (1990). RFLP analysis of phylogenetic relationships and genetic variation in the genus *Lycopersicon*. *Theoretical and Applied Genetics*.

[36] Rick CM, Fobes JF (1975). Allozyme variation in the cultivated tomato and closely related species. *Bulletin of the Torrey Botanical Club*.

[37] Bretó MP, Asins MJ, Carbonell EA (1993). Genetic variability in *Lycopersicon* species and their genetic relationships. *Theoretical and Applied Genetics*.

[38] Rossi M, Goggin FL, Milligan SB, Kaloshian I, Ullman DE, Williamson VM (1998). The nematode resistance gene *Mi* of tomato confers resistance against the potato aphid. *Proceedings of the National Academy of Sciences of the United States of America*.

[39] Kalloo G (1991). *Genetic Improvement of Tomato*.

[40] Lippman Z, Tanksley SD (2001). Dissecting the genetic pathway to extreme fruit size in tomato using a cross between the small-fruited wild species *Lycopersicon pimpinellifolium* and *L. esculentum* var. Giant Heirloom. *Genetics*.

[41] Jenkins JA (1948). The origin of the cultivated tomato. *Economic Botany*.

[42] Williams JGK, Hanafey MK, Rafalski JA, Tingey SV (1993). Genetic analysis using random amplified polymorphic DNA markers. *Methods in Enzymology*.

[43] Rick CM (1991). Tomato paste: a concentrated review of genetic highlights from the beginnings to the advent of molecular genetics. *Genetics*.

[44] Rick CM, Yoder JI (1988). Classical and molecular genetics of tomato: highlights and perspectives. *Annual Review of Genetics*.

[45] Arumuganathan K, Earle ED (1991). Nuclear DNA content of some important plant species. *Plant Molecular Biology Reporter*.

[46] Peterson DG, Pearson WR, Stack SM (1998). Characterization of the tomato (*Lycopersicon esculentum*) genome using in vitro and in situ DNA reassociation. *Genome*.

[47] McCormick S, Niedermeyer J, Fry J, Barnason A, Horsch R, Fraley R (1986). Leaf disc transformation of cultivated tomato (*L. esculentum*) using *Agrobacterium tumefaciens*. *Plant Cell Reports*.

[48] Fillatti JJ, Kiser J, Rose R, Comai L (1987). Efficient transfer of glyphosate tolerance gene into tomato using binary *Agrobacterium tumefaciens* vector. *Bio/Technology*.

[49] Zagorska NA, Shtereva A, Dimitrov BD, Kruleva MM (1998). Induced anderogenesis in tomato (*Lycopersicon esculentum* Mill.) I. Influence of genotype on androgenetic ability. *Plant Cell Reports*.

[50] Menda N, Semel Y, Peled D, Eshed Y, Zamir D (2004). *In silico* screening of a saturated mutation library of tomato. *Plant Journal*.

[51] Bonnema G, Hontelez J, Verkerk R (1996). An improved method of partially digesting plant megabase DNA suitable for YAC cloning: application to the construction of a 5.5 genome equivalent YAC library of tomato. *The Plant Journal*.

[52] Budiman MA, Mao L, Wood TC, Wing RA (2000). A deep-coverage tomato BAC library and prospects toward development of an STC framework for genome sequencing. *Genome Research*.

[53] Hamilton CM, Frary A, Xu Y, Tanksley SD, Zhang H-B (1999). Construction of tomato genomic DNA libraries in a binary-BAC (BIBAC) vector. *Plant Journal*.

[54] Patil RS, Davery MR, Power JB, Cocking EC (2002). Effective protocols for *Agrobacterium*-mediated leaf disc trasformation in tomato (*Lycopersicon esculentum* Mill.). *Indian Journal of Biotechnology*.

[55] Breuning G, Lyons JM (2000). The case of the FLAVR SAVR tomato. *California Agriculture*.

[56] Martin GB, Brommonschenkel SH, Chunwongse J (1993). Map-based cloning of a protein kinase gene conferring disease resistance in tomato. *Science*.

[57] Martin GB, Vicente MC, Tanksley SD (1993). High-resolution linkage analysis and physical chraracterization of the *Pto* bacterial resistance locus in tomato. *Molecular Plant-Microbiology Interactions*.

[58] Warren GF (1998). Spectacular increases in crop yields in the United States in the twentieth century. *Weed Technology*.

[59] Duvick DN (1986). Plant breeding: past achievements and expectations for the future. *Economic Botany*.

[60] Scott JW (1993). Breeding tomatoes for resistance to high temperatures, biotic and abiotic diseases. *Hort Bras*.

[61] Scott JW, Bryan HH, Ramos LJ (1997). High temperature fruit setting ability of large-fruited, jointless pedicel tomato hybrids with various combinations of heat-tolerance. *Proceedings of the Florida State Horticultural Society*.

[62] Lukyanenko AN, Kalloo G (1991). Disease resistance in tomato. *Genetic Improvement of Tomato*.

[63] Scott JW, Francis DM, Miller SA, Somodi GC, Jones JB (2003). Tomato bacterial spot resistance derived from PI 114490; inheritance of resistance to race T2 and relationship across three pathogen races. *Journal of the American Society for Horticultural Science*.

[64] Scott JW, Jones JB (1986). Sources of resistance to bacterial spot in tomato. *HortScience*.

[65] Scott JW, Jones JB, Somodi GC (1995). Screening tomato accessions for resistance to *Xanthomonas comapestris* pv. *vesicatoria*, race T3. *HortScience*.

[66] Scott JW, Miller SA, Stall RE (1997). Resistance to race T2 of the bacterial spot pathogen in tomato. *HortScience*.

[67] Stommel JR, Zhang YP (1998). Molecular markers linked to quantitative trait loci for anthracnose resistance in tomato (Abstract). *HortScience*.

[68] Yang W, Sacks EJ, Lewis Ivey ML, Miller SA, Francis DM (2005). Resistance in *Lycopersicon esculentum* intraspecific crosses to race T1 strains of *Xanthomonas campestris* pv. *vesicatoria* causing bacterial spot of tomato. *Phytopathology*.

[69] Yu ZH, Wang JF, Stall RE, Vallejos CE (1995). Genomic localization of tomato genes that control a hypersensitive reaction to *Xanthomonas campestris* pv. *vesicatoria* (Doidge) dye. *Genetics*.

[70] Farrar RRJ, Barbour JD, Kennedy GG (1994). Field evaluation of insect resistance in a wild tomato and its effects on insect parasitoids. *Entomologia Experimentalis et Applicata*.

[71] Juvik JA, Berlinger MJ, Ben-David T, Rudich J (1982). Resistance among accessions of the genera *Lycopersicon*
and *Solanum* to four of the main insect pests of tomato in Israel. *Phytoparasitica*.

[72] Muigai SG, Bassett MJ, Schuster DJ, Scott JW (2003). Greenhouse and field screening of wild *Lycopersicon* germplasm for resistance to the whitefly *Bemisia argentifolii*. *Phytoparasitica*.

[73] Mutschler MA, Doerge RW, Liu S-C, Kuai JP, Liedl BE, Shapiro JA (1996). QTL analysis of pest resistance in the wild tomato *Lycopersicon pennellii*: QTLs controlling acylsugar level and composition. *Theoretical and Applied Genetics*.

[74] Schalk JM, Stoner AK (1976). A bioassay differentiates resistance to the Colorado potato beetle on tomatoes. *Journal of the American Society for Horticultural Science*.

[75] Weston PA, Johnson DA, Burton HT, Snyder JC (1989). Trichome secretion composition, trichome densities, and spider mite resistance of ten accessions of *Lycopersicon hirsutum*. *Journal of the American Society for Horticultural Science*.

[76] Farrar RRJ, Kennedy GG, Kalloo G (1991). Insect and mite resistance in tomato. *Genetic Improvement of Tomato*.

[77] Goffreda JC, Mutschler MM (1989). Inheritance of potato aphid resistance in hybrids
between *Lycopersicon esculentum* and *L. pennellii*. *Theoretical and Applied Genetics*.

[78] Juvik JA, Stevens MA (1982). Inheritance of foliar 
*α*-tomatine content in tomatoes. *Journal of the American Society for Horticultural Science*.

[79] Hartman JB, St Clair DA (1998). Variation for insect resistance and horticultural traits in tomato inbred backcross populations 
derived from *Lycopersicon pennellii*. *Crop Science*.

[80] Kohler GR, St Clair DA (2005). Variation for resistance to aphids (Homoptera: Aphididae) among tomato inbred backcross lines derived from wild *Lycopersicon* species. *Journal of Economic Entomology*.

[81] Foolad MR, Ashraf M, Harris PJC (2005). Breeding for abiotic stress tolerances in tomato. *Abiotic Stresses: Plant Resistance Through Breeding and Molecular Approaches*.

[82] Rick CM (1988). Tomato-like nightshades: affinities, autoecology and breeders' opportunities. *Economic Botany*.

[83] Rick CM, DeVerna JW, Chetelat RT, Bennett AB, O'Neill SD Experimental introgression to the cultivated tomato from related wild nightshades.

[84] Dehan K, Tal M (1978). Salt tolerance in the wild relatives of the cultivated tomato: responses of *Solanum pennellii* to high salinity. *Irrigation Science*.

[85] Foolad MR, Lin GY (1998). Genetic analysis of low-temperature tolerance during germination in tomato, *Lycopersicon esculentum* Mill. *Plant Breeding*.

[86] Foolad MR, Zhang LP, Subbiah P (2003). Genetics of drought tolerance during seed germination in tomato: inheritance and QTL mapping. *Genome*.

[87] Kalloo G, Kalloo G (1991). Breeding for environmental resistance in tomato. *Genetic Improvement of Tomato*.

[88] Martin B, Tauer CG, Lin RK (1999). Carbon isotope discrimination as a tool to improve water-use efficiency in tomato. *Crop Science*.

[89] Patterson BD, Payne LA (1983). Screening for chilling resistance in tomato seedlings. *HortScience*.

[90] Scott JW, Volin RB, Bryan HH, Olson SM (1986). Use of hybrids to develop heat tolerant tomato cultivars. *Proceedings of the Florida State Horticultural Society*.

[91] Villareal RL, Lai SH Development of heat-tolerant tomato varieties in the tropics.

[92] Asins MJ, Bretó MP, Cambra M, Carbonell EA (1993). Salt tolerance in *Lycopersicon* species. I. Character definition and changes in gene expression. *Theoretical and Applied Genetics*.

[93] Foolad MR (1999). Comparison of salt tolerance during seed germination and vegetative growth in tomato by QTL mapping. *Genome*.

[94] Foolad MR (1999). Genetics of salt tolerance and cold tolerance in tomato: quantitative analysis and QTL mapping. *Plant Biotechnology*.

[95] Foolad MR, Chen FQ, Lin GY (1998). RFLP mapping of QTLs conferring salt tolerance during germination in an interspecific cross of tomato. *Theoretical and Applied Genetics*.

[96] Foolad MR, Lin GY (1997). Absence of a relationship
between salt tolerance during germination and vegetative growth in tomato. *Plant Breeding*.

[97] Jones RA, Qualset CO, Collins GB, Petolino JF (1984). Breeding crops for environmental stress tolerance. *Application of Genetic Engineering to Crop Improvement*.

[98] Fridman E, Pleban T, Zamir D (2000). A recombination hotspot delimits a wild-species quantitative trait locus for tomato sugar content to 484 bp within an invertase gene. *Proceedings of the National Academy of Sciences of the United States of America*.

[99] Stevens MA (1972). Relationships between components contributing to quality variation among tomato lines. *Journal of the American Society for Horticultural Science*.

[100] Stevens MA (1986). Inheritance of tomato fruit quality components. *Plant Breeding Reviews*.

[101] Azanza F, Young TE, Kim D, Tanksley SD, Juvik JA (1994). Characterization of the effect of introgressed segments of chromosome 7 and 10 from *Lycopersicon chmielewskii* on tomato soluble solids, pH, and yield. *Theoretical and Applied Genetics*.

[102] Davies JN (1966). Occurrence of sucrose in the fruit of some species of *Lycopersicon*. *Nature*.

[103] Chetelat RT, DeVerna JW, Bennett AB (1995). Introgression into tomato (*Lycopersicon esculentum*) of the *Lycopersicon chmielewskii* sucrose accumulator gene (*sucr*) controlling fruit sugar composition. *Theoretical and Applied Genetics*.

[104] Davies JN, Hobson GE (1981). The constituents of tomato fruit—the influence of environment, nutrition, and genotype. *Critical Reviews in Food Science and Nutrition*.

[105] Hewitt JD, Garvey TC, Nevins DJ, Jones RA (1987). Wild sources of high soluble solids in tomato. *Tomato Biotechnology*.

[106] Berry SZ, Uddin MR, Kalloo G (1991). Breeding tomato for quality and processing attributes. *Genetic Improvement of Tomat*.

[107] Wood M (1992). Solid future for tomatoes. *Agricultural Research*.

[108] Jones RA, Scott SJ (1984). Genetic potential to improve tomato flavor
in commercial 
*F* hybrids. *Journal of the American Society for Horticultural Science*.

[109] Stevens MA, Kader AA, Albright M (1979). Potential for increasing tomato flavor via increased sugar and acid content breeding. *Journal of the American Society for Horticultural Science*.

[110] Rick CM (1974). High soluble-solids content in large-fruited tomato lines derived from a wild green-fruited species. *Hilgardia*.

[111] Stoner AK, Thompson AE (1966). The potential for selecting and breeding for solids content of tomatoes. *Proceedings of American Society for Horticultural Science*.

[112] Georgelis N, Scott JW, Baldwin EA (2004). Relationship of tomato fruit sugar concentration with physical and chemical traits and linkage of RAPD markers. *Journal of the American Society for Horticultural Science*.

[113] Stevens MA, Rudich J (1978). Genetic potential for overcoming physiological limitations on adaptability, yield and quality in tomato. *HortScience*.

[114] Gerster H (1997). The potential role of lycopene for human health. *Journal of the American College of Nutrition*.

[115] Giovannucci E, Clinton SK (1998). Tomatoes, lycopene, and prostate cancer. *Proceedings of the Society for Experimental Biology and Medicine*.

[116] Sies H, Stahl W (1998). Lycopene: antioxidant and biological effects and its bioavailability in the human. *Proceedings of the Society for Experimental Biology and Medicine*.

[117] Tsubono Y, Tsugane S, Gey KF (1999). Plasma antioxidant vitamins and carotenoids in five Japanese populations with varied mortality from gastric cancer. *Nutrition and Cancer*.

[118] Di Mascio P, Devasagayam TPA, Kaiser S, Sies H (1990). Carotenoids, tocopherols and thiols as biological singlet molecular oxygen quenchers. *Biochemical Society Transactions*.

[119] Khachik F, Carvalho L, Bernstein PS, Muir GJ, Zhao D-Y, Katz NB (2002). Chemistry, distribution, and metabolism of tomato carotenoids and their impact on human health. *Experimental Biology and Medicine*.

[120] Yen H-C, Shelton BA, Howard LR, Lee S, Vrebalov J, Giovannoni JJ (1997). The tomato *high-pigment (hp)* locus maps to chromosome 2 and influences plastome copy number and fruit quality. *Theoretical and Applied Genetics*.

[121] van Tuinen A, Cordonnier-Pratt M-M, Pratt LH, Verkerk R, Zabel P, Koornneef M (1997). The mapping of phytochrome genes and photomorphogenic mutants of tomato. *Theoretical and Applied Genetics*.

[122] Baker LR, Tomes ML (1964). Carotenoids and chlorophyll in two tomato mutants and their hybrid. *Proceedings of the American Society for Horticultural Science*.

[123] Reynard GB (1956). Origin of the Webb Special (Black Queen) tomato. *Report of the Tomato Genetics Cooperative*.

[124] Soressi GP (1975). New spontaneous or chamically-induced fruit-ripening tomato mutants. *Report of the Tomato Genetics Cooperative*.

[125] Thompson AE, Hepler RW, Kerr EA (1962). Clarification of the inheritance of high total carotenoid pigments in the tomato. *Journal of the American Society for Horticultural Science*.

[126] Palmieri S, Martiniello P, Soressi GP (1978). Chlorophyll and carotene content in *high pigment* and *green flesh* fruits. *Report of the Tomato Genetics Cooperative*.

[127] Jarret RL, Sayama H, Tigchelaar EC (1984). Pleiotropic effects associated with the chlorophyll intesifier mutations high pigment and dark green in tomatoes. *Journal of the American Society of Horticultural Science*.

[128] Kerr EA (1956). High pigment ratios. *Report of the Tomato Genetics Cooperative*.

[129] Wann EV (1995). Reduced plant growth in tomato mutants high pigment and dark green partially overcome by gibberellin. *HortScience*.

[130] Butler L (1962). Crimson, a new fruit colour. *Report of the Tomato Genetics Cooperative*.

[131] Ronen G, Carmel-Goren L, Zamir D, Hirschberg J (2000). An alternative pathway to 
*β*-carotene formation in plant chromoplasts discovered by map-based cloning of *Beta* and *old-gold* color mutations in tomato. *Proceedings of the National Academy of Sciences of the United States of America*.

[132] Thompson AE, Tomes ML, Erickson HT, Wann EV, Armstrong RJ (1967). Inheritance of crimson fruit color in tomatoes. *Proceedings of the American Society for Horticultural Science*.

[133] Thompson AE, Tomes ML, Wann EV, McCollum JP, Stoner AK (1965). Characterization of crimson tomato fruit color. *Proceedings of the American Society for Horticultural Science*.

[134] Rice AC, Pederson CS (1954). Factors influencing growth of *Bacillus coagulans* in canned tomato juice. II. Acidic constituents of tomato juice and specific organic acids. *Food Research*.

[135] Lambeth VN, Straten EF, Fields ML (1966). *Fruit Quality Attributes of 250 Foreign and Domestic Tomato Accessions*.

[136] Baldwin EA, Scott JW, Shewmaker CK, Schuch W (2000). Flavor trivia and tomato aroma: biochemistry and possible mechanisms for control of important aroma components. *HortScience*.

[137] Goff SA, Klee HJ (2006). Plant volatile compounds: sensory cues for health and nutritional value?. *Science*.

[138] Tandon KS, Baldwin EA, Scott JW, Shewfelt RL (2003). Linking sensory descriptors to volatile and nonvolatile components of fresh tomato flavor. *Journal of Food Science*.

[139] Jones RA, Scott SJ (1983). Improvement of tomato flavor by genetically increasing sugar and acid contents. *Euphytica*.

[140] Stevens MA, Kader AA, Albright-Holton M, Algazi M (1977). Genotypic variation for flavor and composition in fresh market tomatoes. *Journal of the American Society for Horticultural Science*.

[141] Levin I, Gilboa N, Yeselson E, Shen S, Schaffer AA (2000). *Fgr*, a major locus that modulates the fructose to glucose ratio in mature tomato fruits. *Theoretical and Applied Genetics*.

[142] Bucheli P, Voirol E, de la Torre R (1999). Definition of nonvolatile markers for flavor of tomato (*Lycopersicon esculentum* Mill.) as tools in selection and breeding. *Journal of Agricultural and Food Chemistry*.

[143] Causse M, Saliba-Colombani V, Lecomte L, Duffé P, Rousselle P, Buret M (2002). QTL analysis of fruit quality in fresh market tomato: a few chromosome regions control the variation of sensory and instrumental traits. *Journal of Experimental Botany*.

[144] Buttery RG, Acree TE, Teranishi R (1993). Quantitative and sensory aspects of flavor of tomato and other vegetables and fruits. *Flavor Science: Sensible Principles and Techniques*.

[145] Scott JW (2002). A breeder's perspective on the use of molecular techniques for improving fruit quality. *HortScience*.

[146] Kader AA, Stevens MA, Albright-Holton M, Morris LL, Algazi M (1977). Effect of fruit ripeness when picked on flavor and composition in fresh market tomatoes. *Journal of the American Society for Horticultural Science*.

[147] Mizrahi Y, Taleisnik E, Kagan-Sur V (1988). A saline irrigation regime for improving tomato fruit quality without reducing yield. *Journal of the American Society for Horticultural Science*.

[148] Niedziela JCE, Nelson PV, Willits DH, Peet MM (1993). Short-term salt-shock effects on tomato fruit quality, yield, and vegetative prodiction of subsequent fruit quality. *Journal of the American Society for Horticultural Science*.

[149] McDonald RE, McCollum TG, Baldwin EA (1996). Prestorage heat treatments influence free sterols and flavor volatiles of tomatoes stored at chilling temperature. *Journal of the American Society for Horticultural Science*.

[150] Bruhn CM, Feldman N, Garlitz C (1991). Consumer perceptions of quality: apricots, cantaloupes, peaches, pears, strawberries, and tomatoes. *Journal of Food Quality*.

[151] Giovannoni JJ (2001). Molecular biology of fruit maturation and ripening. *Annual Review of Plant Physiology and Plant Molecular Biology*.

[152] Giovannoni JJ (2004). Genetic regulation of fruit development and ripening. *The Plant Cell*.

[153] Moore S, Vrebalov J, Payton P, Giovannoni J (2002). Use of genomics tools to isolate key ripening genes and analyse fruit maturation in tomato. *Journal of Experimental Botany*.

[154] Alba R, Payton P, Fei Z (2005). Transcriptome and selected metabolite analyses reveal multiple points of ethylene control during tomato fruit development. *The Plant Cell*.

[155] Giovannoni JJ, Yen H, Shelton B (1999). Genetic mapping of ripening and ethylene-related loci in tomato. *Theoretical and Applied Genetics*.

[156] Brady CJ, MacAlpine G, McGlasson WB, Veda Y (1982). Polygalacturonase in tomato fruits and the induction of ripening. *Australian Journal of Plant Physiology*.

[157] Dellapenna D, Alexandert DC, Bennett AB (1986). Molecular cloning of tomato fruit polygalacturonase: analysis of polygalacturonase mRNA levels during ripening. *Proceedings of the National Academy of Sciences of the United States of America*.

[158] Tigchelaar EC, McGlasson W, Buescher R (1978). Genetic regulation of tomato fruit ripening. *HortScience*.

[159] Mutschler MA (1984). Inheritance and linkage of the Alcobaca ripening mutant in tomato. *Journal of the American Society for Horticultural Science*.

[160] Kinzer SM, Schwager SJ, Mutschler MA (1990). Mapping of ripening-related or -specific cDNA clones of tomato (*Lycopersicon esculentum*). *Theoretical and Applied Genetics*.

[161] Kopeliovitch E, Mizrahi Y, Rabinowitch HD, kedar N (1979). Effect of the fruit-ripeniing mutant genes *rin* and *nor* on the flavor of tomato fruit. *Journal of the American Society for Horticultural Science*.

[162] Odland ML, Greech RG, McArdle FG (1968). *Evaluation of Tomato Varieties for Mechanized Harvest Potential*.

[163] Sims WL, Zobel MB (1968). *Mechanized Growing and Harvesting of Processing Tomatoes*.

[164] Yeager AF (1927). Determinate growth in tomato. *Journal of Heredity*.

[165] MacArthur JW (1932). Inherited characters in tomato. I. The self pruning habit. *Journal of Heredity*.

[166] Grandillo S, Tanksley SD (1996). Genetic analysis of RFLPs, GATA microsatellites and RAPDs in a cross between *L. esculentum* and *L. pimpinellifolium*. *Theoretical and Applied Genetics*.

[167] Paterson AH, Lander ES, Hewitt JD, Peterson S, Lincoln SE, Tanksley SD (1988). Resolution of quantitative traits into Mendelian factors by using a complete linkage map of restriction fragment length polymorphisms. *Nature*.

[168] Ozminkowski JRH, Gardener RG, Henderson WR, Moll RH (1990). Prostrate growth habit enhances fresh market tomato fruit yield
and quality. *HortScience*.

[169] Carmel-Goren L, Liu YS, Lifschitz E, Zamir D (2003). The *SELF-PRUNING* gene family in tomato. *Plant Molecular Biology*.

[170] Pnueli L, Carmel-Goren L, Hareven D (1998). The *SELF-PRUNING* gene of tomato regulates vegetative to reproductive switching of sympodial meristems and is the ortholog of *CEN* and *TFL1*. *Development*.

[171] Georgiev H, Kalloo G (1991). Heterosis in tomato breeding. *Genetic Improvement of Tomato*.

[172] Scott JW, Angell FF, Banga SS, Banga SK (1998). Tomato. *Hybrid Cultivar Development*.

[173] Duvick DN (1996). Personal perspective plant breeding, an evolutionary concept. *Crop Science*.

[174] Tanksley SD (1993). Mapping polygenes. *Annual Review of Genetics*.

[175] Paterson AH, Tanksley SD, Sorrells ME (1991). DNA markers in plant improvement. *Advances in Agronomy*.

[176] Zhang H-B, Martin GB, Tanksley SD, Wing RA (1994). Map based cloning in crop plants: tomato as a model system II. Isolation and characterization of a set of ovevlapping yeast artificial chromosomes encompassing the *jointless* locus. *Molecular and General Genetics*.

[177] Tanksley SD, McCouch SR (1997). Seed banks and molecular maps: unlocking genetic potential from the wild. *Science*.

[178] Sax K (1923). The association of size differences with seed coat pattern and pigmentation in *Phaesolus vulgaris*. *Genetics*.

[179] Chetelat RT (2002). Revised list of monogenic stocks. *Report of the Tomato Genetics Cooperative*.

[180] Tanksley SD, O'Brian SJ (1993). Linkage map of the tomato (*Lycopersicon esculentum*) (2N = 24). *Genetic Maps: Locus Maps of Complex Genomes*.

[181] Mutschler MA, Tanksley SD, Rick CM (1987). Linkage maps of the tomato (*Lycopersicon esculentum*). *Report of the Tomato Genetics Cooperative*.

[182] Tanksley SD, Bernatzky R, Nevins DJ, Jones RA (1987). Molecular markers for the nuclear genome of tomato. *Plant Biology, Vol 4, Tomato Biotechnology*.

[183] Foolad MR, Jones RA, Rodriguez RL (1993). RAPD markers for
constructing intraspecific tomato genetic maps. *Plant Cell Reports*.

[184] Tanksley SD, Orton TJ (1983). *Isozymes in Plant Genetics and Breeding*.

[185] Butler L (1968). Linkage summary. *Report of the Tomato Genetics Cooperative*.

[186] Rick CM, King RC (1975). The tomato. *Handbook of Genetics, Vol. 2*.

[187] Tanksley SD, Rick CM (1980). Isozymic gene linkage map of the tomato: applications in genetics and breeding. *Theoretical and Applied Genetics*.

[188] Botstein D, White RL, Skolnick M, Davis RW (1980). Construction of a genetic linkage map in man using restriction fragment length polymorphisms. *American Journal of Human Genetics*.

[189] Williams JGK, Kubelik AR, Livak KJ, Rafalski JA, Tingey SV (1990). DNA polymorphisms amplified by arbitrary primers are useful as genetic markers. *Nucleic Acids Research*.

[190] Vos P, Hogers R, Bleeker M (1995). AFLP: a new technique for DNA fingerprinting. *Nucleic Acids Research*.

[191] Jeffreys AJ, Wilson V, Thein SL (1985). Hypervariable ‘minisatellite’ regions in human DNA. *Nature*.

[192] He C, Poysa V, Yu K (2003). Development and characterization of simple sequence repeat (SSR) markers and their use in determining relationships among *Lycopersicon esculentum* cultivars. *Theoretical and Applied Genetics*.

[193] Tautz D (1989). Hypervariability of simple sequences as a general source for polymorphic DNA markers. *Nucleic Acids Research*.

[194] Konieczny A, Ausubel FM (1993). A procedure for mapping *Arabidopsis* mutations using co-dominant ecotype-specific PCR-based markers. *Plant Journal*.

[195] Paran I, Michelmore RW (1993). Development of reliable PCR-based markers linked to downy mildew resistance genes in lettuce. *Theoretical and Applied Genetics*.

[196] Orita M, Iwahana H, Kanazawa H, Hayashi K, Sekiya T (1989). Detection of polymorphisms of human DNA by gel electrophoresis as single-strand conformation polymorphisms. *Proceedings of the National Academy of Sciences of the United States of America*.

[197] Adams MD, Kelley JM, Gocayne JD (1991). Complementary DNA sequencing: expressed sequence tags and human genome project. *Science*.

[198] Fulton TM, van der Hoeven R, Eannetta NT, Tanksley SD (2002). Identification, analysis, and utilization of conserved ortholog set markers for comparative genomics in higher plants. *The Plant Cell*.

[199] Landegren U, Nilsson M, Kwok P-Y (1998). Reading bits of genetic information: methods for single-nucleotide polymorphism analysis. *Genome Research*.

[200] Fei Z, Tang X, Alba RM (2004). Comprehensive EST analysis of tomato and comparative genomics of fruit ripening. *Plant Journal*.

[201] Labate JA, Baldo AM (2005). Tomato SNP discovery by EST mining and resequencing. *Molecular Breeding*.

[202] Alvarez AE, van de Wiel CCM, Smulders MJM, Vosman B (2001). Use of microsatellites to evaluate genetic diversity and species relationships in the genus *Lycopersicon*. *Theoretical and Applied Genetics*.

[203] Arens P, Odinot P, van Heusden AW, Lindhout P, Vosman B (1995). GATA- and GACA-repeats are not evenly distributed throughout the tomato genome. *Genome*.

[204] Areshchenkova T, Ganal MW (1999). Long tomato microsatellites are predominantly associated with centromeric regions. *Genome*.

[205] Areshchenkova T, Ganal MW (2002). Comparative analysis of polymorphism and chromosomal location of tomato microsatellite markers isolated from different sources. *Theoretical and Applied Genetics*.

[206] Broun P, Tanksley SD (1996). Characterization and genetic mapping of simple repeat sequences in the tomato genome. *Molecular and General Genetics*.

[207] Frary A, Xu Y, Liu J, Mitchell S, Tedeschi E, Tanksley SD (2005). Development of a set of PCR-based anchor markers encompassing the tomato genome and evaluation of their usefulness for genetics and breeding experiments. *Theoretical and Applied Genetics*.

[208] Grandillo S, Tanksley SD (1996). QTL analysis of horticultural traits differentiating the cultivated tomato from the closely related
species *Lycopersicon pimpinellifolium*. *Theoretical and Applied Genetics*.

[209] Smulders MJM, Bredemeijer G, Rus-Kortekaas W, Arens P, Vosman B (1997). Use of short microsatellites from database sequences to generate polymorphisms among *Lycopersicon esculentum* cultivars and accessions of other *Lycopersicon* species. *Theoretical and Applied Genetics*.

[210] Suliman-Pollatschek S, Kashkush K, Shats H, Hillel J, Lavi U (2002). Generation and mapping of AFLP, SSRs and SNPs in *Lycopersicon esculentum*. *Cellular and Molecular Biology Letters*.

[211] Villalta I, Reina-Sånchez A, Cuartero J, Carbonell EA, Asins MJ (2005). Comparative microsatellite linkage analysis and genetic structure of two populations of 
*F* lines derived from *Lycopersicon pimpinellifolium* and *L. cheesmanii*. *Theoretical and Applied Genetics*.

[212] Vosman B, Arens P, Rus-Kortekaas W, Smulders MJM (1992). Identification of highly polymorphic DNA regions in tomato. *Theoretical and Applied Genetics*.

[213] Fulton TM, Xu Y, Siew FL, Tanksley SD (1999). Efficiency of using CAPS as an alternative and potentially automatable
mapping seystem. *Report of the Tomato Genetics Cooperative*.

[214] Ganal MW, Czihal R, Hannappel U, Kloos D-U, Polley A, Ling H-Q (1998). Sequencing of cDNA clones from the genetic map of tomato (*Lycopersicon esculentum*). *Genome Research*.

[215] Saliba-Colombani V, Causse M, Gervais L, Philouze J (2000). Efficiency of RFLP, RAPD, and AFLP markers for the construction of an intraspecific map of the tomato genome. *Genome*.

[216] Niño-Liu DO, Zhang LP, Foolad MR (2003). Sequence comparison and characterization of DNA fragments amplified by resistance gene primers in tomato. *ISHS Acta Horticulturae*.

[217] Zhang LP, Khan A, Niño-Liu D, Foolad MR (2002). A molecular linkage map of tomato displaying chromosomal locations of resistance gene analogs based on a *Lycopersicon esculentum*
×
*L. hirsutum* cross. *Genome*.

[218] Haanstra JPW, Wye C, Verbakel H (1999). An integrated high-density RFLP-AFLP map of tomato based on two *Lycopersicon esculentum*
×
*L. pennellii*
*F* populations. *Theoretical and Applied Genetics*.

[219] Huang CC, Cui Y-Y, Weng CR, Zabel P, Lindhout P (2000). Development of diagnostic PCR markers closely linked to the tomato powdery mildew resistance gene *Ol-1* on chromosome 6 of tomato. *Theoretical and Applied Genetics*.

[220] Zhang Y, Stommel JR (2001). Development of SCAR and CAPS markers linked to the Betas gene in tomato. *Crop Science*.

[221] Ruiz JJ, García-Martínez S, Picó B, Gao M, Quiros CF (2005). Genetic variability and relationship of closely related Spanish traditional cultivars of tomato as detected by SRAP and SSR markers. *Journal of the American Society for Horticultural Science*.

[222] Williams CE, St Clair DA (1993). Phenetic relationships and levels of variability detected by restriction fragment length polymorphism and random amplified polymorphic DNA analysis of cultivated and wild accessions of *Lycopersicon esculentum*. *Genome*.

[223] Yang W, Bai X, Kabelka E (2004). Discovery of singly nucleotide polymorphisms in *Lycopersicon esculentum* by computer aided analysis of expressed sequence tags. *Molecular Breeding*.

[224] Baldo AM, Wan Y, Lamboy WF, Simon CJ, Labate JA, Sheffer SM NP validation and genetic diversity in cultivated tomatoes and grapes.

[225] Ganal MW, Durstewitz G, Kulosa D, Luerssen H, Polley A, Wolf M Development of EST-derived SNP markers for plant breeding.

[226] Sim S-C, Yang W, van der Knaap E, Hogenhout S, Xiao H, Francis DM Microarray-based SNP discovery for tomato genetics and breeding.

[227] Bernatzky R, Tanksley SD (1986). Toward a saturated linkage map in tomato based on isozymes and random cDNA cequences. *Genetics*.

[228] Tanksley SD, Ganal MW, Prince JP (1992). High density molecular linkage maps of the tomato and potato genomes. *Genetics*.

[229] Pillen K, Pineda O, Lewis C, Tanksley SD, Paterson AH (1996). Status of genome mapping tools in the taxon Solanaceae. *Genome Mapping in Plants*.

[230] Chen FQ, Foolad MR (1999). A molecular linkage map of tomato based on a cross between *Lycopersicon esculentum* and *L. pimpinellifolium* and its comparison with other molecular maps of tomato. *Genome*.

[231] Doganlar S, Frary A, Ku HM, Tanksley SD (2002). Mapping quantitative trait loci in inbred backcross lines of *Lycopersicon pimpinellifolium* (LA1589). *Genome*.

[232] Paterson AH, Damon S, Hewitt JD (1991). Mendelian factors underlying quantitative traits in tomato: comparison across species, generations, and environments. *Genetics*.

[233] Paran I, Goldman I, Tanksley SD, Zamir D (1995). Recombinant inbred lines for genetic mapping in tomato. *Theoretical and Applied Genetics*.

[234] Fulton TM, Grandillo S, Beck-Bunn T (2000). Advanced backcross QTL analysis of a *Lycopersicon esculentum*
×
*Lycopersicon parviflorum* cross. *Theoretical and Applied Genetics*.

[235] Bernacchi D, Tanksley SD (1997). An interspecific backcross of *Lycopersicon esculentum*
×
*L. hirsutum*: linkage analysis and a QTL study of sexual compatibility factors and floral traits. *Genetics*.

[236] Monforte AJ, Tanksley SD (2000). Development of a set of near isogenic and backcross recombinant inbred lines containing most of the *Lycopersicon hirsutum* genome in a *L. esculentum* genetic background: a tool for gene mapping and gene discovery. *Genome*.

[237] de Vicente MC, Tanksley SD (1993). QTL analysis of transgressive segregation in an interspecific tomato cross. *Genetics*.

[238] Eshed Y, Zamir D (1995). An introgression line population of *Lycopersicon pennellii* in the cultivated tomato enables the identification and fine mapping of yield- associated QTL. *Genetics*.

[239] Frary A, Fulton TM, Zamir D, Tanksley SD (2004). Advanced backcross QTL analysis of a *Lycopersicon esculentum*
×
*L. pennellii* cross and identification of possible orthologs in the Solanaceae. *Theoretical and Applied Genetics*.

[240] Fulton TM, Beck-Bunn T, Emmatty D (1997). QTL analysis of an advanced backcross of *Lycopersicon peruvianum* to the cultivated tomato and comparisons with QTLs found in other wild species. *Theoretical and Applied Genetics*.

[241] van Ooijen JW, Sandbrink JM, Vrielink M, Verkerk R, Zabel P, Lindhout P (1994). An RFLP linkage map of *Lycopersicon peruvianum*. *Theoretical and Applied Genetics*.

[242] Foolad MR, Zhang LP, Khan AA, Niño-Liu D, Lin GY (2002). Identification of QTLs for early blight (*Alternaria solani*) resistance in tomato using backcross populations of a *Lycopersicon esculentum*
×
*L. hirsutum* cross. *Theoretical and Applied Genetics*.

[243] Zhang LP, Lin GY, Niño-Liu D, Foolad MR (2003). Mapping QTLs conferring early blight (*Alternaria solani*) resistance in a *Lycopersicon esculentum*
×
*L. hirsutum* cross by selective genotyping. *Molecular Breeding*.

[244] Helentjaris T, King G, Slocum M, Siedenstrang C, Wegman S (1985). Restriction fragment polymorphisms as probes for plant diversity and their tools for applied plant breeding. *Plant Molecular Biology*.

[245] Bredemeijer GMM, Arens P, Wouters D, Visser D, Vosman B (1998). The use of semi-automated fluorescent microsatellite analysis for tomato cultivar identification. *Theoretical and Applied Genetics*.

[246] Isidore E, van Os H, Andrzejewski S (2003). Toward a marker-dense meiotic map of the potato genome: lessons from linkage group I. *Genetics*.

[247] van Os H, Stam P, Visser RGF, van Eck HJ (2005). RECORD: a novel method for ordering loci on a genetic linkage map. *Theoretical and Applied Genetics*.

[248] Lefebvre V, Pflieger S, Thabuis A (2002). Towards the saturation of the pepper linkage map by alignment of three intraspecific maps including known-function genes. *Genome*.

[249] Doganlar S, Frary A, Daunay M-C, Lester RN, Tanksley SD (2002). A comparative genetic linkage map of eggplant (*Solanum melongena*) and its implications for genome evolution in the Solanaceae. *Genetics*.

[250] Nunome T, Suwabe K, Iketani H, Hirai M (2003). Identification and characterization of microsatellites in eggplant. *Plant Breeding*.

[251] Sunseri F, Sciancalepore A, Martelli G (2003). Development of RAPD-AFLP map of eggplant and improvement of tolerance to *Verticillium* wilt. *Acta Horticulturae*.

[252] Strommer J, Gerats AGM, Sanago M, Molnar SJ (2000). A gene-based RFLP map of petunia. *Theoretical and Applied Genetics*.

[253] Strommer J, Peters J, Zethof J, De Keukeleire P, Gerats T (2002). AFLP maps of *Petunia hybrida*: building maps when markers cluster. *Theoretical and Applied Genetics*.

[254] Bindler G, van der Hoeven R, Gunduz I (2007). A microsatellite marker based linkage map of tobacco. *Theoretical and Applied Genetics*.

[255] Bonierbale MW, Plaisted RL, Tanksley SD (1988). RFLP maps based on a common set of clones reveal modes of chromosomal evolution in potato and tomato. *Genetics*.

[256] Tanksley SD, Miller J, Paterson A, Bernatzky R, Gustafson JP, Appels R (1988). Molecular mapping of plant chromosomes. *Chromosome Structure and Function*.

[257] Gebhardt C, Ritter E, Barone A, Debener B, Walkermeler B (1991). RFLP maps of potato and their alignment with the homologous
tomato genome. *Theoretical and Applied Genetics*.

[258] Livingstone KD, Lackney VK, Blauth JR, Van Wijk R, Jahn MK (1999). Genome mapping in capsicum and the evolution of genome structure in the Solanaceae. *Genetics*.

[259] Prince JP, Pochard E, Tanksley SD (1993). Construction of a molecular linkage map of pepper and a comparison of synteny with tomato. *Genome*.

[260] Buell CR, Rensink WA, Hart A, Rehfeld K, Liu J, Ly E Functional and comparative genomic resources for the *Solanaceae* at TIGR.

[261] Fridman E, Zamir D (2003). Functional divergence of a syntenic invertase gene family in tomato, potato, and Arabidopsis. *Plant Physiology*.

[262] Ku H-M, Vision T, Liu J, Tanksley SD (2000). Comparing sequenced segments of the tomato and *Arabidopsis* genomes: large-scale duplication followed by selective gene loss creates a network of synteny. *Proceedings of the National Academy of Sciences of the United States of America*.

[263] Lin C, Mueller LA, Carthy JM, Crouzillat D, Pétiard V, Tanksley SD (2005). Coffee and tomato share common gene repertoires as revealed by deep sequencing of seed and cherry transcripts. *Theoretical and Applied Genetics*.

[264] Frary A, Doganlar S, Daunay MC, Tanksley SD (2003). QTL analysis of morphological traits in eggplant and implications for conservation of gene function during evolution of solanaceous species. *Theoretical and Applied Genetics*.

[265] Grube RC, Radwanski ER, Jahn M (2000). Comparative genetics of disease resistance within the solanaceae. *Genetics*.

[266] Oh K, Hardeman K, Ivanchenko MG (2002). Fine mapping in tomato using microsynteny with the *Arabidopsis* genome: the *Diageotropica* (*Dgt*) locus. *Genome Biology*.

[267] Tanksley SD, Bernatzky R, Lapitan NL, Prince JP (1988). Conservation of gene repertoire but not gene order in pepper and tomato. *Proceedings of the National Academy of Sciences of the United States of America*.

[268] MacArthur JW (1934). Linkage groups in the tomato. *Journal of Genetics*.

[269] Bailey DM (1941). The seedling test method for root-knot nematode resistance. *Proceedings of the American Society for Horticultural Science*.

[270] Medina-Filho H (1980). Linkage of *Aps-1*, *Mi* and other markers on chromosome 6. *Report of the Tomato Genetics Cooperative*.

[271] Tanksley SD, Rick CM, Vallejos CE (1984). Tight linkage between a nuclear male-sterile locus and an enzyme marker in tomato. *Theoretical and Applied Genetics*.

[272] Tanksley SD, Loaiza-Figueroa F (1985). Gametophytic self-incompatibility is controlled by a single major locus on chromosome 1 in *Lycopersicon peruvianum*. *Proceedings of the National Academy of Sciences of the United States of America*.

[273] Tanksley SD, Medina-Filho H, Rick CM (1981). The effect of isozyme selection on metric characters in an interspecific backcross of tomato—basis of an early screening procedure. *Theoretical and Applied Genetics*.

[274] Tanksley SD, Medina-Filho H, Rick CM (1982). Use of naturally-occuring enzyme variation to detect and map genes controlling quantitative traits in an interspecific cross of tomato. *Heredity*.

[275] Vallejos CE, Tanksley SD (1983). Segregation of isozyme markers and cold tolerance in an interspecific backcross of tomato. *Theoretical and Applied Genetics*.

[276] Weller JI, Soller M, Brody T (1988). Linkage analysis of quantitative traits in an interspecific cross of tomato (*Lycopersicon esculentum*
×
*Lycopersicon pimpinellifolium*) by means of genetic markers. *Genetics*.

[277] Foolad MR, Zhang LP, Lin GY A genetic linkage map of tomato based on an 
*F* RIL population of a *Lycopersicon esculentum*
×
*L. pennellii* cross.

[278] Graham EB, Frary A, Kang JJ, Jones CM, Gardener RG (2004). A recombinant inbred line mapping population derived
from a *Lycopersicon esculentum*
×
*L. pimpinellifolium* cross. *Report of the Tomato Genetics Cooperative*.

[279] Goldman IL, Paran I, Zamir D (1995). Quantitative trait locus analysis of a recombinant inbred line population derived from
*Lycopersicon esculentum*
×
*Lycopersicon cheesmanii* cross. *Theoretical and Applied Genetics*.

[280] Paran I, Goldman I, Zamir D (1997). QTL analysis of morphological traits in a tomato recombinant inbred line population. *Genome*.

[281] Foolad MR (2004). Recent advances in genetics of salt tolerance in tomato. *Plant Cell, Tissue and Organ Culture*.

[282] Diwan N, Fluhr R, Eshed Y, Zamir D, Tanksley SD (1999). Mapping of *Ve* in tomato: a gene conferring resistance to the broad-spectrum pathogen, *Verticillium dahliae* race 1. *Theoretical and Applied Genetics*.

[283] Bernacchi D, Beck-Bunn T, Eshed Y (1998). Advanced backcross QTL analysis in tomato. I. Identification of QTLs for traits of agronomic importance from *Lycopersicon hirsutum*. *Theoretical and Applied Genetics*.

[284] Bliss FA (1982). The inbred backcross line method for improving quantitative traits of self-pollinated crops. *HortScience*.

[285] Tanksley SD, Nelson JC (1996). Advanced backcross QTL analysis: a method for the simultaneous discovery and transfer of valuable QTLs from unadapted germplasm into elite breeding lines. *Theoretical and Applied Genetics*.

[286] Tanksley SD, Grandillo S, Fulton TM (1996). Advanced backcross QTL analysis in a cross between an elite processing line of tomato and its wild relative *L. pimpinellifolium*. *Theoretical and Applied Genetics*.

[287] Coaker GL, Francis DM (2004). Mapping, genetic effects, and epistatic interaction of two bacterial canker resistance QTLs from *Lycopersicon hirsutum*. *Theoretical and Applied Genetics*.

[288] Robert VJM, West MAL, Inai S (2001). Marker-assisted introgression of blackmold resistance QTL alleles from wild *Lycopersicon cheesmanii* to cultivated tomato (*L. esculentum*) and evaluation of QTL phenotypic effects. *Molecular Breeding*.

[289] Zamir D, Ekstein-Michelson I, Zakay Y (1994). Mapping and introgression of a tomato yellow leaf curl virus tolerance gene, *Ty-1*. *Theoretical and Applied Genetics*.

[290] Zamir D (2001). Improving plant breeding with exotic genetic libraries. *Nature Reviews Genetics*.

[291] Eshed Y, Abu-Abied M, Saranga Y, Zamir D (1992). *Lycopersicon esculentum* lines containing small overlapping introgressions from *L. pennellii*. *Theoretical and Applied Genetics*.

[292] Eshed Y, Zamir D (1994). A genomic library of *Lycopersicon pennellii* in *L. esculentum*: a tool for fine mapping of genes. *Euphytica*.

[293] Gur A, Semel Y, Cahaner A, Zamir D (2004). Real time QTL of complex phenotypes in tomato interspecific introgression lines. *Trends in Plant Science*.

[294] Liu Y-S, Gur A, Ronen G (2003). There is more to tomato fruit colour than candidate carotenoid genes. *Plant Biotechnology Journal*.

[295] Liu Y-S, Zamir D (1999). Second generation *L. pennellii* introgression lines and the concept of bin mapping. *Report of the Tomato Genetics Cooperative*.

[296] Canady MA, Meglic V, Chetelat RT (2005). A library of *Solanum lycopersicoides* introgression liines in cultivated tomato. *Genome*.

[297] Causse M, Duffe P, Gomez MC (2004). A genetic map of candidate genes and QTLs involved in tomato fruit size and composition. *Journal of Experimental Botany*.

[298] Eshed Y, Zamir D (1994). Introgressions from *Lycopersicon pennellii* can improve the soluble solids yield of tomato hybrids. *Theoretical and Applied Genetics*.

[299] Ronen G, Cohen M, Zamir D, Hirschberg J (1999). Regulation of carotenoid biosynthesis during tomato fruit development: expression of the gene for lycopene epsilon-cyclase is down-regulated during ripening and is elevated in the mutant *Delta*. *The Plant Journal*.

[300] Rousseaux MC, Jones CM, Adams D, Chetelat R, Bennett A, Powell A (2005). QTL analysis of fruit antioxidants in tomato using *Lycopersicon pennellii* introgression lines. *Theoretical and Applied Genetics*.

[301] Isaacson T, Ronen G, Zamir D, Hirschberg J (2002). Cloning of tangerine from tomato reveals a carotenoid isomerase essential for the production of 
*β*-carotene and xanthophylls in plants. *The Plant Cell*.

[302] Fridman E, Carrari F, Liu Y-S, Fernie AR, Zamir D (2004). Zooming in on a quantitative trait for tomato yield using interspecific introgressions. *Science*.

[303] Fridman E, Liu Y-S, Carmel-Goren L (2002). Two tightly linked QTLs modify tomato sugar content via different physiological pathways. *Molecular Genetics and Genomics*.

[304] Yates HE, Frary A, Doganlar S (2004). Comparative fine mapping of fruit quality QTLs on chromosome 4 introgressions derived from two wild tomato species. *Euphytica*.

[305] Frary A, Nesbitt TC, Frary A (2000). *fw2.2*: a quantitative trait locus key to the evolution of tomato fruit size. *Science*.

[306] van der Knaap E, Sanyal A, Jackson SA, Tanksley SD (2004). High-resolution fine mapping and fluorescence in situ hybridization analysis of sun, a locus controlling tomato fruit shape, reveals a region of the tomato genome prone to DNA rearrangements. *Genetics*.

[307] Chen K-Y, Tanksley SD (2004). High-resolution mapping and functional analysis of *se2.1*: a major stigma exsertion quantitative trait locus associated with the evolution from allogamy to autogamy in the genus *Lycopersicon*. *Genetics*.

[308] Gur A, Zamir D (2004). Unused natural variation can lift yield barriers in plant breeding. *PLoS Biology*.

[309] Mesbah LA, Kneppers TJA, Takken FLW, Laurent P, Hille J, Nijkamp HJJ (1999). Genetic and physical analysis of a YAC contig spanning the fungal disease resistance locus *Asc* of tomato (*Lucopersicon esculentum*). *Molecular and General Genetics*.

[310] van der Biezen EA, Glagotskaya T, Overduin B, Nijkamp HJJ, Hille J (1995). Inheritance and genetic mapping of resistance to *Alternaria alternata* f. sp. *lycopersici* in *Lycopersicon pennellii*. *Molecular and General Genetics*.

[311] Kaloshian I, Lange WH, Williamson VM (1995). An aphid-resistance locus is tightly linked to the nematode-resistance gene, *Mi*, in tomato. *Proceedings of the National Academy of Sciences of the United States of America*.

[312] Kaloshian I, Yaghoobi J, Liharska T (1998). Genetic and physical localization of the root-knot nematode resistance locus *Mi* in tomato. *Molecular and General Genetics*.

[313] Sandbrink JM, van Ooijen J, Purimahua CC (1995). Localization of genes for bacterial canker resistance in *Lycopersicon peruvianum* using RFLPs. *Theoretical and Applied Genetics*.

[314] van Heusden AW, Koornneef M, Voorrips RE (1999). Three QTLs from *Lycopersicon peruvianum* confer a high level of resistance to *Clavibacter michiganensis* ssp. *michiganensis*. *Theoretical and Applied Genetics*.

[315] Kabelka E, Franchino B, Francis DM (2002). Two loci from *Lycopersicon hirsutum* LA407 confer resistance to strains of *Clavibacter michiganensis* subsp. *michiganensis*. *Phytopathology*.

[316] Martin GB, Williams JGK, Tanksley SD (1991). Rapid identification of markers linked to a Pseudomonas resistance gene in tomato by using random primers and near-isogenic lines. *Proceedings of the National Academy of Sciences of the United States of America*.

[317] Salmeron JM, Oldroyd GED, Rommens CMT (1996). Tomato *Prf* is a member of the leucine-rich repeat class of plant disease resistance genes and lies embedded within the *Pto* kinase gene cluster. *Cell*.

[318] Astua-Monge G, Minsavage GV, Stall RE, Vallejos CE, Davis MJ, Jones JB (2000). *Xv4-vrxv4*: a new gene-for-gene interaction identified between *Xanthomonas campestris* pv. *vesicatoria* race T3 and the wild tomato relative *Lycopersicon pennellii*. *Molecular Plant-Microbe Interactions*.

[319] Ballvora A, Pierre M, van den Ackerveken G (2001). Genetic mapping and functional analysis of the tomato *Bs4* locus governing recognition of the *Xanthomonas campestris* pv. *vesicatoria* AvrBs4 protein. *Molecular Plant-Microbe Interactions*.

[320] Danesh D, Aarons S, McGill GE, Young ND (1994). Genetic dissection of oligogenic resistance to bacterial wilt in tomato. *Molecular Plant-Microbe Interactions*.

[321] Deberdt P, Olivier J, Thoquet P, Quénéhervé P, Prior P (1999). Evaluation of bacterial wilt resistance in tomato lines nearly isogenic for the *Mi* gene for resistance to root-knot. *Plant Pathology*.

[322] Mangin B, Thoquet P, Olivier J, Grimsley NH (1999). Temporal and multiple quantitative trait loci analyses of resistance to bacterial wilt in tomato permit the resolution of linked loci. *Genetics*.

[323] Thoquet P, Olivier J, Sperisen C, Rogowsky P, Laterrot H, Grimsley N (1996). Quantitative trait loci determining resistance to bacterial wilt in tomato cultivar Hawaii 7996. *Molecular Plant-Microbe Interactions*.

[324] Thoquet P, Olivier J, Sperisen C (1996). Polygenic resistance of tomato plants to bacterial wilt in the French West Indies. *Molecular Plant-Microbe Interactions*.

[325] Doganlar S, Dodson J, Gabor B, Beck-Bunn T, Crossman C, Tanksley SD (1998). Molecular mapping of the *py-1* gene for resistance to corky root rot (*Pyrenochaeta lycopersici*) in tomato. *Theoretical and Applied Genetics*.

[326] Stamova BS, Chetelat RT (2000). Inheritance and genetic mapping of cucumber mosaic virus resistance introgressed from *Lycopersicon chilense* into tomato. *Theoretical and Applied Genetics*.

[327] Vakalounakis DJ, Laterrot H, Moretti A, Ligoxigakis EK, Smardas K (1997). Linkage between *Fr1 (Fusarium oxysporum* f. sp. *radicis-lycopersici* resistance) and *Tm-2* (tobacco mosaic virus resistance-2) loci in tomato (*Lycopersicon esculentum*). *Annals Applied Biology*.

[328] Bournival BL, Scott JW, Vallejos CE (1989). An isozyme marker for resistance to race 3 of *Fusarium oxysporum* f. sp. *lycopersici* in tomato. *Theoretical and Applied Genetics*.

[329] Bournival BL, Vallejos CE, Scott JW (1990). Genetic analysis of resistances to races 1 and 2 of *Fusarium oxysporum* f. sp. *lycopersici* from the wild tomato *Lycopersicon pennellii*. *Theoretical and Applied Genetics*.

[330] Ori N, Eshed Y, Paran I (1997). The 
*I* family from the wilt disease resistance locus 
*I* belongs to the nucleotide binding, leucine-rich repeat superfamily of plant resistance genes. *The Plant Cell*.

[331] Sarfatti M, Abu-Abied M, Katan J, Zamir D (1991). RFLP mapping of 
*I*, a new locus in tomato conferring resistance against *Fusarium oxysporum* f. sp. *lycopersici* race 1. *Theoretical and Applied Genetics*.

[332] Sarfatti M, Katan J, Fluhr R, Zamir D (1989). An RFLP marker in tomato linked to the *Fusarium oxysporum* resistance gene 
*I*. *Theoretical and Applied Genetics*.

[333] Scott JW, Agrama HA, Jones JP (2004). RFLP-based analysis of recombination among resistance genes to fusarium wilt races 1, 2, and 3 in tomato. *Journal of the American Society for Horticultural Science*.

[334] Segal G, Sarfatti M, Schaffer MA, Ori N, Zamir D, Fluhr R (1992). Correlation of genetic and physical structure in the region surrounding the 
*I*
*Fusarium oxysporum* resistance locus in tomato. *Molecular and General Genetics*.

[335] Simons G, Groenendijk J, Wijbrandi J (1998). Dissection of the fusarium 
*I* gene cluster in tomato reveals six homologs and one active gene copy. *The Plant Cell*.

[336] Tanksley SD, Costello W (1991). The size of the *L. pennellii* chromosome 7 segment containing the *I-3* gene in tomato breeding lines measured by RFLP probing. *Report of the Tomato Genetics Cooperative*.

[337] Behare J, Laterrot H, Sarfatti M, Zamir D (1991). RFLP mapping of the *Stemphylium* resistance gene in tomato. *Molecular Plant-Microbe Interactions*.

[338] Chunwongse J, Chunwongse C, Black L, Hanson P (1998). Mapping of *Ph-3* gene for late blight from *L. pimpinellifolium* L3708. *Report of the Tomato Genetics Cooperative*.

[339] Moreau P, Thoquet P, Olivier J, Laterrot H, Grimsley N (1998). Genetic mapping of *Ph-2*, a single locus controlling partial resistance to *Phytophthora infestans* in tomato. *Molecular Plant-Microbe Interactions*.

[340] Pierce LC (1971). Linkage test with *Ph* conditioning resistance to race 0. *Report of the Tomato Genetics Cooperative*.

[341] Brouwer DJ, Jones ES, St Clair DA (2004). QTL analysis of quantitative resistance to *Phytophthora infestans* (late blight) in tomato and comparison with potato. *Genome*.

[342] Brouwer DJ, St Clair DA (2004). Fine mapping of three quantitative trait loci for late blight resistance in tomato using near isogenic lines (NILs) and sub-NILs. *Theoretical and Applied Genetics*.

[343] Frary A, Graham E, Jacobs J, Chetelat RT, Tanksley SD (1998). Identification of QTL for late blight resistance from *L. pimpinellifolium* L3708. *Report of the Tomato Genetics Cooperative*.

[344] Balint Kurti PJ, Dixon MS, Jones DA, Norcott KA, Jones JDG (1994). RFLP linkage analysis of the *Cf-4* and *Cf-9* genes for resistance to *Cladosporium fulvum* in tomato. *Theoretical and Applied Genetics*.

[345] Jones DA, Dickinson MJ, Balint-Kurti PJ, Dixon MS, Jones JDG (1993). Two complex resistance loci revealed in tomato by classical and RFLP mapping of *Cf-2*, *Cf-4*, *Cf-5*, and 
*Cf-9* genes for resistance to *Cladosporium fulvum*. *Molecular Plant-Microbe Interactions*.

[346] Laugé R, Dmitriev AP, Joosten MHAJ, De Wit PJGM (1998). Additional resistance gene(s) against *Cladosporium fulvum* present on the *Cf-9* introgression segment are associated with strong PR protein accumulation. *Molecular Plant-Microbe Interactions*.

[347] van der Beek JG, Verkerk R, Zabel P, Lindhout P (1992). Mapping strategy for resistance genes in tomato based on RFLPs between cultivars: *Cf9* (resistance to *Cladosporium fulvum*) on chromosome 1. *Theoretical and Applied Genetics*.

[348] Ganal MW, Simon R, Brommonschenkel S (1995). Genetic mapping of a wide spectrum nematode resistance gene (*Hero*) against *Globodera rostochiensis* in tomato. *Molecular Plant-Microbe Interactions*.

[349] Aarts JM, Hontelez JG, Fischer P, Verkerk R, van Kammen A, Zabel P (1991). Acid phosphatase-
*1*, a tightly linked molecular marker for root-knot nematode resistance in tomato: from protein to gene, using PCR and degenerate primers containing deoxyinosine. *Plant Molecular Biology*.

[350] Ammiraju JSS, Veremis JC, Huang X, Roberts PA, Kaloshian I (2003). The heat-stable root-knot nematode resistance gene *Mi-9* from *Lycopersicon peruvianum* is localized on the short arm of chromosome 6. *Theoretical and Applied Genetics*.

[351] Doganlar S, Frary A, Tanksley SD (1997). Production of interspecific 
*F* hybrids, 
*BC*, 
*BC* and 
*BC* populations between *Lycopersicon esculentum* and two accessions of *Lycopersicon peruvianum* carrying new root-knot nematode resistance genes. *Euphytica*.

[352] Klein-Lankhorst R, Rietveld P, Machiels B (1991). RFLP markers linked to the root knot nematode resistance gene *Mi* in tomato. *Theoretical and Applied Genetics*.

[353] Veremis JC, van Heusden AW, Roberts PA (1999). Mapping a novel heat-stable resistance to *Meloidogyne* in *Lycopersicon peruvianum*. *Theoretical and Applied Genetics*.

[354] Williamson VM, Ho JY, Wu FF, Miller N, Kaloshian I (1994). A PCR-based marker tightly linked to the nematode resistance gene, *Mi*, in tomato. *Theoretical and Applied Genetics*.

[355] Yaghoobi J, Kaloshian I, Wen Y, Williamson VM (1995). Mapping of a new nematode resistance locus in *Lycopersicon peruvianum*. *Theoretical and Applied Genetics*.

[356] Parrella G, Ruffel S, Moretti A, Morel C, Palloix A, Caranta C (2002). Recessive resistance genes against potyviruses are localized in colinear genomic regions of the tomato (*Lycopersicon* spp.) and pepper (*Capsicum* spp.) genomes. *Theoretical and Applied Genetics*.

[357] Chungwongse J, Bunn TB, Crossman C, Jiang J, Tanksley SD (1994). Chromosomal localization and molecular marker tagging of the powdery mildew resistance gene (*Lv*) in tomato. *Theoretical and Applied Genetics*.

[358] de Giovanni C, Dell'Orco P, Bruno A, Ciccarese F, Lotti C, Ricciardi L (2004). Identification of PCR-based markers (RAPD, AFLP) linked to a novel powdery mildew resistance gene (*ol-2*) in tomato. *Plant Science*.

[359] van der Beek JG, Pet G, Lindhout P (1994). Resistance to powdery mildew *(Oidium lycopersicum)* in *Lycopersicon hirsutum* is controlled by an incompletely-dominant gene *Ol-1* on chromosome 6. *Theoretical and Applied Genetics*.

[360] Bai Y, Huang C-C, van der Hulst R, Meijer-Dekens F, Bonnema G, Lindhout P (2003). QTLs for tomato powdery mildew resistance (*Oidium lycopersici*) in *Lycopersicon parviflorum* G1.1601 co-localize with two qualitative powdery mildew resistance genes. *Molecular Plant-Microbe Interactions*.

[361] Levesque H, Vedel F, Mathieu C, de Courcel AGL (1990). Identification of a short rDNA spacer sequence highly specific of a tomato line containing *Tm-1* gene introgressed from *Lycopersicon hirsutum*. *Theoretical and Applied Genetics*.

[362] Ohmori T, Murata M, Motoyoshi F (1996). Molecular characterization of RAPD and SCAR markers linked to the *Tm-1* locus in tomato. *Theoretical and Applied Genetics*.

[363] Tanksley SD, Bernachi D, Beck-Bunn T (1998). Yield and quality evaluations on a pair of processing tomato lines nearly isogenic for the 
*T* gene for resistance to the tobacco mosaic virus. *Euphytica*.

[364] Young ND, Zamir D, Ganal MW, Tanksley SD (1988). Use of isogenic lines and simultaneous probing to identify DNA markers tightly linked to the *Tm-2a* gene in tomato. *Genetics*.

[365] Griffiths PD, Scott JW (2001). Inheritance and linkage of tomato mottle virus resistance genes derived from *Lycopersicon chilense* accession LA 1932. *Journal of the American Society for Horticultural Science*.

[366] Stevens MR, Lamb EM, Rhoads DD (1995). Mapping the *Sw-5* locus for tomato spotted wilt virus resistance in tomatoes using RAPD and RFLP analyses. *Theoretical and Applied Genetics*.

[367] Chagué V, Mercier JC, Guénard M, de Courcel A, Vedel F (1997). Identification of RAPD markers linked to a locus involved in quantitative resistance to TYLCV in tomato by bulked segregant analysis. *Theoretical and Applied Genetics*.

[368] Hanson PM, Bernacchi D, Green S (2000). Mapping a wild tomato introgression associated with tomato yellow leaf curl virus resistance in a cultivated tomato line. *Journal of the American Society for Horticultural Science*.

[369] Hanson PM, Green SK, Kuo G (2006). *Ty-2*, a gene on chromosome 11 conditioning geminivirus
resistance in tomato. *Report of the Tomato Genetics Cooperative*.

[370] Ji Y-F, Scott JW (2006). *Ty-3*, a begomovirus resistance locus linked to *Ty-1* on chromosome 6 of tomato. *Report of the Tomato Genetics Cooperative*.

[371] Kawchuk LM, Hachey J, Lynch DR (1998). Development of sequence characterized DNA markers linked to a dominant verticillium wilt resistance gene in tomato. *Genome*.

[372] Rick CM, Fobes JF (1974). Association of an allozyme with nematode resistance. *Report of the Tomato Genetics Cooperative*.

[373] Gilbert JC, McQuire DC (1955). One major gene for resistance to sever galling from *Meloidogyne incognita*. *Report of the Tomato Genetics Cooperative*.

[374] Hanson PM, Licardo O, Hanudin M, Wang J-F, Chen J-T (1998). Diallel analysis of bacterial wilt resistance in tomato derived from different sources. *Plant Disease*.

[375] Scott JW, Jones JB, Somodi GC (2001). Inheritance of resistance in tomato to race T3 of the bacterial spot pathogen. *Journal of the American Society for Horticultural Science*.

[376] Lawson DM, Lunde CF, Mutschler MA (1997). Marker-assisted transfer of acylsugar-mediated pest resistance from the wild tomato, *Lycopersicon pennellii*, to the cultivated tomato, *Lycopersicon esculentum*. *Molecular Breeding*.

[377] Blauth SL, Steffens JC, Churchill GA, Mutschler MA (1999). Identification of QTLs controlling acylsugar fatty acid composition in an intraspecific population of *Lycopersicon pennellii* (Corr.) D'Arcy. *Theoretical and Applied Genetics*.

[378] Foolad MR, Chen FQ, Lin GY (1998). RFLP mapping of QTLs conferring cold tolerance during seed germination in an interspecific cross of tomato. *Molecular Breeding*.

[379] Truco MJ, Randall LB, Bloom AJ, St Clair DA (2000). Detection of QTLs associated with shoot wilting and root ammonium uptake under chilling temperatures in an interspecific backcross population from *Lycopersicon esculentum*
×
*L. hirsutum*. *Theoretical and Applied Genetics*.

[380] Martin B, Nienhuis J, King G, Schaefer A (1989). Restriction fragment length polymorphisms associated with water
use efficiency in tomato. *Science*.

[381] Foolad MR, Jones RA (1993). Mapping salt-tolerance genes in tomato (*Lycopersicon esculentum*) using trait-based marker analysis. *Theoretical and Applied Genetics*.

[382] Foolad MR, Stoltz T, Dervinis C, Rodriguez RL, Jones RA (1997). Mapping QTLs conferring salt tolerance during germination in tomato by selective genotyping. *Molecular Breeding*.

[383] Foolad MR, Chen FQ (1998). RAPD markers associated with salt tolerance in an interspecific cross of tomato (*Lycopersicon esculentum*
×
*L. pennellii*). *Plant Cell Reports*.

[384] Foolad MR, Chen FQ (1999). RFLP mapping of QTLs conferring salt tolerance during vegetative
stage in tomato. *Theoretical and Applied Genetics*.

[385] Foolad MR, Zhang LP, Lin GY (2001). Identification and validation of QTLs for salt tolerance during vegetative growth in tomato by selective genotyping. *Genome*.

[386] Zamir D, Tal M (1987). Genetic analysis of sodium, potassium and chloride ion content in *Lycopersicon*. *Euphytica*.

[387] Bretó MP, Asins MJ, Carbonell EA (1994). Salt tolerance in *Lycopersicon* species. III. Detection of quantitative trait loci by means of molecular markers. *Theoretical and Applied Genetics*.

[388] Vos P, Simons G, Jesse T (1998). The tomato *Mi-1* gene confers resistance to both root-knot nematodes and potato aphids. *Nature Biotechnology*.

[389] Milligan SB, Bodeau J, Yaghoobi J, Kaloshian I, Zabel P, Williamson VM (1998). The root knot nematode resistance gene *Mi* from tomato is a member of the leucine zipper, nucleotide binding, leucine-rich repeat family of plant genes. *The Plant Cell*.

[390] Hammond-Kosack KE, Jones JDG (1997). Plant disease resistance genes. *Annual Review of Plant Physiology and Plant Molecular Biology*.

[391] Goggin FL, Williamson VM, Ullman DE (2001). Variability in the response of *Macrosiphum euphorbiae* and *Myzus persicae* (Hemiptera: Aphididae) to the tomato resistance gene *Mi*. *Environmental Entomology*.

[392] Scott JW, Everett PH, Bryan HH (1985). Suncoast—a large-fruited home garden tomato. *Florida Agricultural Experiment Stations Circular*.

[393] Foolad MR, Jones RA (1991). Genetic analysis of salt tolerance during germination in *Lycopersicon*. *Theoretical and Applied Genetics*.

[394] Foolad MR (1996). Genetic analysis of salt tolerance during vegetative growth in tomato, *Lycopersicon esculentum* Mill. *Plant Breeding*.

[395] Foolad MR (1997). Genetic basis of physiological traits related to salt tolerance in tomato, *Lycopersicon esculentum* Mill. *Plant Breeding*.

[396] Monforte AJ, Asíns MJ, Carbonell EA (1996). Salt tolerance in *Lycopersicon* species. IV. Efficiency of marker-assisted selection for salt tolerance improvement. *Theoretical and Applied Genetics*.

[397] Monforte AJ, Asíns MJ, Carbonell EA (1997). Salt tolerance in *Lycopersicon* species. V. Does genetic variability at quantitative trait loci affect their analysis?. *Theoretical and Applied Genetics*.

[398] Monforte AJ, Asíns MJ, Carbonell EA (1999). Salt tolerance in *Lycopersicon* spp. VII. Pleiotropic action of genes controlling earliness on fruit yield. *Theoretical and Applied Genetics*.

[399] Zhang LP, Lin GY, Foolad MR (2003). QTL comparison of salt tolerance during seed germination and vegetative growth in a *Lycopersicon esculentum*
×
*L. pimpinellifolium* RIL population. *Acta Horticulturae*.

[400] Foolad MR (2000). Genetics bases of salt tolerance and cold tolerance in tomato. *Current Topics in Plant Biology*.

[401] Foolad MR, Lin GY, Chen FQ (1999). Comparison of QTLs for seed germination under non-stress, cold stress and salt stress in tomato. *Plant Breeding*.

[402] Foolad MR, Zhang LP, Lin GY Comparison of QTLs for cold, salt and drought tolerance during
seed germination in an 
*F* RIL population of tomato.

[403] Foolad MR, Zhang LP, Subbiah P (2003). Relationships among cold, salt and drought tolerance during seed germination in tomato: inheritance and QTL mapping. *Acta Horticulturae*.

[404] Frary A, Fritz LA, Tanksley SD (2004). A comparative study of the genetic bases of natural variation in tomato leaf, sepal, and petal morphology. *Theoretical and Applied Genetics*.

[405] Monforte AJ, Tanksley SD (2000). Fine mapping of a quantitative trait locus (QTL) from *Lycopersicon hirsutum* chromosome I affecting fruit characteristics and agronomic traits: breaking linkage among QTLs affecting different traits and dissection of heterosis for yield. *Theoretical and Applied Genetics*.

[406] Frary A, Doganlar S, Frampton A (2003). Fine mapping of quantitative trait loci for improved fruit characteristics from *Lycopersicon chmielewskii* chromosome 1. *Genome*.

[407] Ito P, Currence TM (1964). A linkage test involving c *sp B+ md* in chromosome 6. *Report of the Tomato Genetics Cooperative*.

[408] Lincoln RE, Porter JW (1950). Inheritance of beta-carotene in tomatoes. *Genetics*.

[409] Zhang Y, Stommel JR (2000). RAPD and AFLP tagging and mapping of Beta (*B*) and Beta modifier 
*(*, two genes which influence 
*β*-carotene accumulation in fruit of tomato (*Lycopersicon esculentum* Mill.). *Theoretical and Applied Genetics*.

[410] Saliba-Colombani V, Causse M, Langlois D, Philouze J, Buret M (2001). Genetic analysis of organoleptic quality in fresh market tomato. 1. Mapping QTLs for physical and chemical traits. *Theoretical and Applied Genetics*.

[411] Tomes ML, Erickson HT, Barman RJ (1966). Location, inheritance, and modification of flower color. *Report of the Tomato Genetics Cooperative*.

[412] Peters JL, Széll M, Kendrick RE (1998). The expression of light-regulated genes in the high-pigment-1 mutant of tomato. *Plant Physiology*.

[413] Mustilli AC, Fenzi F, Ciliento R, Alfano F, Bowler C (1999). Phenotype of the tomato *high pigment-2* mutant is caused by a mutation in the tomato homolog of DEETIOLATED1. *The Plant Cell*.

[414] Chen FQ, Foolad MR, Hyman J, St Clair DA, Beelaman RB (1999). Mapping of QTLs for lycopene and other fruit traits in a *Lycopersicon esculentum*
×
*L. pimpinellifolium* cross and comparison of QTLs across tomato species. *Molecular Breeding*.

[415] Causse M, Saliba-Colombani V, Lesschaeve I, Buret M (2001). Genetic analysis of organoleptic quality in fresh market tomato. 2. Mapping QTLs for sensory attributes. *Theoretical and Applied Genetics*.

[416] Azanza F, Kim D, Tanksley SD, Juvik JA (1995). Genes from *Lycopersicon chmielewskii* affecting tomato quality during fruit ripening. *Theoretical and Applied Genetics*.

[417] Doganlar S, Tanksley SD, Mutschler MA (2000). Identification and molecular mapping of loci controlling fruit ripening time in tomato. *Theoretical and Applied Genetics*.

[418] Slater A, Maunders MJ, Edwards K, Schuch W, Grierson D (1985). Isolation and characterisation of cDNA clones for tomato polygalacturonase and other ripening-related proteins. *Plant Molecular Biology*.

[419] Thompson AJ, Tør M, Barry CS (1999). Molecular and genetic characterization of a novel pleiotropic tomato-ripening mutant. *Plant Physiology*.

[420] Tør M, Manning K, King GJ (2002). Genetic analysis and FISH mapping of the *Colourless non-ripening* locus of tomato. *Theoretical and Applied Genetics*.

[421] Lanahan MB, Yen HC, Giovannoni JJ, Klee HJ (1994). The *Never ripe* mutation blocks ethylene perception in tomato. *The Plant Cell*.

[422] Wilkinson JQ, Lanahan MB, Yen H-C, Giovannoni JJ, Klee HJ (1995). An ethylene-inducible component of signal transduction encoded by *Never-ripe*. *Science*.

[423] Yen H-C, Lee S, Tanksley SD, Lanahan MB, Klee HJ, Giovannoni JJ (1995). The tomato *Never-ripe* locus regulates ethylene-inducible gene experession and is linked to a homologue of the *Arabidopsis ETR1* gene. *Plant Physiology*.

[424] Giovannoni JJ, Noensie EN, Ruezinsky DM (1995). Molecular genetic analysis of the *ripening-inhibitor* and *non-ripening* loci of tomato: a first step in genetic map-based cloning of fruit ripening genes. *Molecular and General Genetics*.

[425] Vrebalov J, Ruezinsky D, Padmanabhan V (2002). A MADS-box gene necessary for fruit ripening at the tomato *ripening-inhibitor (rin)* locus. *Science*.

[426] van der Knaap E, Lippman ZB, Tanksley SD (2002). Extremely elongated tomato fruit controlled by four quantitative trait loci with epistatic interactions. *Theoretical and Applied Genetics*.

[427] van der Knaap E, Tanksley SD (2003). The making of a bell pepper-shaped tomato fruit: identification of loci controlling fruit morphology in yellow stuffer tomato. *Theoretical and Applied Genetics*.

[428] Ku H-M, Doganlar S, Chen K-Y, Tanksley SD (1999). The genetic basis of pear-shaped tomato fruit. *Theoretical and Applied Genetics*.

[429] Osborn TC, Alexander DC, Fobes JF (1987). Identification of restriction fragment length polymorhisms linked to genes controlling soluble solids content in tomato. *Theoretical and Applied Genetics*.

[430] Paterson AH, DeVerna JW, Lanini B, Tanksley SD (1990). Fine mapping of quantitative trait loci using selected overlapping recombinant chromosomes, in an interspecies cross of tomato. *Genetics*.

[431] Wing RA, Zhang H-B, Tanksley SD (1994). Map-based cloning in crop plants. Tomato as a model system: I. Genetic and physical mapping of *jointless*. *Molecular and General Genetics*.

[432] Zhang H-B, Budiman MA, Wing RA (2000). Genetic mapping of *jointless-2* to tomato chromosome 12 using RFLP and RAPD markers. *Theoretical and Applied Genetics*.

[433] Bernatzky R (1993). Genetic mapping and protein product diversity of the self-incompatibility locus in wild tomato (*Lycopersicon peruvianum*). *Biochemical Genetics*.

[434] Coaker GL, Meulia T, Kabelka EA, Jones AK, Francis DM (2002). A QTL controlling stem morphology and vascular development in *Lycopersicon esculentum*
×
*Lycopersicon hirsutum* (Solanaceae) crosses is located on chromosome 2. *American Journal of Botany*.

[435] Alpert KB, Grandillo S, Tanksley SD (1995). *fw 2.2*: a major QTL controlling fruit weight is common to both red- and green-fruited tomato species. *Theoretical and Applied Genetics*.

[436] Young PA, MacArthur JW (1947). Horticultural characters of tomatoes.

[437] Grandillo S, Ku H-M, Tanksley SD (1996). Characterization of *fs8.1*, a major QTL influencing fruit shape in tomato. *Molecular Breeding*.

[438] Ku H-M, Grandillo S, Tanksley SD (2000). *fs8.1*, a major QTL, sets the pattern of tomato carpel shape well before anthesis. *Theoretical and Applied Genetics*.

[439] Liu J, Van Eck J, Cong B, Tanksley SD (2002). A new class of regulatory genes underlying the cause of pear-shaped tomato fruit. *Proceedings of the National Academy of Sciences of the United States of America*.

[440] Di Mascio P, Kaiser S, Sies H (1989). Lycopene as the most efficient biological carotenoid singlet oxygen quencher. *Archives of Biochemistry and Biophysics*.

[441] Kohlmeier L, Kark JD, Gomez-Gracia E (1997). Lycopene and myocardial infarction risk in the EURAMIC study. *American Journal of Epidemiology*.

[442] Levy J, Bosin E, Feldman B (1995). Lycopene is a more potent inhibitor of human cancer cell proliferation than either 
*α*-carotene or 
*β*-carotene. *Nutrition and Cancer*.

[443] Stahl W, Sies H (1996). Lycopene: a biologically important carotenoid for humans?. *Archives of Biochemistry and Biophysics*.

[444] Wann EV, Jourdain EL (1985). Effects of mutant genotypes *hp*
*o* and *dg*
*o* on tomato fruit quality. *Journal of the American Society for Horticultural Science*.

[445] Römer S, Fraser PD, Kiano JW (2000). Elevation of the provitamin A content of transgenic tomato plants. *Nature Biotechnology*.

[446] Fox E, Giovannoni J, Razdan MK, Mattoo AK (2007). Genetic control of fruit ripening. *Genetic Improvement of Sloanaceous Crops*.

[447] Yang W, Francis DM (2005). Marker-assisted selection for combining resistance to bacterial spot and bacterial speck in tomato. *Journal of the American Society for Horticultural Science*.

[448] Ribaut J-M, Jiang C, Hoisington D (2002). Simulation experiments on efficiencies of gene introgression by backcrossing. *Crop Science*.

[449] Servin B, Martin OC, Mézard M, Hospital F (2004). Toward a theory of marker-assisted gene pyramiding. *Genetics*.

[450] Tanksley SD, Young ND, Paterson AH, Bonierbale MW (1989). RFLP mapping in plant breeding: new tools for an old science. *Bio/Technology*.

[451] Bouchez A, Hospital F, Causse M, Gallais A, Charcosset A (2002). Marker-assisted introgression of favorable alleles at quantitative trait loci between maize elite lines. *Genetics*.

[452] Eathington SR, Dudley JW, Rufener GK (1997). Usefulness of marker-QTL associations in early generation selection. *Crop Science*.

[453] Hospital F, Goldringer I, Openshaw S (2000). Efficient marker-based recurrent selection for multiple quantitative trait loci. *Genetical Research*.

[454] Jiang GH, Xu CG, Tu JM, Li XH, He YQ, Zhang QF (2004). Pyramiding of insect- and disease-resistance genes into an elite *indica*, cytoplasm male sterile restorer line of rice, ‘Minghui 63’. *Plant Breeding*.

[455] Knapp SJ (1998). Marker-assisted selection as a strategy for increasing the probability of selecting superior genotypes. *Crop Science*.

[456] Schneider KA, Brothers ME, Kelly JD (1997). Marker-assisted selection to improve drought resistance in common bean. *Crop Science*.

[457] Stuber CW, Polacco M, Senior ML (1999). Synergy of empirical breeding, marker-assisted selection, and genomics to increase crop yield potential. *Crop Science*.

[458] Tar'an B, Michaels TE, Pauls KP (2003). Marker-assisted selection for complex trait in common bean (*Phaseolus vulgaris* L.) using QTL-based index. *Euphytica*.

[459] Toojinda T, Baird E, Booth A (1998). Introgression of quantitative trait loci (QTLs) determining stripe rust resistance in barley: an example of marker-assisted line development. *Theoretical and Applied Genetics*.

[460] Zhou PH, Tan YF, He YQ, Xu CG, Zhang Q (2003). Simultaneous improvement for four quality traits of Zhenshan 97, an elite parent of hybrid rice, by molecular marker-assisted selection. *Theoretical and Applied Genetics*.

[461] Zhu H, Briceño G, Dovel R (1999). Molecular breeding for grain yield in barley: an evaluation of QTL effects in a spring barley cross. *Theoretical and Applied Genetics*.

[462] Stuber CW, Edward MD (1986). Genotypic selection for improvement of quantitative traits in corn using molecular marker loci.

[463] Edwards M, Johnson L RFLPs for rapid recurrent selection.

[464] Lecomte L, Duffé P, Buret M, Servin B, Hospital F, Causse M (2004). Marker-assisted introgression of five QTLs controlling fruit quality traits into three tomato lines revealed interactions between QTLs and genetic backgrounds. *Theoretical and Applied Genetics*.

[465] Grandillo S, Ku H-M, Tanksley SD (1999). Identifying the loci responsible for natural variation in fruit size and shape in tomato. *Theoretical and Applied Genetics*.

[466] Bogdanove AJ (2002). *Pto* update: recent progress on an ancient plant defence response signalling pathway. *Molecular Plant Pathology*.

[467] Gu Y-Q, Martin GB (1998). Molecular mechanisms involved in bacterial speck disease resistance of tomato. *Philosophical Transactions of the Royal Society of London B: Biological Sciences*.

[468] Martin GB, Frary A, Wu T (1994). A member of the tomato *Pto* gene family confers sensitivity to fenthion resulting in rapid cell death. *The Plant Cell*.

[469] Grierson D, Tucker GA, Keen J, Ray J, Bird CR, Schuch W (1986). Sequencing and identification of a cDNA clone for tomato polygalacturonase. *Nucleic Acids Research*.

[470] Tanksley SD (2004). The genetic, developmental, and molecular bases of fruit size and shape variation in tomato. *The Plant Cell*.

[471] van der Knaap E, Tanksley SD (2001). Identification and characterization of a novel locus controlling early fruit development in tomato. *Theoretical and Applied Genetics*.

[472] Alpert KB, Tanksley SD (1996). High-resolution mapping and isolation of a yeast artificial chromosome contig containing *fw2.2*: a major fruit weight quantitative trait locus in tomato. *Proceedings of the National Academy of Sciences of the United States of America*.

[473] Cong B, Liu J, Tanksley SD (2002). Natural alleles at a tomato fruit size quantitative trait locus differ by heterochronic regulatory mutations. *Proceedings of the National Academy of Sciences of the United States of America*.

[474] Liu J, Cong B, Tanksley SD (2003). Generation and analysis of an artificial gene dosage series in tomato to study the mechanisms by which the cloned quantitative trait locus *fw2.2* controls fruit size. *Plant Physiology*.

[475] Ling H-Q, Koch G, Bäumlein H, Ganal MW (1999). Map-based cloning of *chloronerva*, a gene involved in iron uptake of higher plants encoding nicotianamine synthase. *Proceedings of the National Academy of Sciences of the United States of America*.

[476] Mao L, Begum D, Chuang H-W (2000). *JOINTLESS* is a MADS-box gene controlling tomato flower abscission zone development. *Nature*.

[477] Mao L, Begum D, Goff SA, Wing RA (2001). Sequence and analysis of the tomato *JOINTLESS* locus. *Plant Physiology*.

[478] Budiman MA, Chang S-B, Lee S (2004). Localization of jointless-2 gene in the centromeric region of tomato chromosome 12 based on high resolution genetic and physical mapping. *Theoretical and Applied Genetics*.

[479] Dixon MS, Jones DA, Keddie JS, Thomas CM, Harrison K, Jones JDG (1996). The tomato *Cf-2* disease resistance locus comprises two functional genes encoding leucine-rich repeat proteins. *Cell*.

[480] Rivers BA, Bernatzky R, Robinson SJ, Jahnen-Dechent W (1993). Molecular diversity at the self-incompatibility locus is a salient feature in natural populations of wild tomato (*Lycopersicon peruvianum*). *Molecular and General Genetics*.

[481] Adkins S (2000). Tomato spotted wilt virus—positive steps towards negative success. *Molecular Plant Pathology*.

[482] Brommonschenkel SH, Frary A, Frary A, Tanksley SD (2000). The broad-spectrum tospovirus resistance gene *Sw-5* of tomato is a homolog of the root-knot nematode resistance gene *Mi*. *Molecular Plant-Microbe Interactions*.

[483] Brommonschenkel SH, Tanksley SD (1997). Map-based cloning of the tomato genomic region that spans the *Sw-5* tospovirus resistance gene in tomato. *Molecular and General Genetics*.

[484] Wang Y, van der Hoeven RS, Nielsen R, Mueller LA, Tanksley SD (2005). Characteristics of the tomato nuclear genome as determined by sequencing undermethylated *EcoR*I digested fragments. *Theoretical and Applied Genetics*.

[485] Oldroyd GED, Staskawicz BJ (1998). Genetically engineered broad-spectrum disease resistance in tomato. *Proceedings of the National Academy of Sciences of the United States of America*.

[486] Remington DL, Ungerer MC, Purugganan MD (2001). Map-based cloning of quantitative trait loci: progress and prospects. *Genetical Research*.

[487] Khalf-Allah AM, Mousa AG (1972). Relative importance of types of gene action for early yield, total yield and fruit size in tomato. *Egyptian Journal of Genetics and Cytology*.

[488] Ibarbia EA, Lambeth VN (1969). Inheritance of soluble solids in a large/small-fruited tomato cross. *Journal of the American Society of Horticultural Science*.

[489] Wang Y, van der Hoeven R, Nielsen R Characteristics of the tomato nuclear genome as determined by sequencing unmethylated DNA and
euchromatic and pericentromic BACs.

[490] Khush GS, Rick CM (1968). Cytogenetic analysis of the tomato genome by means of induced deficiencies. *Chromosoma*.

[491] Mueller LA, Solow TH, Taylor N (2005). The SOL genomics network. A comparative resource for Solanaceae biology and beyond. *Plant Physiology*.

